# Controlling Antimicrobial Activity of Quinolones Using Visible/NIR Light-Activated BODIPY Photocages

**DOI:** 10.3390/pharmaceutics14051070

**Published:** 2022-05-17

**Authors:** Elena Contreras-García, Carmen Lozano, Cristina García-Iriepa, Marco Marazzi, Arthur H. Winter, Carmen Torres, Diego Sampedro

**Affiliations:** 1 Departamento de Química, Centro de Investigación en Síntesis Química (CISQ), Universidad de La Rioja, Madre de Dios 53, 26006 Logroño, Spain; elena.contreras@unirioja.es; 2 Área Bioquímica y Biología Molecular, Universidad de La Rioja, Madre de Dios 53, 26006 Logroño, Spain; carmen.lozano@unirioja.es (C.L.); carmen.torres@unirioja.es (C.T.); 3 Departamento de Química Analítica, Química Física e Ingeniería Química, Grupo de Reactividad y Estructura Molecular (RESMOL), Universidad de Alcalá, 28805 Alcalá de Henares, Spain; cristina.garciai@edu.uah.es (C.G.-I.); marco.marazzi@uah.es (M.M.); 4 Instituto de Investigación Química “Andrés M. del Río” (IQAR), Universidad de Alcalá, 28805 Alcalá de Henares, Spain; 5 Department of Chemistry, Iowa State University, Ames, IA 50014, USA; winter@iastate.edu

**Keywords:** photodrugs, quinolones, BODIPYs, antibiotics, photoprotecting groups, photocages

## Abstract

Controlling the activity of a pharmaceutical agent using light offers improved selectivity, reduction of adverse effects, and decreased environmental build-up. These benefits are especially attractive for antibiotics. Herein, we report a series of photoreleasable quinolones, which can be activated using visible/NIR light (520–800 nm). We have used BODIPY photocages with strong absorption in the visible to protect two different quinolone-based compounds and deactivate their antimicrobial properties. This activity could be recovered upon green or red light irradiation. A comprehensive computational study provides new insight into the reaction mechanism, revealing the relevance of considering explicit solvent molecules. The triplet excited state is populated and the photodissociation is assisted by the solvent. The light-controlled activity of these compounds has been assessed on a quinolone-susceptible *E. coli* strain. Up to a 32-fold change in the antimicrobial activity was measured.

## 1. Introduction

Antibiotics are a powerful instrument for treating diseases caused by bacteria, both in humans and in animals. However, the benefits of these drugs are also accompanied by some serious drawbacks, such as the emergence and spread of antibiotic-resistant bacteria [1,2,3]. Furthermore, systemic delivery of antibiotics to treat localized infections destroys beneficial bacteria in the patient, such as intestinal bacteria (that could be susceptible to the antibiotic), which can also lead to numerous side effects.

From a chemical point of view, solutions to these problems include research of new antimicrobial agents or new administration methods. Among these new routes of administration, light-controlled drugs are becoming an emerging field [4,5,6]. Light-activated drugs could solve some of the problems of traditional pharmacological agents, such as poor selectivity and the appearance of adverse effects. The activation process of these drugs can be reversible or irreversible depending on the specific type of photochemical reaction. Gaining control over a molecule in a reversible way can be accomplished with the use of molecular switches [4,7,8]. In fact, different types of molecular switches have been already employed to create systems that modulate the activity of antibacterial molecules [9,10].

On the contrary, irreversible strategies to activate a pharmacological agent are based on the cage strategy [11,12]. These molecules contain a photoreactive moiety covalently attached to an active compound in a way that renders the molecule inactive to its target receptor. The light-sensitive part of the molecule, also known as photoreleasable protecting group (PPG), provides spatial and temporal control over the bioactivity. Attaching a PPG to a pharmacological agent allows the controlled photorelease of the active form in a specific target. The active drug will be then released exactly in the place and at the moment in which it is required. Consequently, the drug would be in its inactive form in the rest of the body, where its effect could be damaging. This would remarkably improve the selectivity of the drug, which would allow, in turn, a better adjustment in drug dosage, making possible the use of higher doses in the affected part and removing the adverse effects in other healthy parts. In addition, only small fractions of the active drug will face the excretion process, which would decrease environmental build-up [11,12]. Concerning antibiotics, all of these advantages could have an immense impact on the emergence and spread of antibiotic-resistant bacteria as well as the useful life of antimicrobial agents available on the market, which could be potentially extended.

Regarding the PPG part of the light-activated drugs, several structures have been employed as photocages, including nitrobenzyl, nitroanilide, or phenacyl moieties [13]. However, most of them are mainly responsive to UV light. Unfortunately, UV light is damaging to living tissues [14,15]. In addition, this type of light barely offers tissue penetration [16,17]. Thus, these types of PPGs have serious drawbacks that should be considered for biological applications. More recently, new PPGs have been reported absorbing in the visible region, such as coumarins [18,19], quinones [20,21], and boron-dipyrromethenes, also known as BODIPYs [22,23,24,25].

Perhaps one of the most studied photocages in the last years are BODIPYs. Despite being a relatively new type of PPG, meso-substituted BODIPYs have been already employed in drug release to achieve spatial and temporal control of some pharmacological agents [26,27]. This family of chromophores offers tunable absorptions throughout the visible/near-IR region (450–800 nm), high molar absorption coefficients (30,000–80,000 M^−1^ cm^−1^), and known biological compatibility [22]. Thus, BODIPYs consists of a new and promising alternative for the design and use of PPGs in biological media. The optical properties of these compounds (strong and tunable absorption in the red and infrared regions of the spectrum) make them perfect candidates for the photorelease of drugs in biological media as the required light for this photoreaction has an increased tissue penetration and it is completely innocuous to living organisms. 

Having these excellent properties in mind, we decided to design a series of molecules combining a BODIPY moiety (acting as PPG) and a quinolone antibiotic. It is well known that the acid group in position 3 of the quinolone core for this family of compounds has a crucial role in the antimicrobial activity of the molecule [28]. This group is required for the binding to the gyrase complex, critical for the activity of these compounds and for their antimicrobial activity. Hence, we created an irreversible system by incorporating the PPG in that position. We envisioned a strong deactivation of the antimicrobial properties once a photoactivatable moiety was attached to the quinolone structure in position 3. Doing so, the necessary free acid group would be blocked, rendering inactive the quinolone derivative. Subsequently, after irradiation of the molecule and release of the antibiotic, the activity should be recovered. Among the different alternatives for PPGs blocking position 3, we selected BODIPYs due to their optical properties which seem particularly appealing for biological applications, in strong contrast with most of the reported PPGs.

Beyond the optical properties of these photocages, water solubility is also a critical feature for biological applications. Any compound designed to be used in biological media needs to feature a reasonable water solubility or, at least, to be soluble in compatible solvents. In this respect, BODIPYs, as aromatic organic compounds present very low water solubility. Thus, the design of compounds with potential antimicrobial activity based on these PPGs should include chemical modifications which increase this value. Due to the typically low concentrations required in clinical practice for antimicrobial agents, even modest values for solubility in water or compatible solvents could be enough.

Herein, we report our efforts in the design, synthesis, photochemical characterization, computational exploration and biological use of new dyads prepared from modified BODIPYs carrying a photo-releasable quinolone derivative, activatable by visible light.

## 2. Materials and Methods

The minimal inhibitory concentration (MIC) is defined as the lowest concentration of an antimicrobial that inhibits the visible growth of a microorganism after overnight incubation. MIC values were determined by the microdilution method in cation adjusted Mueller-Hinton broth (Becton Dickinson). *Escherichia coli* ATCC 25922 strain was cultured in Brain-Heart Infusion (BHI) agar (Becton, Dickinson and Company Sparks, Le Pont de Claix, France) for 24 h at 37 °C. Overnight colonies of a pure culture on agar were used to prepare an inoculum adjusted to the turbidity of a 0.5 McFarland in sterile saline solution (1 × 10^8^ CFU/mL). This suspension was diluted in cation adjusted Mueller-Hinton broth until a density of 5 × 10^5^ CFU/mL and exposed to a serial twofold dilution of each compound tested (0.03–32 mg/L) with a final volume of 0.1 mL in a 96 well microdilution tray. The MIC value was determined as the lowest concentration of compound where no visible growth was apparent. Positive (with bacteria and cation adjusted Mueller-Hinton broth) and negative (only with cation adjusted Mueller-Hinton broth) control wells were included in all assays. Moreover, control tests of solvents (mixtures of water and DMSO 40:60 or 20:80) were included to check the survival of the bacterium in these concentrations.

## 3. Results and Discussion

**Figure 1:**
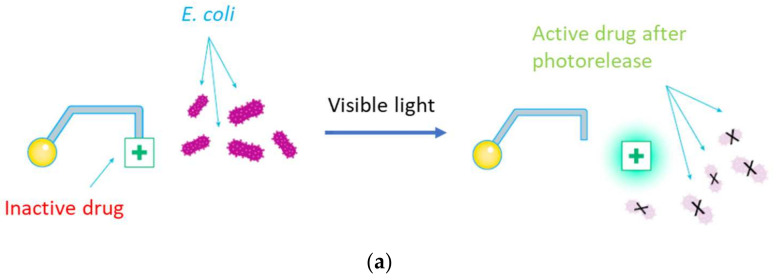

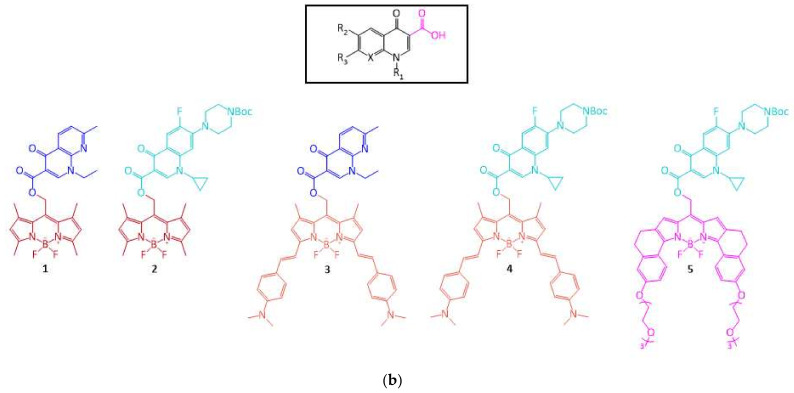
(**a**) Conceptual representation of drug photorelease. (**b**) On top, general structure of the quinolone antibiotics employed, position 3 required for activity is highlighted in pink. Bottom, structure of the PPG-quinolone dyads (**1**–**5**). The color key represents the different moieties included in this study. Already reported BODIPYs shown in red and orange, new BODIPY in purple. Antibiotics shown in dark blue (nalidixic acid) and light blue (ciprofloxacin).

Our main goal was to create a system compatible with biological applications. This should imply a photorelease upon irradiation with visible light of compounds with a reasonable water solubility. With this objective in mind, we selected three BODIPY scaffolds (see Figure 1) and combined them with two different antibiotics to prepare five different PPG-drug dyads. Two of the BODIPY moieties have been previously reported (PPG parts in dyads **1**–**4**) [22]. In addition, a new PPG was specifically designed and prepared in this study with the aim of increasing the water solubility in the dyad **5**. As the antibiotic parts, two different compounds have been chosen, nalidixic acid (in **1**, **3**) and ciprofloxacin (in **2**, **4**, **5**). Combination of the drugs with the BODIPY-based PPGs allowed for the design of the five compounds shown in Figure 1. Dyad **5** was designed to combine the higher antibiotic activity of ciprofloxacin with the expected improved water solubility produced by the newly designed PPG.

**Figure 2:**
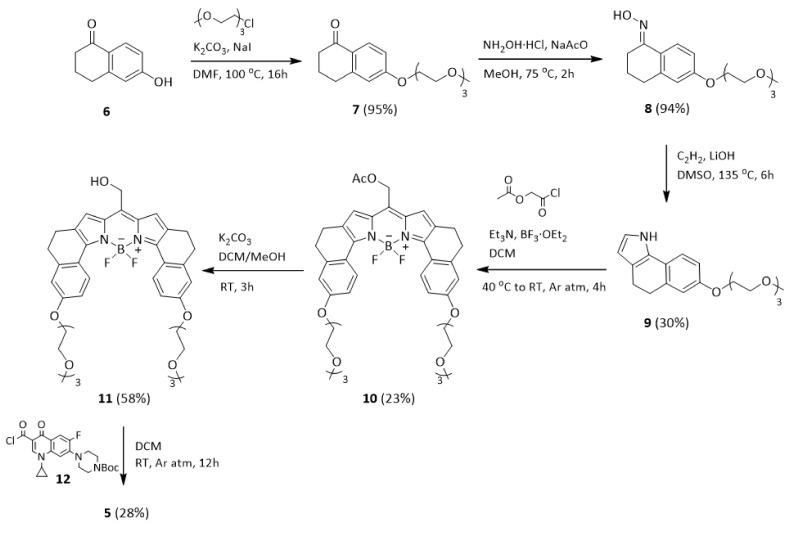
Synthetic route of photocage **11** and the corresponding protected quinolone **5**.

For the synthesis of BODIPY photocages for compounds **1**, **2**, **3**, and **4**, we based our route on a previously described procedure [22]. However, a modification in the preparation was needed to obtain the final protected product. Specifically, we synthesized the acyl chloride of the antibiotic, either nalidixic acid or BOC-ciprofloxacin, and we made the coupling reaction with the corresponding BODIPY in the presence of a base (see Supporting Information, Figures S1 and S2). For compound **5** and its corresponding photocage, a new synthetic route was designed (Figure 2). First, a glycol chain was introduced in tetralone **6**, through an O-alkylation to get derivative **7**. Next, oxime **8** was obtained by condensation of **7** with hydroxylamine. Then, pyrrole **9** was formed via a Trofimov reaction. The synthesis of the BODIPY **10** was carried out following the standard methodology [22]. After that, conversion to the meso-methylhydroxy BODIPY **11** was achieved upon ester hydrolysis. Finally, the coupling reaction between the acyl chloride form of BOC-ciprofloxacin and **11** gave the protected form of the antibiotic **5**. More details on the synthesis of these compounds and complete characterization data are available in the Supporting Information.

**Figure 3:**
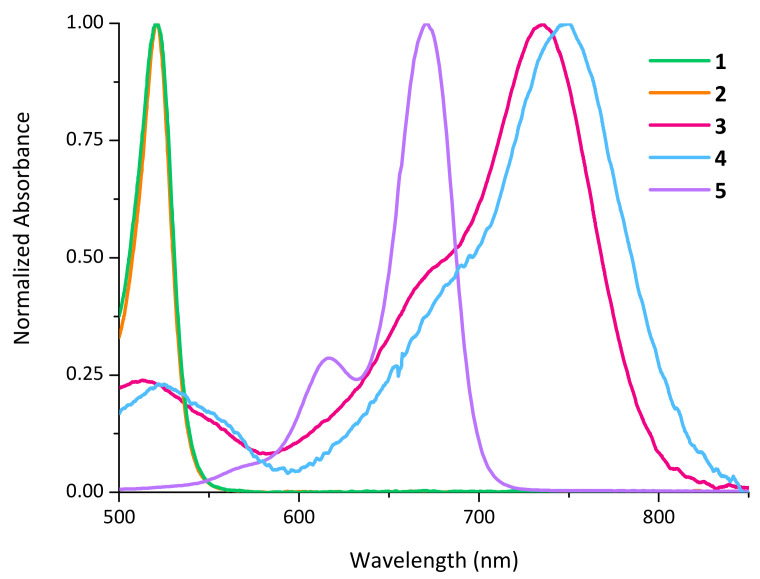
UV-Vis spectra of protected quinolones (**1**–**5**). All compounds were dissolved in DCM in a concentration close to 5 × 10^−5^ M.

Once we prepared and characterized the compounds, we studied their photochemical properties. First, we recorded their electronic absorption spectra. As expected, all of them absorb in the visible region, but more interestingly, three of them inside the optical therapeutic window (650–1350 nm, see Figure 3 and Table 1) in which there is a maximum depth of tissue penetration. Dyads **1** and **2** bearing the BODIPY with a simpler structure displayed absorption in the green region of the spectrum, both with the maximum absorption at 520 nm. A large bathochromic shift took place when the conjugation of the BODIPY part was elongated through the addition of two (dimethylamino)-styryl groups. Compounds **3** and **4**, bearing this modified BODIPY moieties displayed absorption in the red/NIR region of the spectrum with the maximum absorption at 735 and 744 nm, respectively. Finally, compound **5** displayed absorption in the red region of the spectrum with the maximum at 671 nm. The excitation energies and electronic nature of the vertical transition have been studied computationally for compounds **2**, **4** and **5** to account for the differences cause by the BODIPY moiety. Regarding the excitation energies, the same qualitative trend has been obtained: compound **2** is significantly blue-shifted compared to **4** and **5**, being compound **4** the one showing the more red-shifted absorption (Table S4). The electronic nature of the S_0_ → S_1_ vertical transition is characterized by a ^1^ (π,π*) transition localized in the BODIPY scaffolds, independently of the considered solvent. Hence, we can confidently conclude that the antibiotic moiety is not involved in the electronic transition (Figure S10). With these results in hand, we proceeded to choose for each case the adequate source of irradiation in order to test if these compounds were indeed photoreactive. 

Compounds **1** and **2** were irradiated with a 30 W RGB LED in green mode (λ_max_ = 520 nm), dyad **5** was irradiated in red mode (λ_max_ = 635 nm), and compounds **3** and **4** were irradiated with a 515 mW far-red lamp (λ_max_ = 730 nm, see Figure S9 for details). Upon irradiation, the release reaction took place until completion in all cases, and no alternative products were observed by HPLC (see Figures S3–S7, Supporting Information). Additionally, the stability of the compounds at room temperature was measured. The samples were kept in the dark for 5 days, and no release of quinolones or other decomposition products were observed by ^1^H-NMR or HPLC after that time.

Then, we measured the quantum yield of the release process at the different wavelengths for each compound, to evaluate the photorelease efficiency with the visible light sources previously employed. We measured the quantum yield instrumentally using a protocol already reported [29]. The photonic flux emitted by the light source, as well as the number of photons not absorbed by the sample, were determined using an Ocean Optics USB4000-UV-Vis detector equipped with a cosine corrector (see Figure S8). The values obtained showed a better release efficiency of compounds **1**, **2**, and **5**. The obtained results are in line with previously reported photorelease quantum yield for BODIPYs [22]. The low quantum yields for the photorelease is a common drawback for BODIPY PPGs [22]. However, these molecules can fully release the antibiotic molecule with Vis/NIR light, and the quantum efficiencies (Φε) are similar to nitrobenzyl photocages because of a much larger extinction coefficient of the BODIPY chromophore compared to the nitrobenzyl chromophore. 

**Figure 4:**
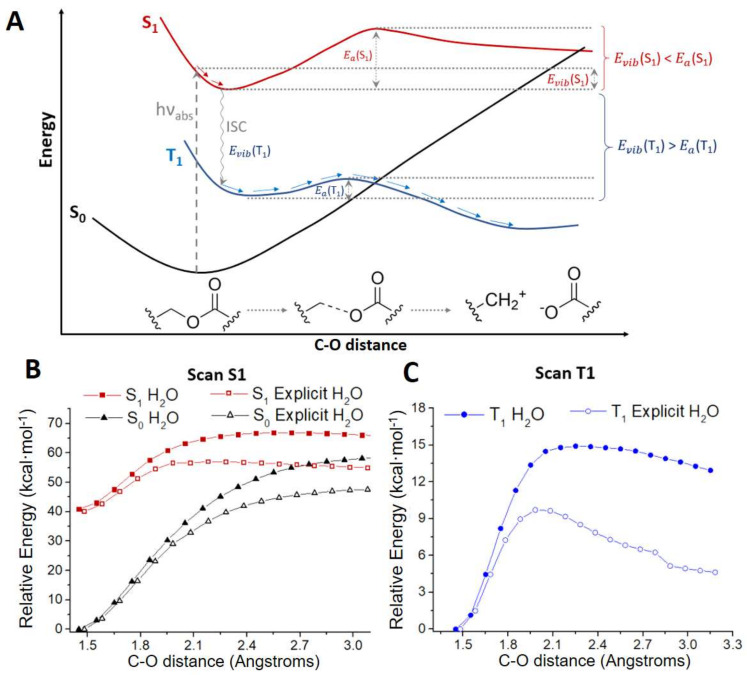
(**A**) General scheme of the photorelease mechanism computed for compounds **2**, **4** and **5**. (**B**) Scan computed in S_1_ and (**C**) T_1_ along the C-O distance considering the solvent (water) implicitly (filled symbols) or explicitly (empty symbols).

To gather a deeper insight into the photorelease mechanism, we have studied by means of computational methods under the framework of the Time-Dependent Density Functional Theory (TD-DFT, see Computational Methods section for details) the antibiotic release pathway for compounds **2**, **4** and **5**. Previous reports [30] have shown that C-O bond breaking in the triplet state may be a key step in the photocleavage mechanism for related compounds. For all the computed structures, a similar scheme has been found (Figure 4A). In particular, after excitation to S_1_ the system reaches a minimum in this state. From there, the S_1_ path along the C-O bond breaking responsible of the photorelease has been scanned (Figure 4B), finding a significantly large energy barrier compared to the excess energy obtained after excitation (red curve in Figure 4A). In particular, the energy barriers in S_1_ computed for **2**, **4** and **5** when including explicit water molecules as solvent are ca. 15 kcal/mol (Figure 4B and Figure S11). Hence, the photorelease in S_1_ is not an energetically favorable path. However, if the system is trapped in the S_1_ minimum, it could also populate the first triplet state (T_1_) which lies lower in energy (Table S5), through an intersystem crossing (ISC) as shown in Figure 4A. Once in T_1_, the system evolves to a minimum from which it could complete the C-O bond breaking overcoming a barrier whose computed energy is lower than the one computed in S_1_ (Figure 4C). In particular, the energy barrier computed in T_1_ is <10 kcal/mol for compounds **2**, **4** and **5** (Figure 4C and Figure S12) when considering explicit water as solvent. The transition states corresponding to these energy barriers have been optimized, hence describing mechanistically the antibiotic release (Figure S13). In addition, this T_1_ barrier could be easily overcome by the system as the excess energy obtained after the ISC is larger than the T_1_ energy barrier (blue curve in Figure 4A). Hence, we can conclude that the photorelease of the antibiotic should take place preferentially in T_1_. This conclusion is in agreement with previous studies focused on other families of BODIPY-based photocages which found the photorelease in the triplet state to be the main path [30].

**Figure 5:**
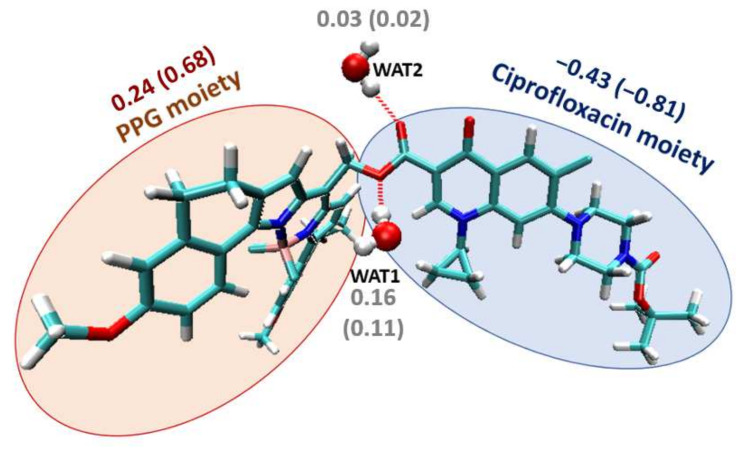
Moieties considered for charge analysis (depicting as an example the structure of **5**), showing the explicit water molecules and their hydrogen-bond interactions (dotted red lines). The charge of each moiety computed at the T_1_ minimum (T_1_ transition state) are also depicted.

Moreover, we have evaluated the solvent effect on the mechanism. Photocleavage has been suggested to be highly affected by the solvent environment [30]. The solvent may contribute by trapping the generated carbocation upon light irradiation or by generating a solvent-caged ion pair. In order to test the solvent effect on these compounds, we have computed the photorelease path considering three different implicit continuum solvents: dichloromethane, DMSO and water. Clearly, the ability of these solvents to stabilize carbocations or ion pairs would be different. For all compounds under study, it is observed that DMSO and water give almost identical profiles whereas the ones computed in dichloromethane are slightly different, showing larger energy barriers, both in S_1_ and T_1_ (Figures S11 and S12). In addition, we have computed these paths considering two explicit water molecules, leading to two hydrogen bond interactions with the oxygen atoms of the compound (Figure 5) while keeping the implicit description for the surroundings. A clear and significate effect of these explicit water molecules has been observed as the energy barriers computed both in S_1_ and T_1_ are reduced between 5 and 10 kcal/mol depending on the electronic state and the compound (Figure 4B,C, Figures S11 and S12). 

In the light of the results obtained when considering explicit water molecules, we have analyzed the charge evolution along the release path to check their role. For this aim, we have calculated the charges of ciprofloxacin and the PPG for the stationary points in T_1_ (minimum, transition state and products) considering both implicit and explicit water (Table S6). By analyzing the data, we verify that the antibiotic release generates the negatively charged ciprofloxacin moiety and the carbocation of the corresponding PPG moiety. Remarkably, it is observed that one of the explicit water molecules, in particular WAT1 (Figure 5), has a significant positive charge along the release path. That is, this water molecule is not a passive spectator, but it acts as a nucleophile promoting the C-O breaking, and thus lowering the T_1_ energy barrier. Hence, the water molecule (or probably any nucleophilic solvent) is contributing to the reaction mechanism by lowering the required activation energy. These results clearly suggest the relevance of explicit solvent molecules for the computational study of BODIPYs photorelease, in accordance with the experimental requirement for specific solvents to speed up the release process [22].

**Figure 6:**
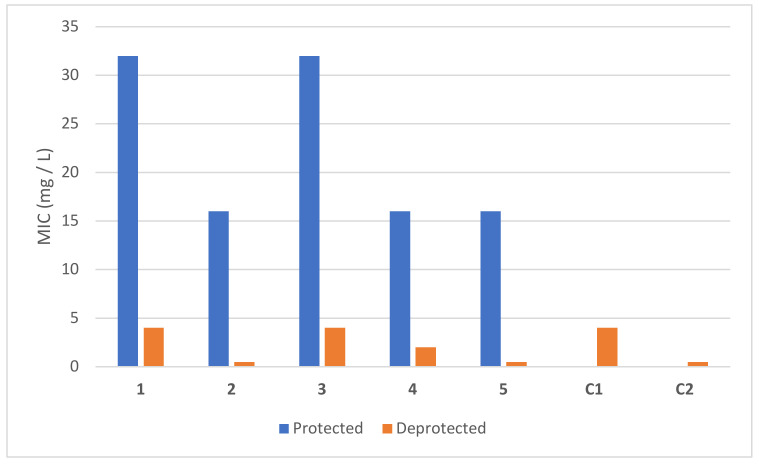
Graphic comparison of MIC values on *E. coli* ATCC 25922 strain of the protected and deprotected forms of the photoreleasable quinolones **1**–**5**. In blue it is represented the protected form (BODIPY linked to the antibiotic). In orange it is shown the activated form (after irradiation of the sample, which induces the release of the antibiotic). C1 and C2 refer to the quinolones’ control experiments (C1 = nalidixic acid, C2 = BOC-ciprofloxacin) in 60:40 DMSO:H_2_O. Note that in the case where MIC value is >32 mg/L, a value of 32 has been represented, and for the compound with MIC 8–16 mg/L, the higher value has been depicted. These experiments were performed in triplicate and the same result was obtained in all experiments.

**Figure 7:**
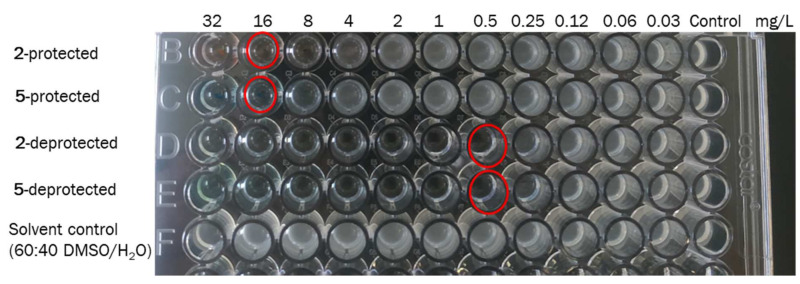
MIC study on *E. coli* ATCC 25922 of **2** and **5**. The wells corresponding to the minimum concentrations of compounds 2 and 5 in which no bacterial growth was detected (MIC values) are marked with a red circle. The protected compounds showed higher MIC values than the deprotected ones, indicating that the antibiotic regains its activity upon light irradiation. The solvent control did not affect bacterial growth. Green light (100 W, 520 nm for compound **2**) or red light (50 W, 625 nm for compound **5**) were used for 1 h.

Once we proved the ability of BODIPYs as photocages of quinolone antibiotics, we tested the effect they have on the antimicrobial activity before and after irradiation (see Table 2, Figure 6 and Figure 7). Firstly, the compounds were irradiated to ensure their complete photorelease. After that, an overnight culture of the quinolone susceptible *Escherichia coli* ATCC 25922 strain [31] was used to prepare an inoculum adjusted to the turbidity of a 0.5 McFarland in sterile saline (equivalent to 1 × 10^8^ CFU/mL). This suspension was diluted in cation adjusted Mueller-Hinton broth to give a final organism density of 5 × 10^5^ CFU/mL and was exposed to different concentrations (0.03–32 mg/L) either of the protected forms **1**–**5** (non-irradiated compounds: BODIPY linked to the antibiotic) or the deprotected form (compounds previously irradiated where the antibiotic was already released) with a final volume of 0.1 mL in a 96 well microdilution tray. The microtiter plate was incubated at 37 °C for 24 h in the dark. This experiment was performed in triplicate and the same result was obtained in all experiments.

For this study, stock samples were dissolved in mixtures of water and DMSO (40:60 for **1**, **2**, and **5**, 20:80 for **3** and **4**). Subsequent serial two-fold dilutions to achieve lower concentrations were made with cation adjusted Mueller Hinton broth. A control test of solvents was made to check the survival of the bacterium in the water/DMSO mixtures used. Solvents were found to not affect the bacterium growth as both mixtures employed (80:20 and 60:40) resulted in MIC > 32 mg/L. In addition, the antimicrobial activity of both quinolones, nalidixic acid and BOC-ciprofloxacin, was tested as a reference (see Figure 6). 

The derivatives of nalidixic acid **1** and **3** showed an 8-fold change in activity when they were irradiated (MIC**_1-_**_protected_ > 32 mg/L vs. MIC**_1-_**_deprotected_ = 4 mg/L, MIC**_3-_**_protected_ = 32 mg/L vs. MIC**_3_**_-deprotected_ = 4 mg/L). These values were in agreement with the control of nalidixic acid, which reported a value of 4 mg/L, indicating that after irradiation, the antibiotic was completely released.

BOC-ciprofloxacin derivatives showed a 32-fold difference in activity in **2** and **5** (MIC**_2_**_-protected_ = 8–16 mg/L vs. MIC**_2_**_-deprotected_ = 0.5 mg/L, MIC**_5_**_-protected_ = 16 mg/L vs. MIC**_5_**_-deprotected_ = 0.5 mg/L), and 8-fold change in **4** (MIC**_4_**_-protected_ = 16 mg/L vs. MIC**_4_**_-deprotected_ = 2 mg/L). These results were in agreement with the controls made for BOC-ciprofloxacin which reported MIC values of 0.5 and 2 mg/L when the stock solution (256 mg/L) was dissolved in 60:40 and 80:20 DMSO:H_2_O respectively. The values once again demonstrated that upon irradiation the release yield of the antibiotic was 100%.

We then decided to perform the antibiotic’s release in presence of the bacteria (Figure 7), to check the antimicrobial activity. For this experiment, we selected two of the protected compounds **2** and **5**, since they showed a bigger change in activity upon irradiation (Table 2). Like the previous experiment, 5 × 10^5^ CFU/mL of *E. coli* ATCC 25922 strain was exposed to different concentrations (0.03–32 mg/L) of the protected form of these compounds. Control tests of solvents and BOC-ciprofloxacin were also included. The 96 well microdilution trays were incubated at 37 °C for 24 h. During this time, the plates were irradiated for a period of 1 h with green light in the case of **2**, and red light for **5**.

The difference found in the MIC values shown in Table 2 and Table 3 are due to the different experimental conditions. However, it was shown that the release process could be successfully induced also in a biological environment, although a smaller antimicrobial activity was observed in this case. The experimental irradiation conditions (see Figure S9) implied the irradiation of the well plates from the bottom with the corresponding LED, green for **2**, and red for **5**. Therefore, the light was not directly applied to the sample, but it went through the well plate material, which allows for a ca. 60% of light transmittance. Thus, light intensity effectively reaching the sample is lower. Besides, the culture medium is non-transparent, and as the bacteria grows, turbidity appears, which also implies a decrease in the amount of light that reaches the photoactive compound. Moreover, while the release process takes place, the bacteria are being incubated and therefore keep growing. This implies a different time scale between results shown in Table 2 and Table 3. These practical constraints are due to the set-up used and we plan to modify it in the following studies. However, these experiments clearly manifest that we could use the prepared compounds in a biological environment.

## 4. Conclusions

We have synthesized several photoreleasable derivatives of quinolones. All of them absorb in the visible region, including three dyads with absorbance inside the optical therapeutic window. The BODIPY scaffold has been linked at position 3 of the quinolones, which is key for the antimicrobial role of the molecule. The activation process can be accomplished in an adequate time scale that could allow for the external control of the bacterial growth. The molecules presented herein have the required solubility in biologically inert mixtures of water and DMSO, which allowed us to study the antimicrobial properties before and after irradiation. We have proved that the photocage has an important deactivation effect in the molecule’s activity, with the better results showing a 32-fold change in activity. In addition, we have calculated the photorelease mechanism in different environments, furnishing arguments in favor of T_1_ population followed by photodissociation. The inclusion of explicit solvent molecules in the calculations appears to be a requisite for the correct description of this process. To sum up, we have created an irreversible system for the activation of antibiotics controlled by Vis/NIR light. We have demonstrated that the release process takes place in a biological environment by performing in vitro studies in the presence of bacteria. These results open the possibility for the future applications of these types of drugs in systems that are more complex.

## Figures and Tables

**Table 1:** Photochemical properties of protected quinolones.

Compound	λ_max_ (nm)	ε_max_ (M^−1^ cm^−1^)	Ф (%)
**1**	520	60,500	0.006 ^a^
**2**	520	56,200	0.006 ^a^
**3**	735	58,400	0.003 ^b^
**4**	744	52,900	0.003 ^b^
**5**	671	80,500	0.006 ^c^

Photorelease quantum yields (Ф) determined instrumentally at 520 ^a^, 730 ^b^, and 625 ^c^ nm.

**Table 2:** Minimum Inhibitory Concentration (MIC) values of photoreleasable quinolones before and after irradiation.

Compound	1 ^a^	2 ^a^	3 ^b^	4 ^b^	5 ^a^
MIC_protected_ (mg/L)	>32	8–16	32	16	16
MIC_deprotected_ (mg/L)	4	0.5	4	2	0.5

^a^ In water/DMSO (40:60). ^b^ In water/DMSO (20:80).

**Table 3:** MIC values for the deprotected forms obtained in the presence of *E. coli* ATCC 25922 with and without irradiation. Green light (100 W, 520 nm for compound **2**) or red light (50 W, 625 nm for compound **5**) were used for 1 h.

Compound	MIC (mg/L) with Irradiation	MIC (mg/L) without Irradiation
**2**	2	8
**5**	4	16
**BOC-ciprofloxacin**	0.5	0.5

## Data Availability

Not applicable.

## Supplementary Materials

The following supporting information can be downloaded at: https://www.mdpi.com/article/10.3390/pharmaceutics14051070/s1. Synthesis (Figure S1. Synthetic route of compounds **1** and **3**, photoreleasable derivatives of nalidixic acid. Figure S2. Synthetic route of compounds **2** and **4**, photoreleasable derivatives of Boc-ciprofloxacin) [22,32], full characterization details for dyads **1**–**5**, experimental details for the HPLC (Table S1. HPLC method A, used for the analysis of compounds **1** and **2**. Table S2. HPLC method B, used for the analysis of compounds **3**, **4**, and **5**), photorelease (Table S3. Irradiation conditions of photoreleasable quinolones. Figure S3. Release process of compound **1** upon irradiation with green light. The peak corresponding to compound **1** decreases while the peak of nalidixic acid grows. Release yield > 95%. Figure S4. Release process of compound **2** upon irradiation with green light. The peak corresponding to compound **2** decreases while the peak of Boc-ciprofloxacin grows. Release yield > 90%. Figure S5. Release process of compound **3** upon irradiation with NIR light. The peak corresponding to compound **3** decreases while the peak of nalidixic acid grows. Release yield > 97%. Figure S6. Release process of compound **4** upon irradiation with NIR light. The peak corresponding to compound **4** decreases while the peak of Boc-ciprofloxacin grows. Release yield > 90%. Figure S7. Release process of compound **5** upon irradiation with red light. The peaks corresponding to compound **5** (in blue) gradually disappear while the peaks of Boc-ciprofloxacin (in green) grow. Release yield > 95%.), quantum yield (Figure S8. Schematic representation of the instrumental setup used to measure the quantum yield. The light emitted by the LED is focused through a lens and imaged into the sample equipped with a magnetic stirrer. The radiation not absorbed by the sample is collected by a cosine corrector.) [29] and MIC measurements (Figure S9. Irradiation of the photoreleasable quinolones in a biological environment. On the left, compound **2** irradiated with green light. On the right, compound **5** irradiated with red light.) [33], additional computational data (Table S4. Experimental and computed absorption (S_0_→S_1_) energies in eV (nm) and the corresponding oscillator strength (f), considering dichloromethane as the solvent. Figure S10. Molecular orbitals involved in the S_0_→S_1_ vertical transition of compound (A) **2**, (B) **4** and (C) **5**. Figure S11. Relaxed scan along the photorelease coordinate (distance C-O) in S_1_ considering diverse solvents for (A) compound **2**, (B) **4** and (C) **5**. Figure S12. Relaxed scan along the photorelease coordinate (distance C-O) in T_1_ considering diverse solvents for (A) compound **2**, (B) **4** and (C) **5**. Table S5. Energy of the first triplet (T_1_) and singlet excited state (S_1_) relative to the ground state (S_0_) computed at the S_1_ minimum. The results shown have been computed considering explicit water as the solvent. Figure S13. Transition state structures optimized in T_1_ considering explicit water molecules as the solvent. The vector of the negative frequency corresponds to the photorelease process. Table S6. Charge analysis for the ciprofloxacin, PPG and, when applying, the water molecules computed for the minimum, transition state and products in T_1_. The charge of each moiety is computed as the sum of the Mulliken charges of each atom included in the moiety. Table S7. S_1_-T_1_ spin-orbit coupling values (in cm^−1^) calculated at the CAM-B3LYP/def2-SVPD level of theory, including water as implicit solvent, without (implicit) or with (micro-solvation) two explicit water molecules.) [34,35,36,37,38,39,40,41] and Cartesian coordinates.
