# Supplementary material for: Controlling Antimicrobial Activity of Quinolones Using Visible/NIR Light-Activated BODIPY Photocages
Source: Pharmaceutics. 2022 May 17;14(5):1070. doi: 10.3390/pharmaceutics14051070 (PMC9144359; doi:10.3390/pharmaceutics14051070)
Supplement: Supplementary file 1 [file pharmaceutics-14-01070-s001.zip › pharmaceutics-1678222-supplementary.pdf]

Supporting information:

**Controlling Antimicrobial Activity of Quinolones Using Visible/NIR Light-Activated BODIPY Photocages**

Elena Contreras-García,[a] Carmen Lozano,[b] Cristina García-Iriepa,[c,d] Marco Marazzi,[c,d] Arthur H. Winter,[e] Carmen Torres,[b] and Diego Sampedro*[a]

[a]Departamento de Química, Centro de Investigación en Síntesis Química (CISQ), Universidad de La Rioja, Madre de Dios 53, 26006 Logroño, Spain

[b] Área de Bioquímica y Biología Molecular, Universidad de La Rioja, Madre de Dios 53, 26006 Logroño, Spain

[c] Universidad de Alcalá, Departamento de Química Analítica, Química Física e Ingeniería Química, Grupo de Reactividad y Estructura Molecular (RESMOL), Alcalá de Henares, Madrid, Spain

[d] Universidad de Alcalá, Instituto de Investigación Química ''Andrés M. del Río'' (IQAR), Alcalá de Henares, Madrid, Spain

[e]Department of Chemistry, Iowa State University, Ames, Iowa 50014, United States

## Contents

1. General information ........ 2
2. Synthesis ........ 3
3. HPLC Conditions ........ 12
4. Photorelease studies ........ 14
5. Quantum yield measurements ........ 20
6. Determination of minimal inhibitory concentration (MIC) ........ 22
7. Characterization data ........ 23
8. Computational Results ........ 31
9. Cartesian Coordinates ........ 36
10. References ........ 70

## 1. General information

All chemicals were obtained from commercial sources and used as received unless stated otherwise. Room temperature reactions were carried out between 22-25 °C. Thin-layer chromatography (TLC) was performed using silica gel 60 pre-coated aluminum plates (Macherey-Nagel 0.20 mm thickness) with a fluorescence indicator UV254. Solvents were ACS grade and used without further purification. For the detection of components, UV light at 254 nm was used. All chromatographic solvents were ACS grade and used without further purification. Column chromatography was performed on silica gel 60 (0.040-0.063 mm) and the indicated eluent in each case.

$^{1}H$ and $^{13}C$ NMR spectra were recorded on a Bruker AVANCE III HD (300 MHz), or Bruker AVANCE NEO (400 MHz) spectrometers at 25 °C. Chemical shifts (δ) are reported in parts per million (ppm) with the solvent resonance as the internal standard ($\delta_H$ 7.26 for $CHCl_3$, and 2.50 for DMSO-$d_6$; $\delta_C$ 77.16 for $CHCl_3$). Coupling constants (J) are reported in Hertz (Hz). The following abbreviations are used to indicate signal multiplicity: s (singlet), d (doublet), t (triplet), q (quartet), m (multiplet), dd (doublet of doublets), and quint (quintet).

Molecular absorption spectra were obtained by using a spectrometer provided with an Ocean Optics USB4000-UV-Vis detector and coupled to a deuterium/tungsten halogen lamp. Quartz cuvettes with 1 cm optical path length were used. Solutions were prepared in a concentration range of $5 \times 10^{-5}$ M approximately.

High-resolution electrospray mass spectra (HR ESI-MS) were recorded on a Bruker MicrOTOF-Q spectrometer. Accurate mass measurements were achieved by using sodium formate as an external reference. HR ESI-MS performed at Iowa State University were recorded on an Agilent QTOF 6540 spectrometer.

## 2. Synthesis

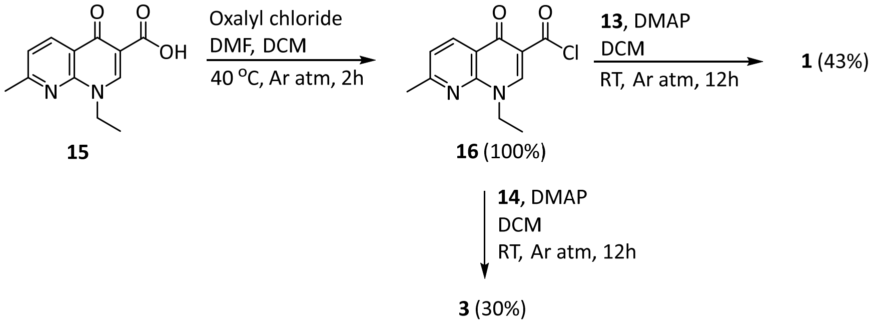


**Figure S1.** Synthetic route of compounds **1** and **3**, photoreleasable derivatives of nalidixic acid.

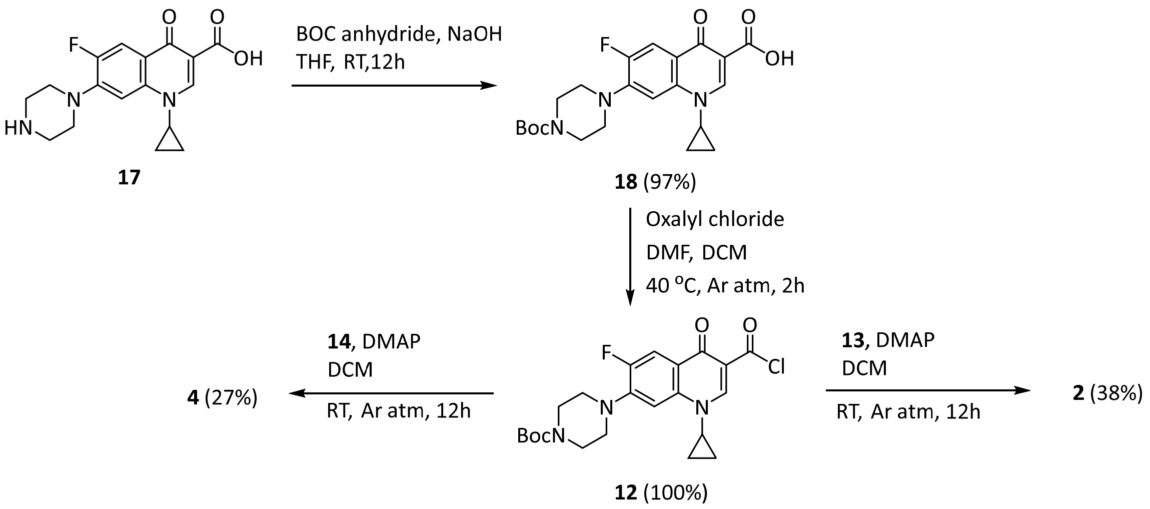


**Figure S2.** Synthetic route of compounds **2** and **4**, photoreleasable derivatives of Boc-ciprofloxacin.

**(5,5-difluoro-1,3,7,9-tetramethyl-5*H*-4l4,5l4-dipyrrolo[1,2-*c*:2',1'-*f*][1,3,2]diazaborinin-10-yl) methanol (13):**

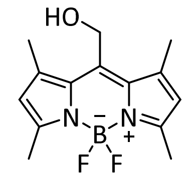

Compound prepared according to a previously described procedure (75% yield).[1]
$^{1}$H-NMR (300 MHz, $CDCl_3$): δ 6.09 (s, 2H), 4.91 (s, 2H), 2.53 (s, 6H), 2.51 (s, 6H).
$^{1}$H-NMR spectrum in agreement with published data.[1]

**(3,7-bis(4-(dimethylamino)styryl)-5,5-difluoro-1,9-dimethyl-5*H*-4l4,5l4- dipyrrolo[1,2-*c*:2', 1'-*f*][1,3,2]diazaborinin-10-yl)methanol (14):**

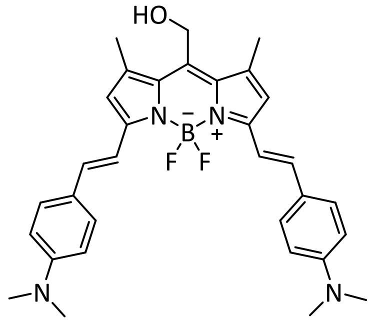

Compound prepared according to a previously described procedure (92% yield).[2]
$^{1}$H-NMR (300 MHz, DMSO-$d_6$): δ 7.48-7.43 (m, 6H), 7.27 (d, $J$ = 16 Hz, 2H), 6.94 (s, 2H), 6.79 (d, $J$ = 8.9 Hz, 4H), 5.45 (t, $J$ = 4.9 Hz, 1H), 4.74 (d, $J$ = 4.8 Hz, 2H), 3.00 (s, 12H), 2.54 (s, 6H).
$^{1}$H-NMR spectrum in agreement with published data.[2]

**1-ethyl-7-methyl-4-oxo-1,4-dihydro-1,8-naphthyridine-3-carbonyl chloride (16):**

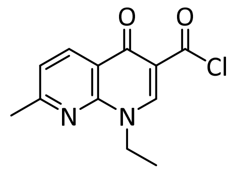

To a solution of nalidixic acid (0.43 mmol, 0.1 g) in dry DCM (4 ml) under argon atmosphere was added oxalyl chloride (1.29 mmol, 0.11 ml) and one drop of DMF. The resulting mixture was stirred for 2 hours at 40 °C. After that time, the solvent was evaporated *in vacuo* to give 0.11 g of a dark blue solid that was immediately used in the next step.

**(5,5-difluoro-1,3,7,9-tetramethyl-5*H*-4l4,5l4-dipyrrolo[1,2-*c*:2',1'-*f*][1,3,2]diazaborinin-10-yl) methyl 1-ethyl-7-methyl-4-oxo-1,4-dihydro-1,8-naphthyridine-3-carboxylate (1):**

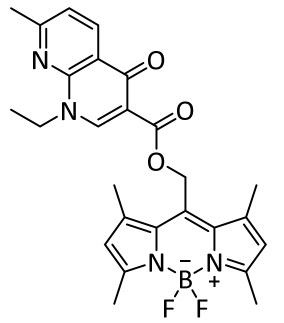


Compound **13** (80 mg, 0.29 mmol) was dissolved in dry DCM (4 ml) under argon atmosphere and 4-(dimethylamino)pyridine (70 mg, 0.58 mmol) was added. This mixture was added over a solution containing compound **16** (111 mg, 0.43 mmol) in dry DCM (3 ml) under argon atmosphere. The reaction mixture was stirred in the dark for 12 hours at room temperature. After that time, the solvent was evaporated *in vacuo*, and the resulting residue was purified by column chromatography (DCM:EtOAc 5:2) to obtain 61 mg (43%) of a red solid.

$^1$H-NMR (400 MHz, $CDCl_3$): δ 8.63 (d, *J* = 8.1 Hz, 1H), 8.55 (s, 1H), 7.24 (d, *J* = 8.1 Hz, 1H), 6.08 (s, 2H), 5.51 (s, 2H), 4.46 (q, *J* = 7.1 Hz, 2H), 2.65 (s, 3H), 2.54 (s, 6H), 2.44 (s, 6H), 1.47 (t, *J* = 7.1 Hz, 3H).

$^{13}$C-NMR (100 MHz, $CDCl_3$): δ 174.8, 164.2, 163.0, 156.6, 148.7, 142.1, 137.0, 134.0, 133.1, 122.4, 121.6, 121.5, 110.6, 58.1, 47.0, 25.2, 15.9, 15.4, 14.8.

HR-MS (ESI, [M+H]+): Calcd. for $C_{26}H_{27}BF_2N_4O_3$ + H: 493.2217; Found: 493.2226

**(3,7-bis(4-(dimethylamino)styryl)-5,5-difluoro-1,9-dimethyl-5*H*-4l4,5l4- dipyrrolo[1,2-*c*:2',1'-*f*] [1,3,2]diazaborinin-10-yl)methyl 1-ethyl-7-methyl-4-oxo- 1,4-dihydro-1,8-naphthyridine-3-carboxylate (3):**

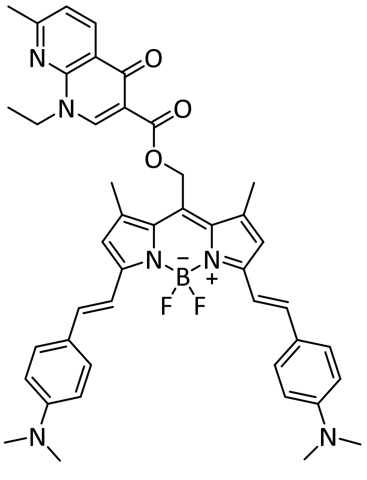


Compound **14** (83 mg, 0.15 mmol) was dissolved in dry DCM (4 ml) under argon atmosphere and 4-(dimethylamino)pyridine (37 mg, 0.30 mmol) was added. This mixture was added over a solution containing compound **16** (56 mg, 0.23 mmol) in dry DCM (3 ml) under argon atmosphere. The reaction mixture was stirred in the dark for 12 hours at room temperature. After that time, the solvent was evaporated *in vacuo*, and the resulting residue was purified by column chromatography (DCM:EtOAc:Toluene 2:3:1) to obtain 34 mg (30%) of a dark green solid.

$^{1}$H-NMR (300 MHz, $CDCl_3$): δ 8.64 (d, *J* = 8.1 Hz, 1H), 8.57 (s, 1H), 7.53 (d, *J* = 8.9 Hz, 6H), 7.25-7.15 (m, 3H), 6.71 (d, *J* = 8.9 Hz, 6H), 5.53 (s, 2H), 4.45 (q, *J* = 7.1 Hz, 2H), 3.04 (s, 12H), 2.64 (s, 3H), 2.46 (s, 6H), 1.45 (t, *J* = 7.1 Hz, 3H).
$^{13}$C-NMR (75 MHz, CDCl3): δ 175.0, 163.7, 162.9, 153.4, 151.1, 148.7, 148.6, 139.5, 137.2, 137.0, 129.4, 129.3, 125.2, 121.5, 121.4, 118.4, 115.0, 112.2, 110.6, 58.4, 46.9, 40.4, 25.2, 16.1, 15.4.
HR-MS (ESI, [M+Na]+): Calcd. for $C_{44}H_{45}BF_2N_6O_3$ +Na: 777.3514; Found: 777.3497

**7-(4-(*tert*-butoxycarbonyl)piperazin-1-yl)-1-cyclopropyl-6-fluoro-4-oxo-1,4- dihydroquinoline-3-carboxylic acid (18):**

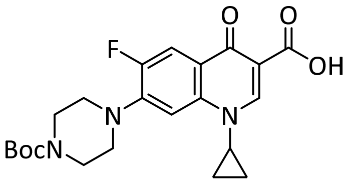


Ciprofloxacin (2 g, 6.0 mmol) and di-*tert*-butyl decarbonate (1.4 g, 6.6 mmol) were dissolved in THF (60 ml). Next, a solution of sodium hydroxide (0.48 g, 12.0 mmol) in water (12 ml) was added, and the mixture was stirred overnight at room temperature. After that time, the solvent was removed *in vacuo*, and the residue was taken into aqueous saturated ammonium chloride (200 ml). The aqueous phase was extracted with DCM (200 ml) three times. The organic layer was washed with brine (400 ml) and dried over anhydrous $MgSO_4$. The solvent was evaporated *in vacuo* to obtain 2.51 g (97%) of white solid.
$^{1}$H-NMR (300 MHz, $CDCl_3$): δ 14.94 (s, 1H), 8.71 (s, 1H), 7.96 (d, *J* = 12.9 Hz, 1H), 7.35 (d, *J* = 7.1 Hz, 1H), 3.72 – 3.61 (m, 4H), 3.59-3.50 (m, 1H), 3.34 – 3.21 (m, 4H), 1.49 (s, 9H), 1.41- 1.35 (m, 2H), 1.26 – 1.15 (m, 2H).

***Tert*-butyl 4-(3-(chlorocarbonyl)-1-cyclopropyl-6-fluoro-4-oxo-1,4- dihydroquinolin-7-yl) piperazine-1-carboxylate (12):**

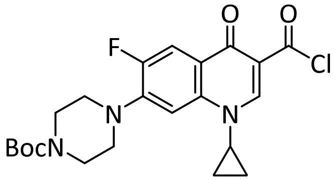


To a solution of compound **18** (0.15 g, 0.35 mmol) in dry DCM (5 ml) under argon atmosphere, oxalyl chloride (88 µl, 1.05 mmol) and one drop of DMF were added. The reaction was stirred for 2 hours at 40 °C. After that time, the solvent was removed *in vacuo* to give 0.16 g (100%) of an orange solid that was immediately used in the next step.

**(5,5-difluoro-1,3,7,9-tetramethyl-5*H*-4l4,5l4-dipyrrolo[1,2-*c*:2',1'-*f*][1,3,2] diazaborinin-10-yl) methyl 7-(4-(*tert*-butoxycarbonyl)piperazin-1-yl)-1 cyclopropyl-6-fluoro-4-oxo-1,4-dihydroquinoline-3-carboxylate (2):**

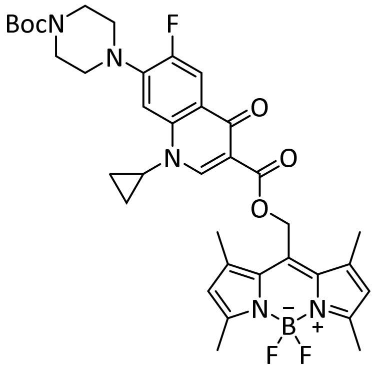

Compound **13** (67 mg, 0.24 mmol) was dissolved in dry DCM (4 ml) under argon atmosphere and 4-(dimethylamino)pyridine (57 mg, 0.47 mmol) was added. This mixture was added over a solution containing compound **12** (160 mg, 0.36 mmol) in dry DCM (3 ml) under argon atmosphere. The reaction mixture was stirred in the dark for 12 hours at room temperature. After that time, the solvent was evaporated *in vacuo*, and the resulting residue was purified by column chromatography (DCM:EtOAc 5:2) to obtain 63 mg (38%) of a red solid.

$^{1}$H-NMR (300 MHz, $CDCl_3$): δ 8.45 (s, 1H), 8.05 (d, *J* = 13.1 Hz, 1H), 7.24 (d, 1H), 6.08 (s, 2H), 5.51 (s, 2H), 3.68 – 3.60 (m, 4H), 3.45 – 3.33 (m, 1H), 3.24 – 3.16 (m, 4H), 2.53 (s, 6H), 2.45 (s, 6H), 1.49 (s, 9H), 1.34 – 1.26 (m, 2H), 1.12 – 1.02 (m, 2H).

$^{13}$C-NMR (75 MHz, $CDCl_3$): δ 173.1, 164.6, 156.5, 155.2, 154.7, 151.9, 148.2, 144.8, 142.1, 138.1, 134.1, 133.1, 122.4, 113.8, 109.3, 105.1, 80.4, 58.1, 50.1, 43.6, 34.7, 28.6, 15.9, 14.8, 8.5.

HR-MS (ESI, [M+H]+): Calcd. for $C_{36}H_{41}BF_3N_5O_5$ +H: 692.3225; Found: 692.3235

**(3,7-bis(4-(dimethylamino)styryl)-5,5-difluoro-1,9-dimethyl-5*H*-4l4,5l4- dipyrrolo[1,2-*c*:2',1'-*f*] [1,3,2]diazaborinin-10-yl)methyl 7-(4-(*tert*-butoxycarbonyl)piperazin-1-yl)-1-cyclopropyl-6-fluoro -4-oxo-1,4- dihydroquinoline-3-carboxylate (4):**

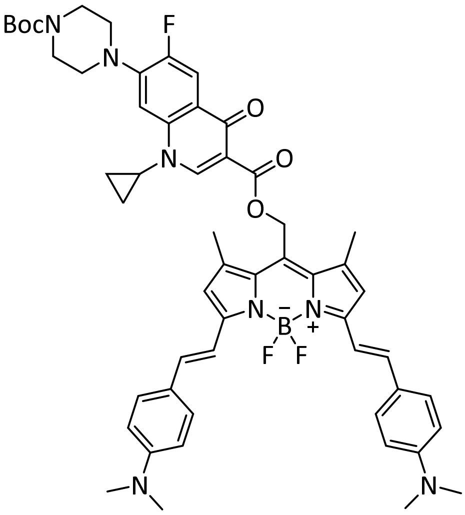

Compound **14** (83 mg, 0.15 mmol) was dissolved in dry DCM (4 ml) under argon atmosphere and 4-(dimethylamino)pyridine (37 mg, 0.30 mmol) was added. This mixture was added over a solution

containing compound **12** (103 mg, 0.23 mmol) in dry DCM (3 ml) under argon atmosphere. The reaction mixture was stirred in the dark for 12 hours at room temperature. After that time, the solvent was evaporated *in vacuo*, and the resulting residue was purified by column chromatography (DCM:EtOAc:Toluene 2:3:1) to obtain 34 mg (30%) of a dark green solid.

$^{1}$H-NMR (300 MHz, $CDCl_3$): δ 8.45 (s, 1H), 8.07 (d, *J* = 13.1 Hz, 1H), 7.53 (d, *J* = 8.9 Hz, 6H), 7.25 – 7.17 (m, 3H), 6.71 (d, *J* = 8.7 Hz, 6H), 5.54 (s, 2H), 3.66 – 3.59 (m, 4H), 3.42 – 3.32 (m, 1H), 3.22 – 3.16 (m, 4H), 3.03 (s, 12H), 2.48 (s, 6H), 1.49 (s, 9H), 1.26 – 1.21 (m, 2H), 1.09 – 1.02 (m, 2H).

$^{13}$C-NMR (75 MHz, $CDCl_3$): δ 173.3, 164.1, 154.8, 153.4, 151.1, 148.0, 144.7, 144.6, 139.6, 138.1, 137.1, 134.8, 129.4, 129.3, 127.9, 125.2, 123.3, 118.4, 115.0, 113.9, 113.6, 112.2, 109.4, 105.2, 80.3, 58.4, 50.1, 43.6, 40.4, 34.7, 28.6, 16.1, 8.6.

HR-MS (ESI, [M+H]+): Calcd. for $C_{54}H_{59}BF_3N_7O_5$ +H: 954.4704; Found: 954.4659

**6-(2-(2-(2-methoxyethoxy)ethoxy)ethoxy)-3,4-dihydronaphthalen-1(2*H*)-one (7):**

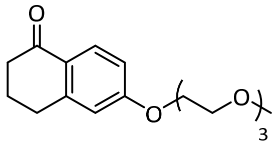

To a solution of 6-hydroxy-1-tetralone **6** (1 g, 6.2 mmol) in anhydrous DMF (15 ml) under argon atmosphere was added sodium carbonate (1.3 g, 12.3 mmol), 1-chloro-2-[2-(2-methoxyethoxy)ethoxy]ethane (1.2 g, 6.7 mmol), and sodium iodide as a catalyst. The resulting mixture was stirred for 16 hours at 100 °C. After that time, the solvent was removed *in vacuo*, and the resulting product was neutralized with 1M HCl. Then, it was extracted with DCM (60 ml) twice, and the organic layers were washed with brine (60 ml). Next, the organic layer was dried over anhydrous $MgSO_4$, and the solvent was evaporated *in vacuo* to give 1.8 g (95%) of a clear oil.

$^{1}$H-NMR (400 MHz, $CDCl_3$): δ 7.99 (d, *J* = 8.7 Hz, 1H), 6.83 (dd, *J* = 8.8, 2.5 Hz, 1H), 6.71 (d, *J* = 2.5 Hz, 1H), 4.20 – 4.15 (m, 2H), 3.88-3.84 (m, 2H), 3.75-3.72 (m, 2H), 3.70 – 3.61 (m, 4H), 3.56-3.53 (m, 2H), 3.37 (s, 3H), 2.92-2.89 (m, 2H), 2.62-2.58 (m, 2H), 2.10 (quint, *J* = 6.4 Hz, 2H).

$^{13}$C-NMR (100 MHz, $CDCl_3$): δ 197.3, 162.9, 147.0, 129.7, 126.6, 113.6, 113.4, 72.1, 71.0, 70.8, 70.7, 69.7, 67.7, 59.2, 39.0, 30.3, 23.5.

HR-MS (ESI, [M+H]+): Calcd. for $C_{17}H_{24}O_5$ +H: 309.1697; Found: 309.1701

**6-(2-(2-(2-methoxyethoxy)ethoxy)ethoxy)-3,4-dihydronaphthalen-1(2*H*)-one oxime (8):**

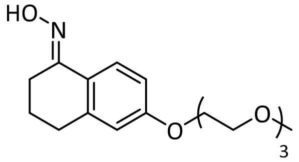

Sodium acetate (0,29 g, 3.6 mmol) was dissolved in water (3 ml) and added over a solution of hydroxylamine hydrochloride (0.25 g, 3.6 mmol) in water (3 ml). The resulting mixture was added over a solution of compound **7** (1 g, 3.2 mmol) in methanol (10 ml) and heated at 35 °C. Next, the sample was stirred for 2 hours at 80 °C. After that time, the reaction was cooled at room temperature, and the solvent was removed *in vacuo*. Next, water (30 ml) was added to the residue, and the aqueous phase was extracted with DCM (50 ml) twice. The combined organic layers were washed with brine (50 ml) and dried over anhydrous $MgSO_4$. Then, the solvent was evaporated *in vacuo* to result in 0.99 g (94%) of a clear oil.

$^{1}$H-NMR (400 MHz, $CDCl_3$): δ 7.77 (d, *J* = 8.7 Hz, 1H), 6.76 (dd, *J* = 8.8, 2.7 Hz, 1H), 6.66 (d, *J* = 2.6 Hz, 1H), 4.16 – 4.09 (m, 2H), 3.87 – 3.82 (m, 2H), 3.75 – 3.71 (m, 2H), 3.70 – 3.62 (m, 4H), 3.58 – 3.52 (m, 2H), 3.37 (s, 3H), 2.79 (t, *J* = 6.6 Hz, 2H), 2.70 (t, *J* = 6.1 Hz, 2H), 1.85 (quint, *J* = 6.5 Hz, 2H).
$^{13}$C-NMR (100 MHz, $CDCl_3$): δ 159.6, 155.2, 141.6, 125.7, 123.5, 113.8, 113.6, 72.0, 70.9, 70.8, 70.7, 69.8, 67.4, 59.1, 30.2, 23.9, 21.5.
HR-MS (ESI, [M+H]+): Calcd. for $C_{17}H_{25}NO_5$ +H: 324.1805; Found: 324.1811

**7-(2-(2-(2-methoxyethoxy)ethoxy)ethoxy)-4,5-dihydro-1*H*-benzo[g]indole (9):**

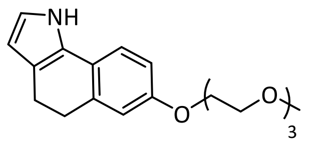


In a three-neck flask, under argon atmosphere, compound **8** (1 g, 3.1 mmol) was dissolved in DMSO (25 ml). Next, lithium hydroxide monohydrate (0.7 g, 16.7 mmol) was added, and a light flow of acetylene was passed through the reaction mixture. The mixture was heated at 135 °C while keeping the acetylene flow for 6 hours. After that time, the reaction mixture was allowed to reach room temperature, and distilled water (60 ml) was added. Then, the aqueous phase was extracted with diethyl ether (100 ml) five times. The combined organic layers were extracted with water (100 ml) once. Finally, the organic layer was dried over anhydrous $MgSO_4$ and the solvent was removed *in vacuo*. The obtained residue was purified by column chromatography (DCM:EtOAc 5:1)to give 0.31 g (30%) of a clear oil.
$^{1}$H-NMR (400 MHz, $CDCl_3$): δ 8.34 (s, 1H), 7.06 (d, *J* = 8.3 Hz, 1H), 6.80 (d, *J* = 2.5 Hz, 1H), 6.72 (d, *J* = 2.7 Hz, 1H), 6.71 (t, *J* = 2.7 Hz, 1H), 6.10 (t, *J* = 2.5 Hz, 1H), 4.14 – 4.07 (m, 2H), 3.86 – 3.82 (m, 2H), 3.76 – 3.72 (m, 2H), 3.71-3.64 (m, 4H), 3.59 – 3.52 (m, 2H), 3.38 (s, 3H), 2.89 (t, *J* = 7.5 Hz, 2H), 2.72 (t, *J* = 7.3 Hz, 2H).
$^{13}$C-NMR (100 MHz, $CDCl_3$): δ 156.6, 136.8, 127.8, 123.1, 119.3, 118.5, 117.4, 115.6, 112.1, 108.0, 72.1, 70.9, 70.8, 70.7, 69.9, 67.6, 59.2, 30.6, 21.9.
HR-MS (ESI, [M+H]+): Calcd. for $C_{19}H_{25}NO_4$ +H: 332.1856; Found: 332.1855

**8-((acetyloxy)methyl)-17,17-difluoro-3,13-bis(2-(2-(2-methoxyethoxy)ethoxy)ethoxy)-5,10,11,17 -tetrahydro-6H benzo[g]benzo[6',7']indolo[1',2':3,4] [1,3,2] diazaborinino[1,6-a]indol-16-ium (10):**

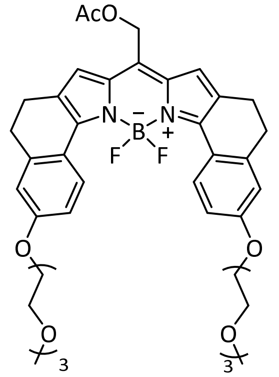


To a solution of compound **9** (0.94 mmol, 0.31 g) in dry DCM (10 ml) under argon atmosphere, was added acetoxyacetyl chloride (0.56 mmol, 60 µl) and the mixture was stirred in the dark at 40 °C for 2 hours. Then, TEA (1.7 mmol, 0.23 ml) was added. The resulting mixture was allowed to stir for 15

minutes. Next, boron trifluoride diethyl etherate (1.7 mmol, 0.21 ml) was added. After 1 hour of stirring, another portion of TEA (1.7 mmol, 0.23 ml) and boron trifluoride diethyl etherate (1.7 mmol, 0.21 ml) were added, and the stirring continued for one more hour. After that time, the solvent was evaporated *in vacuo*, and the obtained residue was purified by column chromatography (DCM:MeOH 32:1) to give 85 mg (23%) of a deep blue-green solid.
$^1$H-NMR (400 MHz, $CDCl_3$): δ 8.68 (d, *J* = 9.0 Hz, 2H), 6.98 (s, 2H), 6.95 (dd, *J* = 8.9, 2.7 Hz, 2H), 6.82 (d, *J* = 2.7 Hz, 2H), 5.25 (s, 2H), 4.24 – 4.19 (m, 4H), 3.91 – 3.86 (m, 4H), 3.78 – 3.73 (m, 4H), 3.72 – 3.65 (m, 8H), 3.58 – 3.53 (m, 4H), 3.38 (s, 6H), 2.88 (t, *J* = 7.0 Hz, 4H), 2.73 (t, *J* = 6.4 Hz, 4H), 2.10 (s, 3H).
$^{13}$C-NMR (100 MHz, $CDCl_3$): δ 170.7, 160.2, 152.6, 143.1, 136.2, 132.6, 130.4, 128.3, 122.1, 121.6, 115.1, 113.1, 72.1, 71.0, 70.8, 70.7, 69.7, 67.5, 59.9, 59.2, 31.0, 22.5, 21.1.
HR-MS (ESI, [M+H]+): Calcd. for $C_{42}H_{51}BF_2N_2O_{10}$ +H: 790.3568; Found: 790.3562

**17,17-difluoro-8-(hydroxymethyl)-3,13-bis(2-(2-(2-methoxyethoxy)ethoxy) ethoxy)-5,10,11,17-tetrahydro-6H-benzo[g]benzo[6',7']indolo[1',2':3,4] [1,3,2]diazaborinino [1,6-a]indol-16-ium (11):**

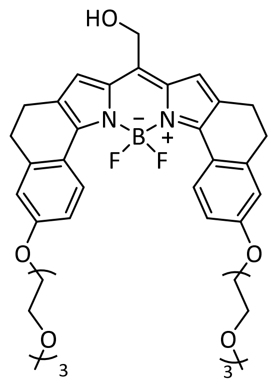

To a solution of compound **10** (0.11 mmol, 85 mg) in a mixture of DCM (25 ml) and MeOH (10 ml) was added a solution of potassium carbonate (0.39 mmol, 53 mg) in water (1 ml). The reaction mixture was stirred in the dark at room temperature for 3 hours. After that time, the organic solvent was removed *in vacuo*, and the residue was extracted with DCM (40 ml) twice. Next, the combined organic layers were washed with 1M HCl (10 ml) and brine (20 ml). The organic layer was dried over anhydrous $MgSO_4$, and the solvent was removed *in vacuo*. The resulting residue was purified by column chromatography (DCM:MeOH 40:1) to obtain 48 mg (58%) of a dark green solid.
$^1$H-NMR (400 MHz, $CDCl_3$): δ 8.68 (d, *J* = 9.0 Hz, 2H), 7.04 (s, 2H), 6.96 (dd, *J* = 8.9, 2.7 Hz, 2H), 6.82 (d, *J* = 2.7 Hz, 2H), 4.81 (s, 2H), 4.28 – 4.14 (m, 4H), 3.93 – 3.84 (m, 4H), 3.80 – 3.72 (m, 4H), 3.72 – 3.64 (m, 8H), 3.59 – 3.52 (m, 4H), 3.38 (s, 6H), 2.88 (t, *J* = 7.0 Hz, 4H), 2.73 (t, *J* = 7.0 Hz, 4H).
$^{13}$C-NMR (100 MHz, $CDCl_3$): δ 160.1, 152.4, 143.0, 135.5, 133.5, 132.4, 121.8, 121.8, 121.7, 115.1, 113.1, 72.1, 71.0, 70.8, 70.7, 69.8, 67.6, 59.9, 59.2, 31.0, 22.6.
HR-MS (ESI, [M+H]+): Calcd. for $C_{40}H_{49}BF_2N_2O_9$ +H: 790.3568; Found: 790.3562

**8-((((7-(4-(*tert*-butoxycarbonyl)piperazin-1-yl)-1-cyclopropyl-6-fluoro-4-oxo- 1,4-dihydroquinolin-3-yl)carbonyl)oxy)methyl)-17,17-difluoro-3,13-bis(2-(2-(2- methoxyethoxy)ethoxy)ethoxy)-5,10, 11,17-tetrahydro-6H benzo[g]benzo [6',7'] indolo [1',2':3,4][1,3,2]diazaborinino[1,6-a]indol-16-ium (5):**

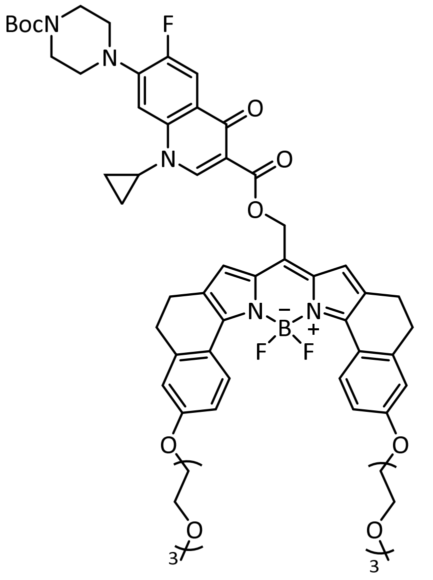


Compound **11** (48 mg, 0.06 mmol) was dissolved in dry DCM (3 ml) under argon atmosphere and 4-(dimethylamino)pyridine (16 mg, 0.13 mmol) was added. This mixture was added over a solution containing compound **12** (39 mg, 0.09 mmol) in dry DCM (2 ml) under argon atmosphere. The reaction mixture was stirred in the dark for 12 hours at room temperature. After that time, the solvent was evaporated *in vacuo*, and the resulting residue was purified by column chromatography (DCM:MeOH 40:1) to obtain 21 mg (28%) of a dark green solid.

$^{1}$H-NMR (400 MHz, $CDCl_3$): δ 8.68 (d, *J* = 9.0 Hz, 2H), 8.46 (s, 1H), 8.07 (d, *J* = 13.1 Hz, 1H), 7.24 (d, *J* = 7.0 Hz, 1H), 7.18 (s, 2H), 6.95 (dd, *J* = 8.9, 2.7 Hz, 2H), 6.82 (d, *J* = 2.7 Hz, 2H), 5.49 (s, 2H), 4.25 – 4.16 (m, 4H), 3.91 – 3.81 (m, 4H), 3.79 – 3.73 (m, 4H), 3.72 – 3.61 (m, 12H), 3.59 – 3.52 (m, 4H), 3.38 (s, 6H), 3.45-3.33 (m, 1H), 3.20 (m, 4H), 2.88 (t, *J* = 6.2 Hz, 4H), 2.74 (t, *J* = 7.1 Hz, 4H), 1.49 (s, 9H), 1.31 – 1.26 (m, 2H), 1.10-1.05 (m, 2H).

$^{13}$C-NMR (100 MHz, $CDCl_3$): δ 173.2, 164.3, 160.1, 154.8, 154.7, 152.4, 152.3, 148.5, 144.6, 143.1, 138.1, 136.2, 132.6, 130.3, 129.3, 122.6, 121.7, 115.1, 113.8, 113.6, 113.1, 109.5, 105.3, 80.4, 72.1, 71.0, 70.8, 70.7, 69.8, 67.5, 60.1, 59.2, 53.6, 50.1, 34.8, 31.0, 29.8, 28.5, 22.6, 8.4.

HR-MS (ESI, [M+H]+): Calcd. for $C_{62}H_{73}BF_3N_5O_{13}$ +H: 1163.5359; Found: 1163.5340

## 3. HPLC Conditions

A Phenomenex C18 column (250 mm x 4.6 mm x 5 µm) was employed as the stationary phase. The mobile phase consisted of different mixtures of acetonitrile and water containing 0.1% of trifluoroacetic acid. The flow in all cases was 1 ml/minute, and the injection volume was 20 µl, previously filtered through a 0.45 µm PTFE membrane filter. The gradient methods used for each compound are described next.

**Table S1.** HPLC method A, used for the analysis of compounds **1** and **2**.

| Time (min) | Mobile phase composition (%) | |
|---|---|---|
| | Acetonitrile | Water (0.1% TFA) |
| 0 | 80 | 20 |
| 5 | 40 | 60 |
| 7 | 40 | 60 |
| 14 | 10 | 90 |
| 15 | 10 | 90 |

For compounds **1** and **2**, the detection was carried out in dual-mode at 254 or 282, and 520 nm to monitor the release of the antibiotic as well as the remaining protected compound. Retention times are indicated next: $t_{R1}$ = 12.8 min, $t_{R2}$ = 14.7 min, $t_{R\textbf{Nalidixic acid}}$ = 8.0 min, $t_{R\textbf{Boc-ciprofloxacin}}$ = 11.3 min.

**Table S2.** HPLC method B, used for the analysis of compounds **3**, **4**, and **5**.

| Time (min) | Mobile phase composition (%) | |
|---|---|---|
| | Acetonitrile | Water (0.1% TFA) |
| 0 | 80 | 20 |
| 5 | 40 | 60 |
| 7 | 40 | 60 |
| 14 | 10 | 90 |
| 20 | 10 | 90 |

For compounds **3** and **4**, the detection was carried out in dual-mode at 254 or 282, and 730 nm in order to monitor the release of the antibiotic and the remaining protected compound. For compound **5**, the selected wavelengths in dual-mode were 282 and 675 nm. Retention times are indicated next: $t_{R\mathbf{3}}$ = 15.8 min, $t_{R\mathbf{4}}$= 16.8 min, $t_{R\mathbf{5}}$ = 15.9 min, $t_{R\textbf{Nalidixic acid}}$ = 8.0 min, $t_{R\textbf{Boc-ciprofloxacin}}$ = 11.3 min.

The following data correspond to the minimum purity of the samples as measured by HPLC:

- Compound 1: purity > 92%
- Compound 2: purity > 92%
- Compound 3: purity > 95%
- Compound 4: purity > 95%
- Compound 5: purity > 95%

## 4. Photorelease studies

All compounds (**1**-**5**) were irradiated at a concentration of 256 mg/L in mixtures of DMSO/$H_2O$ with the LED lamp indicated in table 3. The release process was followed by HPLC (**1**-**4**) employing method A or B as indicated in HPLC conditions section, or $^1$H-NMR (**5**).

**Table S3.** Irradiation conditions of photoreleasable quinolones

| Compound | DMSO/$H_2O$ | Volume (ml) | LED lamp |
|---|---|---|---|
| **1** | 60:40 | 1 | Green (λ = 520 nm) |
| **2** | 60:40 | 1 | Green (λ = 520 nm) |
| **3** | 80:20 | 1 | NIR (λ = 730 nm) |
| **4** | 80:20 | 1 | NIR (λ = 730 nm) |
| **5** | 60:40 | 1 | Red (λ = 625 nm) |

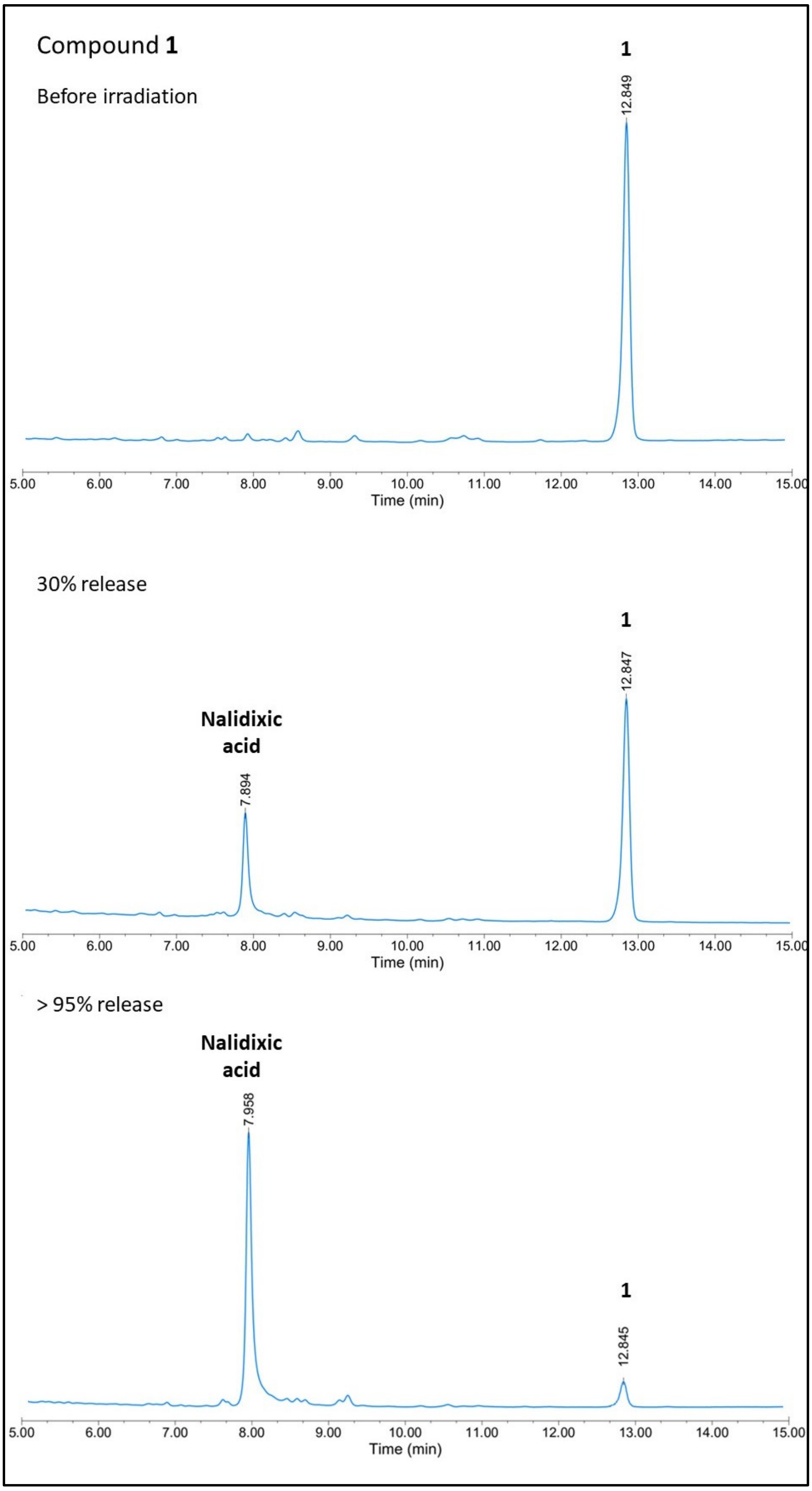


**Figure S3.** Release process of compound **1** upon irradiation with green light. The peak corresponding to compound **1** decreases while the peak of nalidixic acid grows. Release yield > 95%.

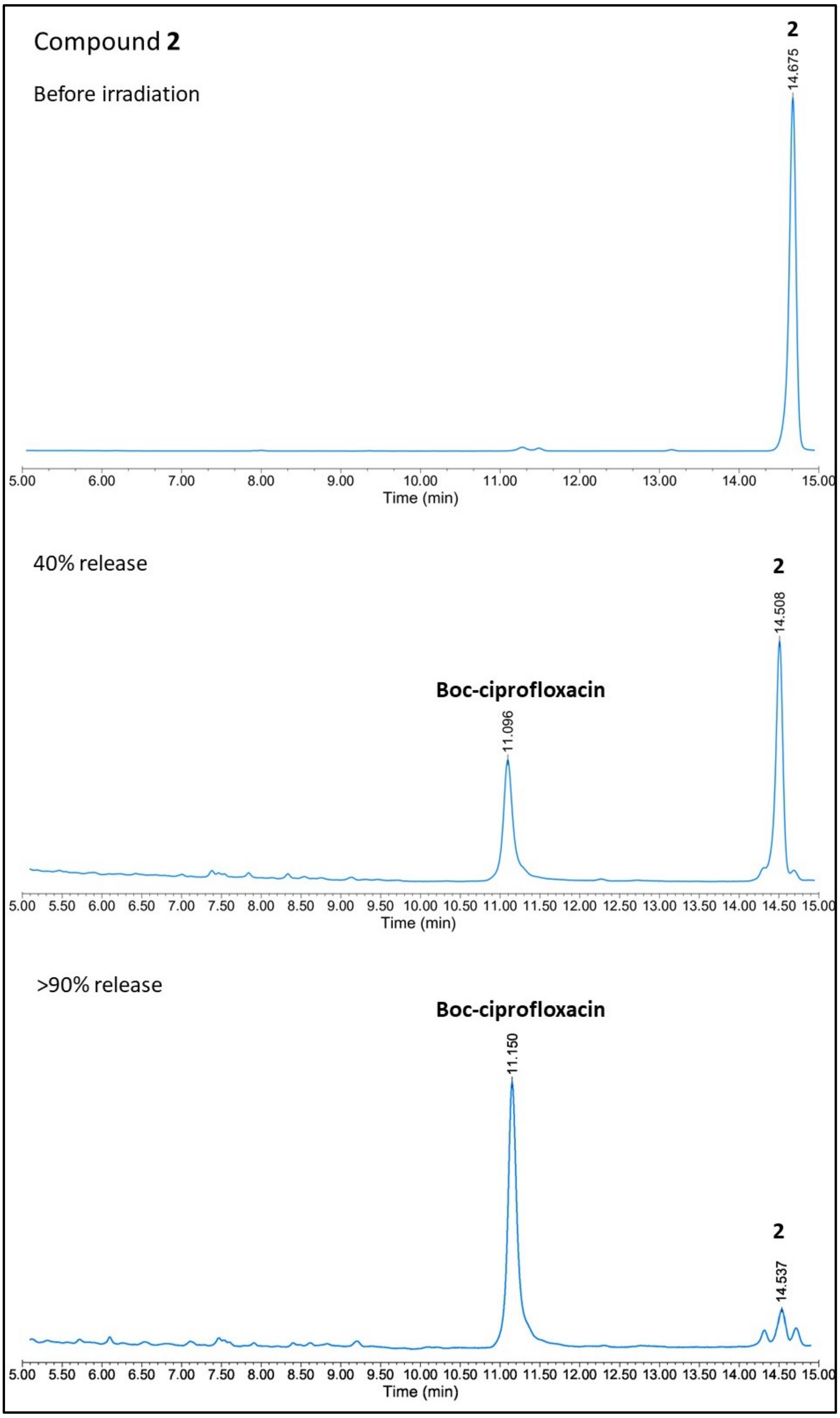


**Figure S4.** Release process of compound **2** upon irradiation with green light. The peak corresponding to compound **2** decreases while the peak of Boc-ciprofloxacin grows. Release yield > 90%.

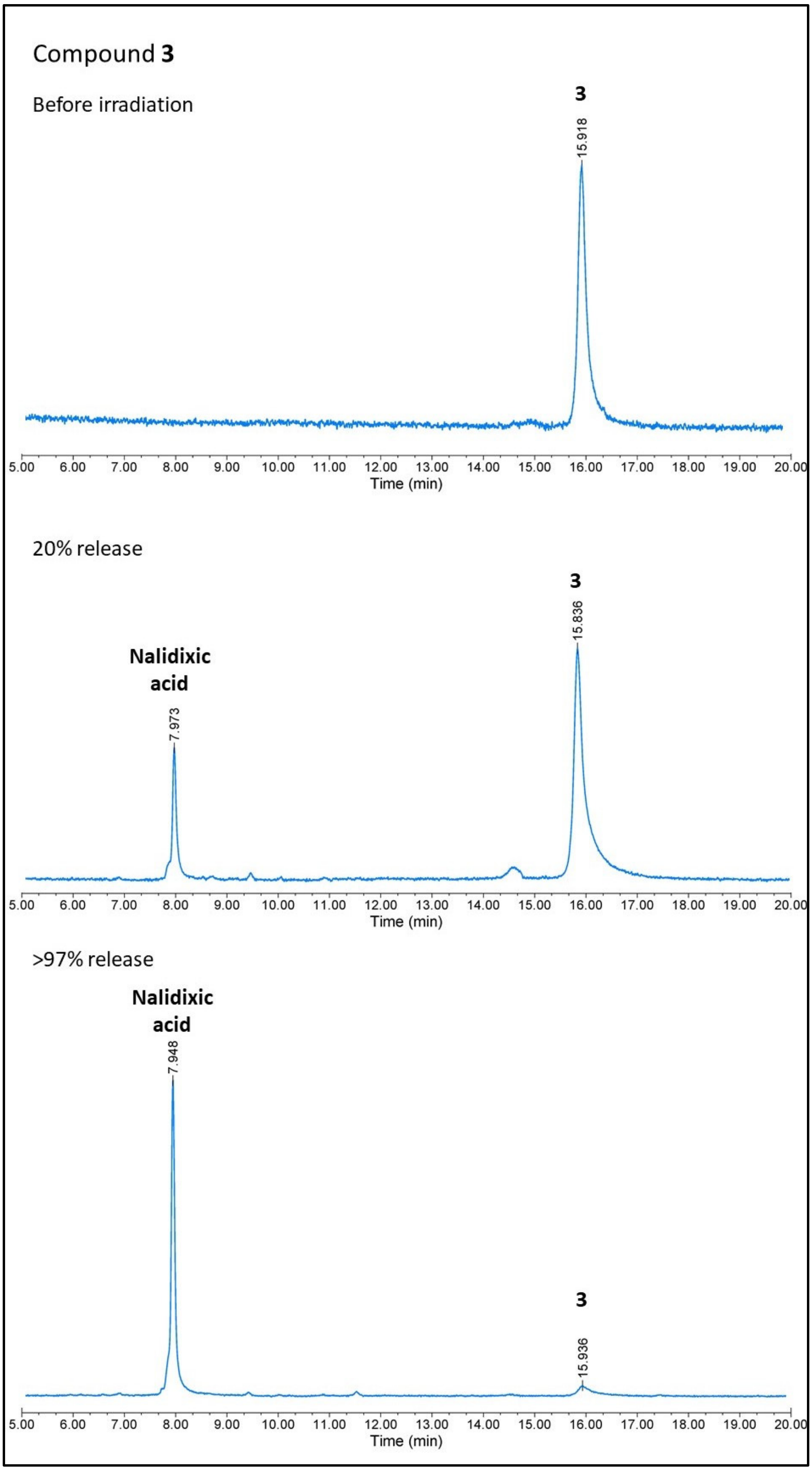


**Figure S5.** Release process of compound **3** upon irradiation with NIR light. The peak corresponding to compound **3** decreases while the peak of nalidixic acid grows. Release yield > 97%.

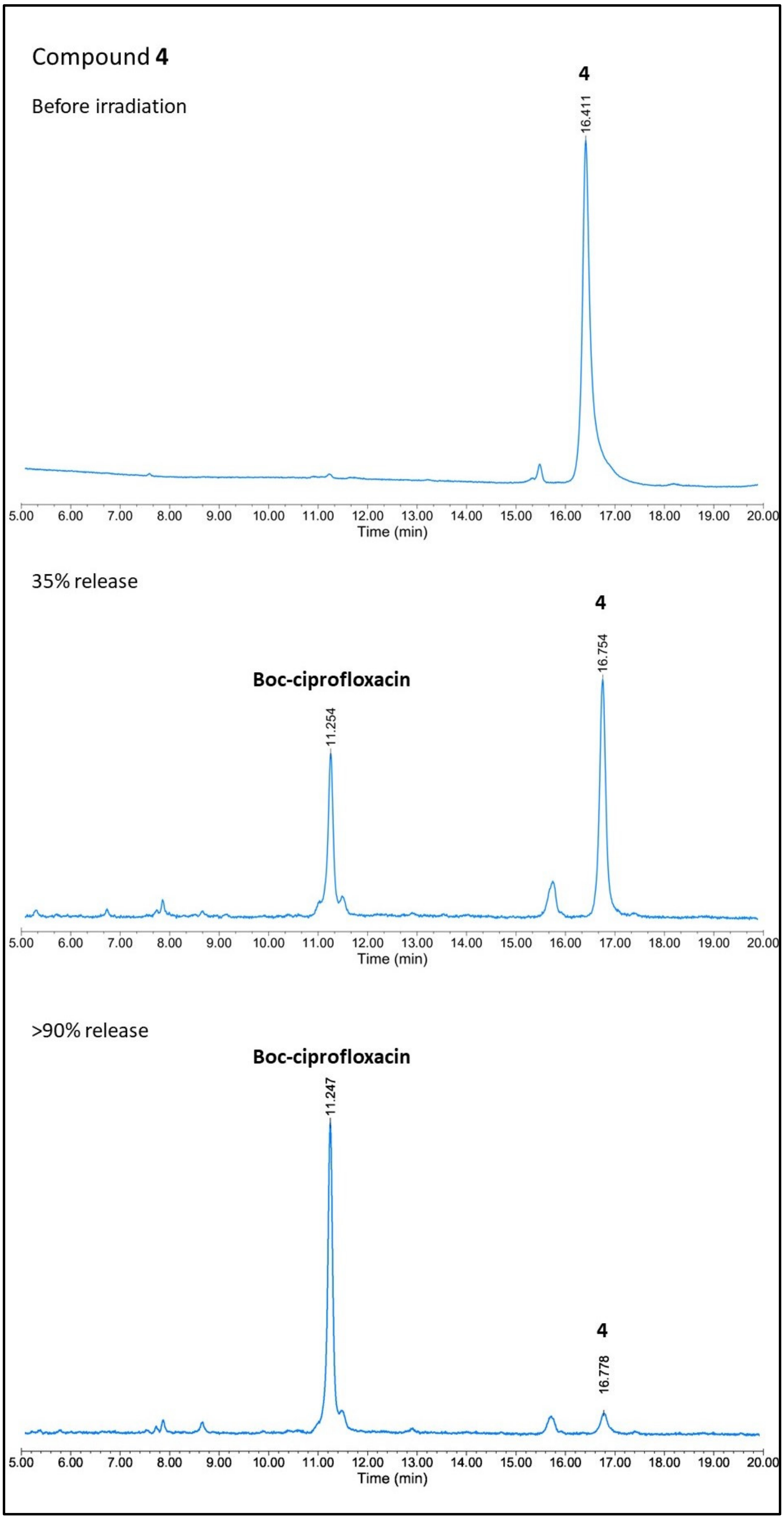


**Figure S6.** Release process of compound **4** upon irradiation with NIR light. The peak corresponding to compound **4** decreases while the peak of Boc-ciprofloxacin grows. Release yield > 90%.

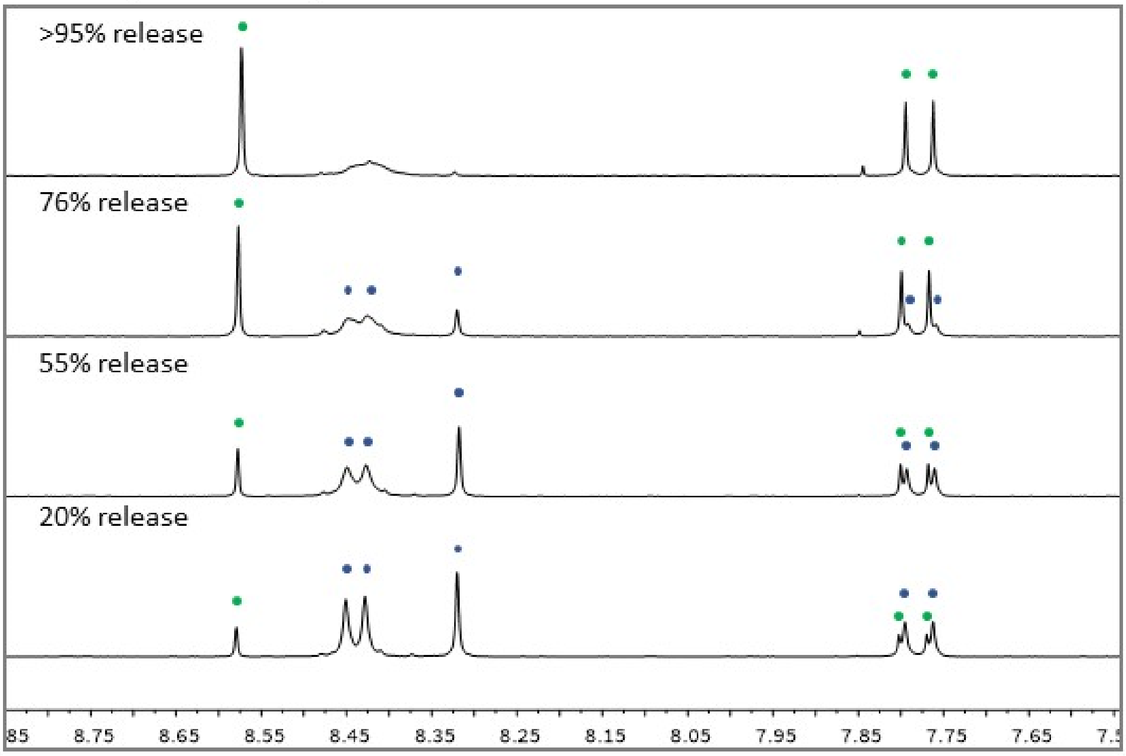


**Figure S7.** Release process of compound **5** upon irradiation with red light. The peaks corresponding to compound **5** (in blue) gradually disappear while the peaks of Boc-ciprofloxacin (in green) grow. Release yield > 95%.

## 5. Quantum yield measurements

The adequate light source was selected according to the sample under study. Compounds **1** and **2** were irradiated with green light using a 30W RGB LED. Compounds **3** and **4** were irradiated using a 515mW far-red LED. Compound **15** was irradiated with a 30W RGB LED in red light mode. The LED of choice was incorporated in the setup represented in Figure S8. Samples were prepared in a concentration close to 5 x $10^{-5}$ M and dissolved in different mixtures of DMSO and water according to their solubility (see Table S3). All samples were equipped with a magnetic stirrer to ensure homogeneous irradiation of the total volume. Prior to the irradiation process, the sample was analyzed by HPLC to check that the protected form of the antibiotic was being irradiated. The HPLC conditions for each compound can be found in the previous section.

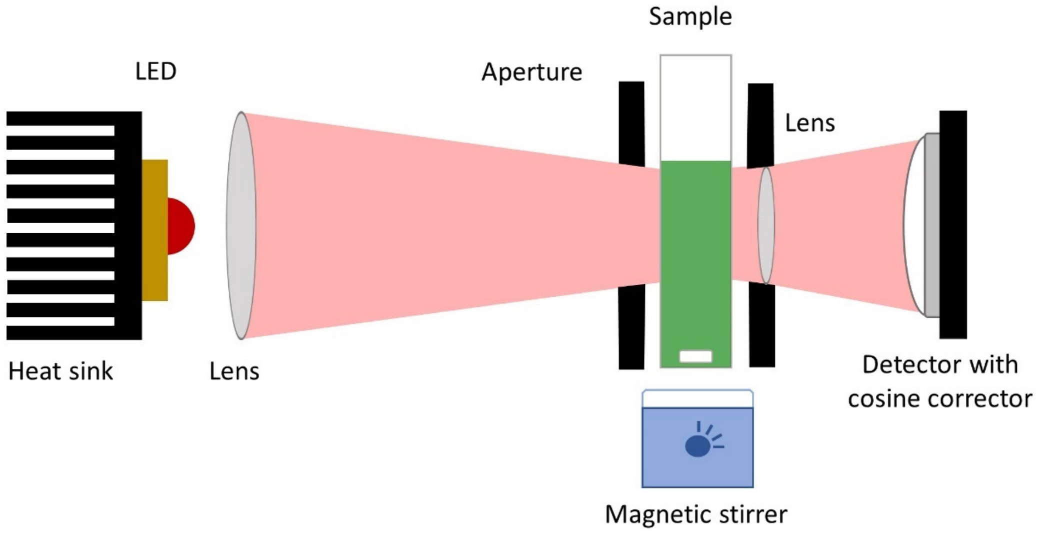


**Figure S8.** Schematic representation of the instrumental setup used to measure the quantum yield.[3] The light emitted by the LED is focused through a lens and imaged into the sample equipped with a magnetic stirrer. The radiation not absorbed by the sample is collected by a cosine corrector.

Once the LED was placed in the setup, the light was blocked with a laser dimming paper in front of the lens. This allowed the determination of the ambient light, which was later substrated from subsequent measurements. Next, the paper was removed, and a reference cuvette containing the solvent used to dissolve the samples was inserted. The light not absorbed by the sample was collected by a cosine corrector coupled to an Ocean Optics USB4000-UV-Vis detector. The transmitted radiant flux ($P_{ref}$), was obtained in moles of photons·$s^{-1}$·$m^{-2}$.
Then, the reference was exchanged by a cuvette containing the sample that was irradiated for a determined time. During that time, the light not absorbed by the sample ($P_{sample}$) was monitored by continuous acquisition of the transmitted radiant flux in moles of photons·$s^{-1}$·$m^{-2}$. Note that upon irradiation, the conversion from the protected antibiotic to the released form should be below 10% to avoid competitive absorption between the initial product and the byproduct of the photocage. After irradiation, the sample's conversion was quantified by HPLC, which allowed the calculation of the number of antibiotic molecules released.

The light absorbed by the sample can be calculated as the difference between the light not absorbed by the reference and the light not absorbed by the sample, as expressed in equation 1:

$$P_{abs} = P_{ref} - P_{sample}$$

**Equation 1.** Equation expressing the moles of photons·s$^{-1}$·m$^{-2}$ absorbed by the sample. $P_{ref}$ corresponds with the transmitted radiant flux measured for the reference with solvent, $P_{sample}$ refers to the transmitted radiant flux recorded when the sample was inserted in the setup.

To conclude the experiment, the number of photons absorbed by the sample, as well as the number of molecules of antibiotic released, were introduced in equation 1 to obtain the quantum yield.

## 6. Determination of minimal inhibitory concentration (MIC)

The minimal inhibitory concentration (MIC) was determined by the microdilution method in cation adjusted Mueller-Hinton broth (Becton Dickinson).[4] *Escherichia coli* ATCC 25922 strain was cultured in Brain-Heart Infusion (BHI) agar (Becton Dickinson) for 24h at 37 °C. Overnight colonies of a pure culture on agar were used to prepare an inoculum adjusted to the turbidity of a 0.5 McFarland in sterile saline solution (1 x $10^8$ CFU/ml). This suspension was diluted in cation adjusted Mueller-Hinton broth until a density of 5 x $10^5$ CFU/mL and exposed to a serial twofold dilution of each compound tested (0.03-32 mg/L) with a final volume of 0.1 mL in a 96 well microdilution tray. The MIC value was determined as the lowest concentration of compound where no visible growth was apparent. Positive (with bacteria and cation adjusted Mueller-Hinton broth) and negative (only with cation adjusted Mueller-Hinton broth) control wells were included in all assays. Moreover, control tests of solvents (mixtures of water and DMSO 40:60 or 20:80) were included to check the survival of the bacterium in these concentrations.

In the experiments in which the compounds were activated in presence of the bacteria, for the irradiation of the plates, we used a 100W RGB LED in green mode and a 50W RGB LED in red mode. The plates were placed on top of each LED and inside an incubator at a controlled temperature of 37 °C (Figure S9). The irradiation of the plates took place for an hour. After that time, the LEDS were switched off and the plates were kept in the incubator for a total time of 24 hours.

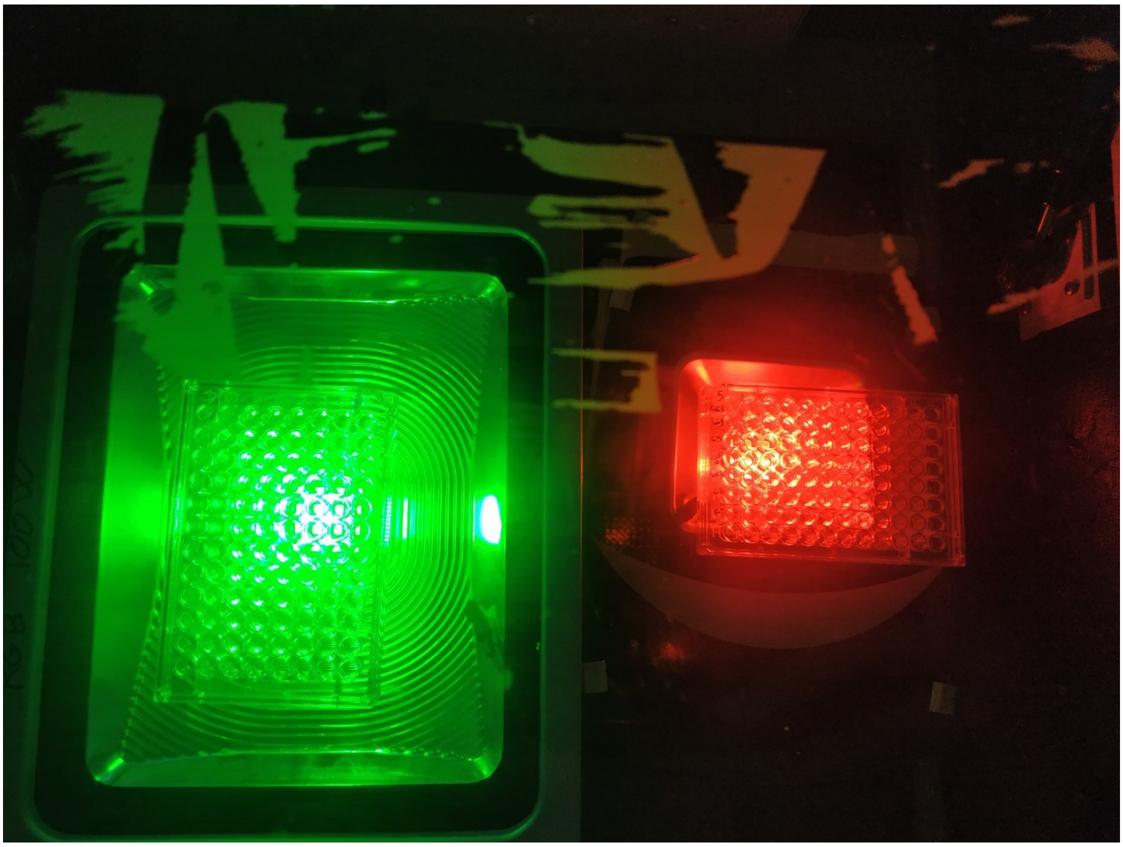

**Figure S9** . Irradiation of the photoreleasable quinolones in a biological environment. On the left, compound **2** irradiated with green light. On the right, compound **5** irradiated with red light.

## 7. Characterization data

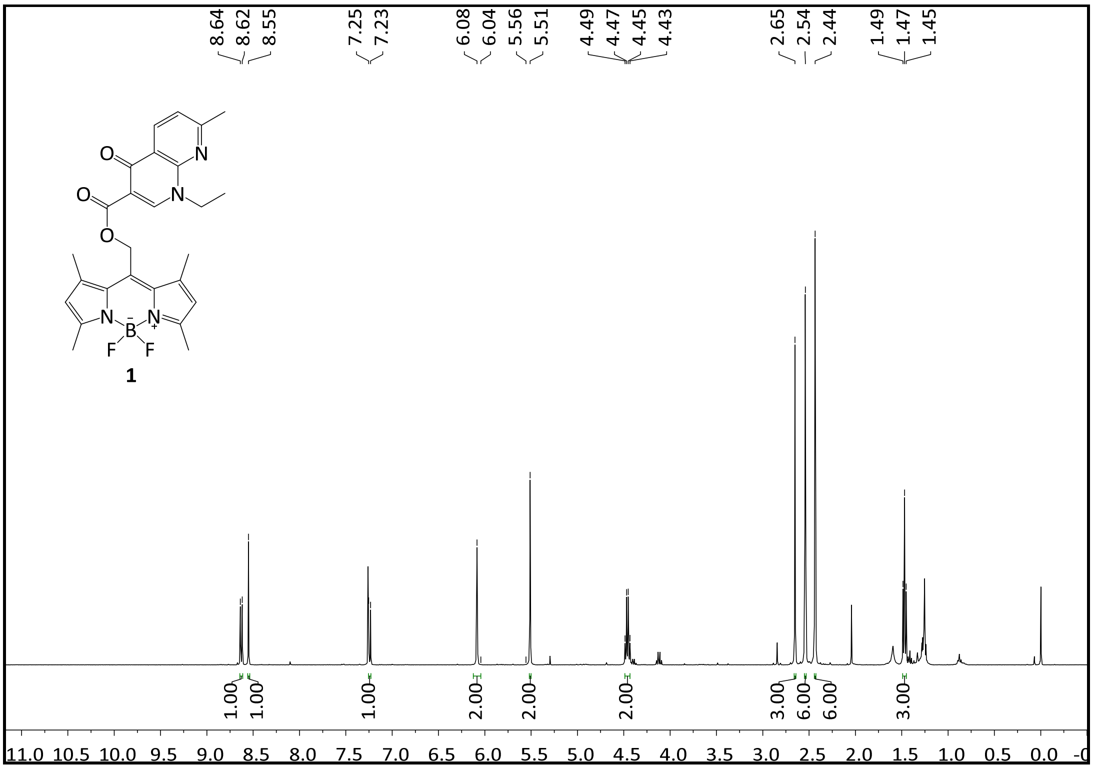


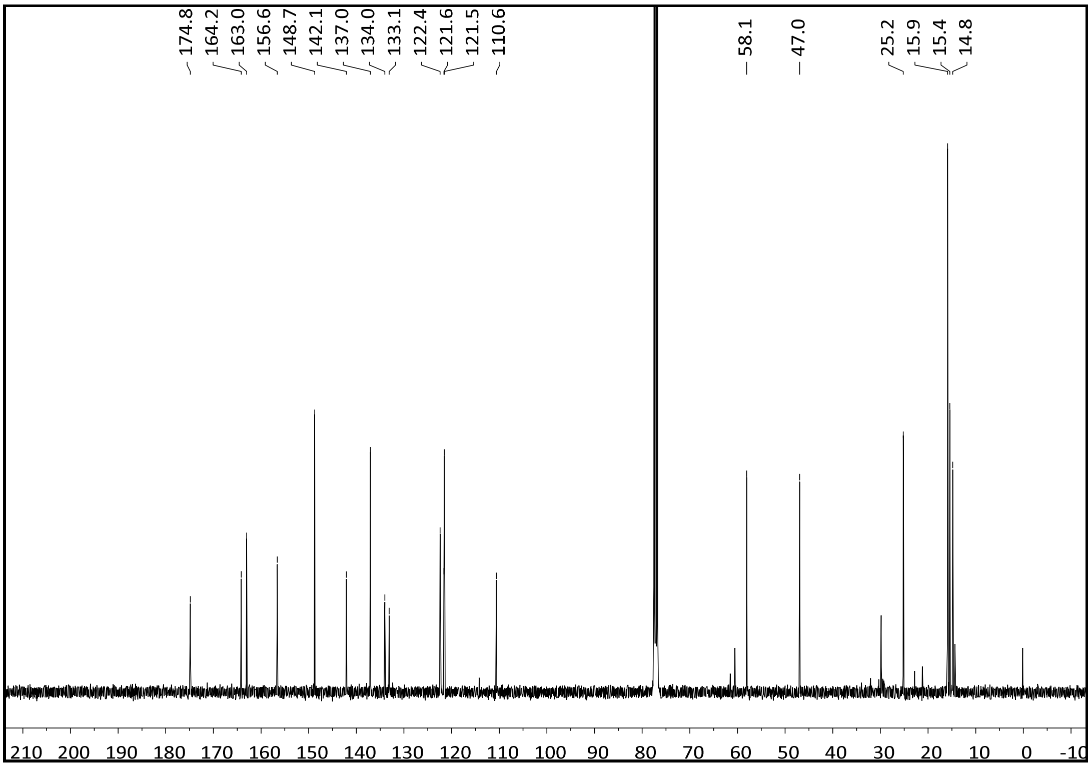

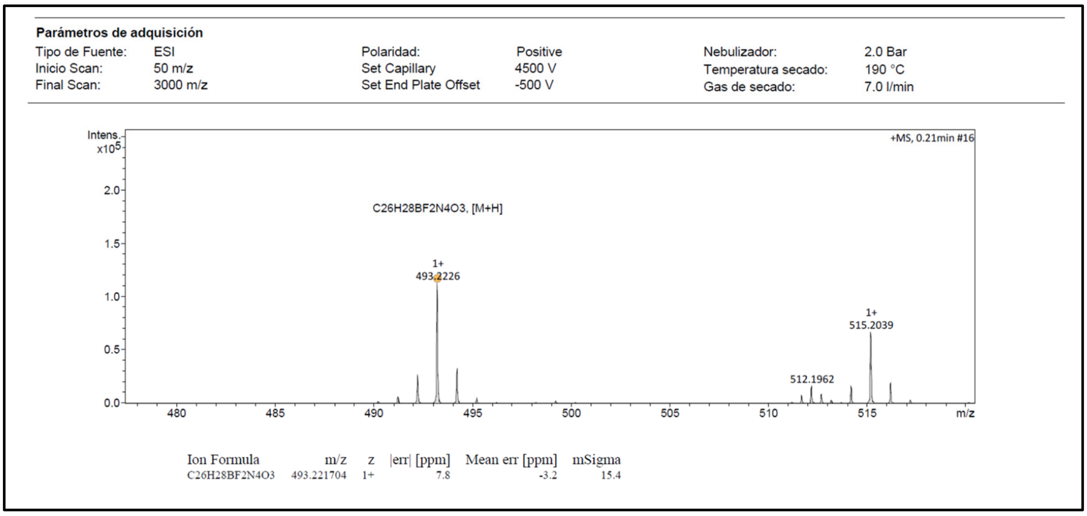
Parámetros de adquisición
Tipo de Fuente: ESI
Inicio Scan: 50 m/z
Final Scan: 3000 m/z
Polaridad: Positive
Set Capillary 4500 V
Set End Plate Offset -500 V
Nebulizador: 2.0 Bar
Temperatura secado: 190 °C
Gas de secado: 7.0 l/min
Intens. x10^5
+MS, 0.21min #16
C26H28BF2N4O3, [M+H]
1+ 493.2226
1+ 515.2039
512.1962
m/z
Ion Formula m/z z |err| [ppm] Mean err [ppm] mSigma
C26H28BF2N4O3 493.221704 1+ 7.8 -3.2 15.4

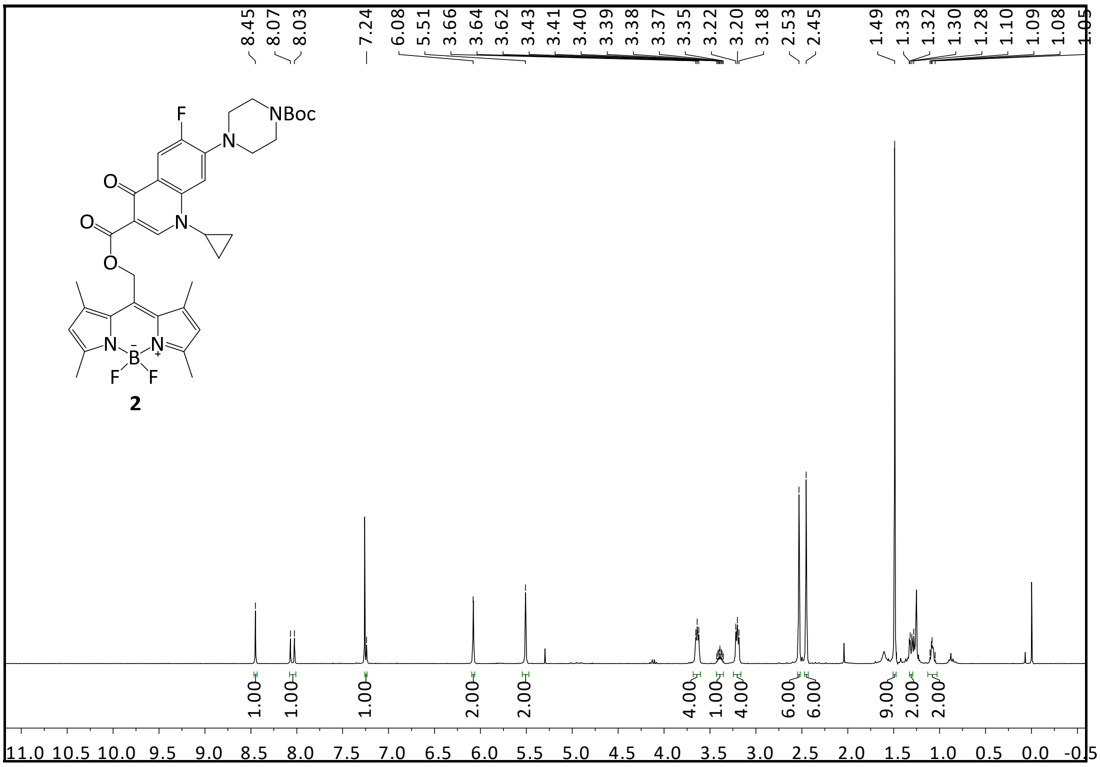
8.45 8.07 8.03 7.24 6.08 5.51 3.66 3.64 3.62 3.43 3.41 3.40 3.39 3.38 3.37 3.35 3.22 3.20 3.18 2.53 2.45 1.49 1.33 1.32 1.30 1.28 1.10 1.09 1.08 1.05
F
NBoc
N
O
O
N
O
N
B
N
F F
2
1.00 1.00 1.00 2.00 2.00 4.00 1.00 4.00 6.00 6.00 9.00 2.00 2.00
11.0 10.5 10.0 9.5 9.0 8.5 8.0 7.5 7.0 6.5 6.0 5.5 5.0 4.5 4.0 3.5 3.0 2.5 2.0 1.5 1.0 0.5 0.0 -0.5

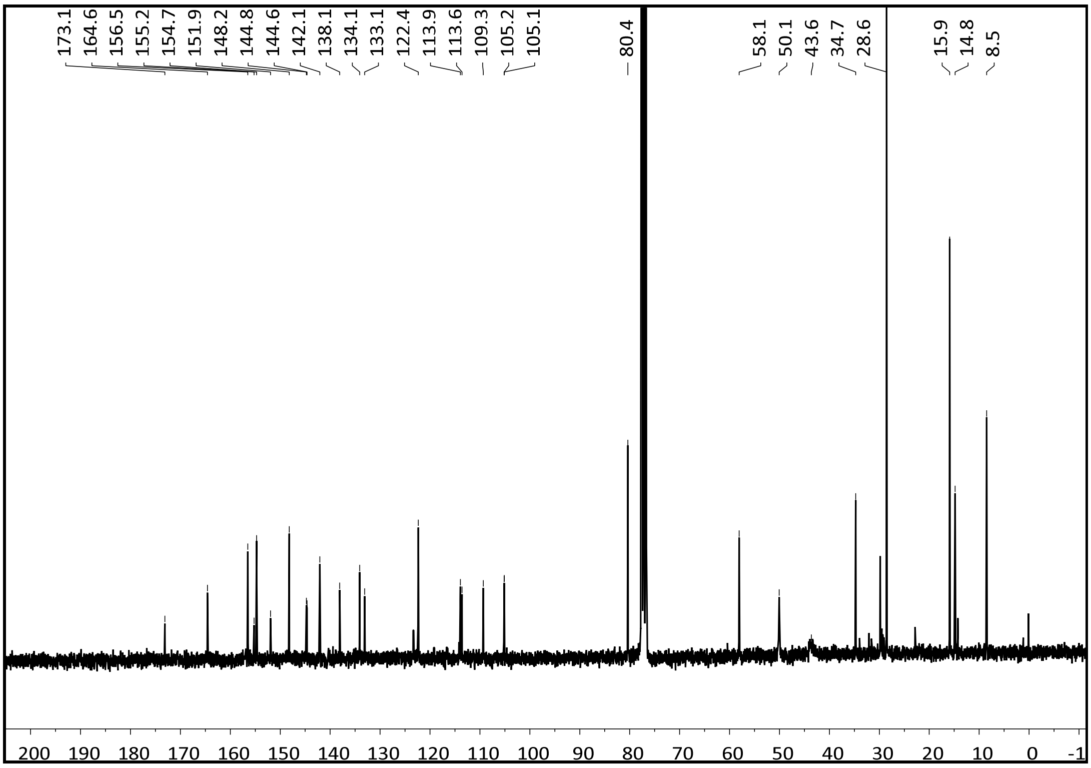


**Parámetros de adquisición**

| | | | | | |
|---|---|---|---|---|---|
| Tipo de Fuente: | ESI | Polaridad: | Positive | Nebulizador: | 2.0 Bar |
| Inicio Scan: | 50 m/z | Set Capillary | 4500 V | Temperatura secado: | 190 °C |
| Final Scan: | 3000 m/z | Set End Plate Offset | -500 V | Gas de secado: | 7.0 l/min |

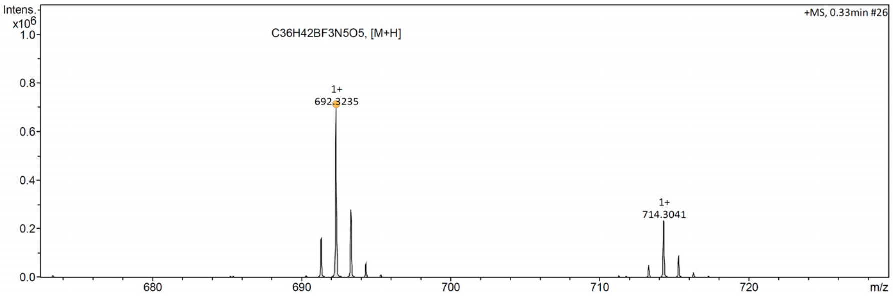


| Ion Formula | m/z | z | \|err\| [ppm] | Mean err [ppm] | mSigma |
|---|---|---|---|---|---|
| C36H42BF3N5O5 | 692.322561 | 1+ | 0.4 | 1.2 | 4.7 |

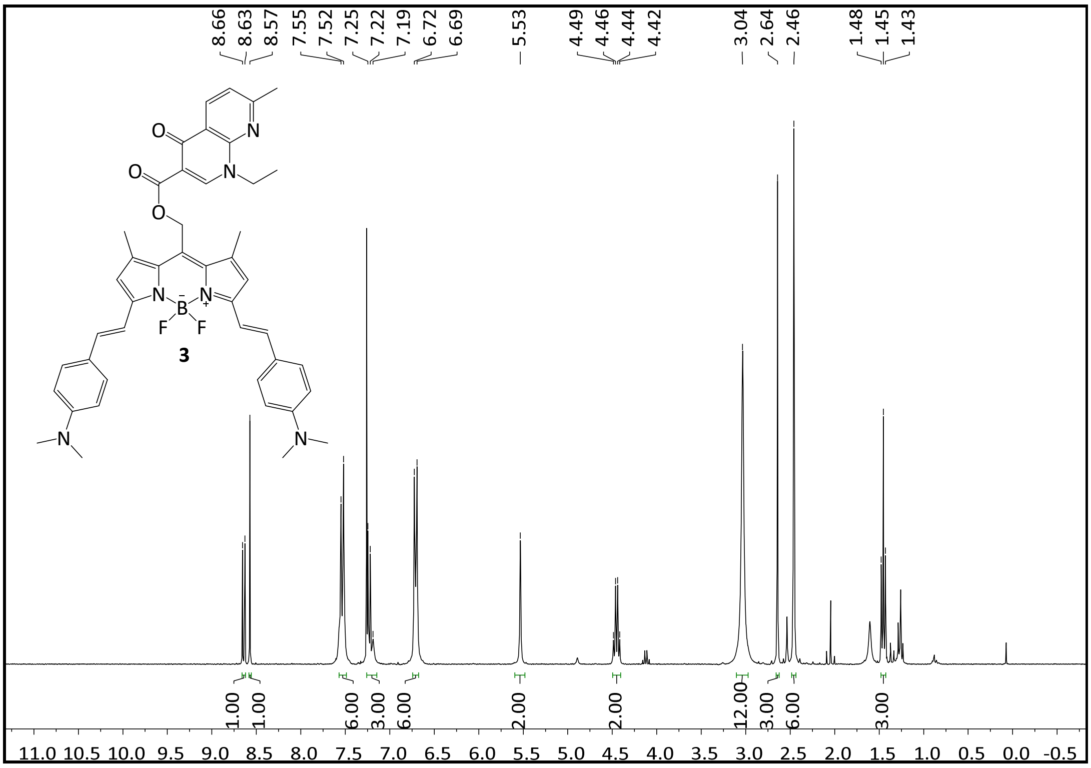
8.66
8.63
8.57
7.55
7.52
7.25
7.22
7.19
6.72
6.69
5.53
4.49
4.46
4.44
4.42
3.04
2.64
2.46
1.48
1.45
1.43
3
1.00
1.00
6.00
3.00
6.00
2.00
2.00
12.00
3.00
6.00
3.00
11.0 10.5 10.0 9.5 9.0 8.5 8.0 7.5 7.0 6.5 6.0 5.5 5.0 4.5 4.0 3.5 3.0 2.5 2.0 1.5 1.0 0.5 0.0 -0.5

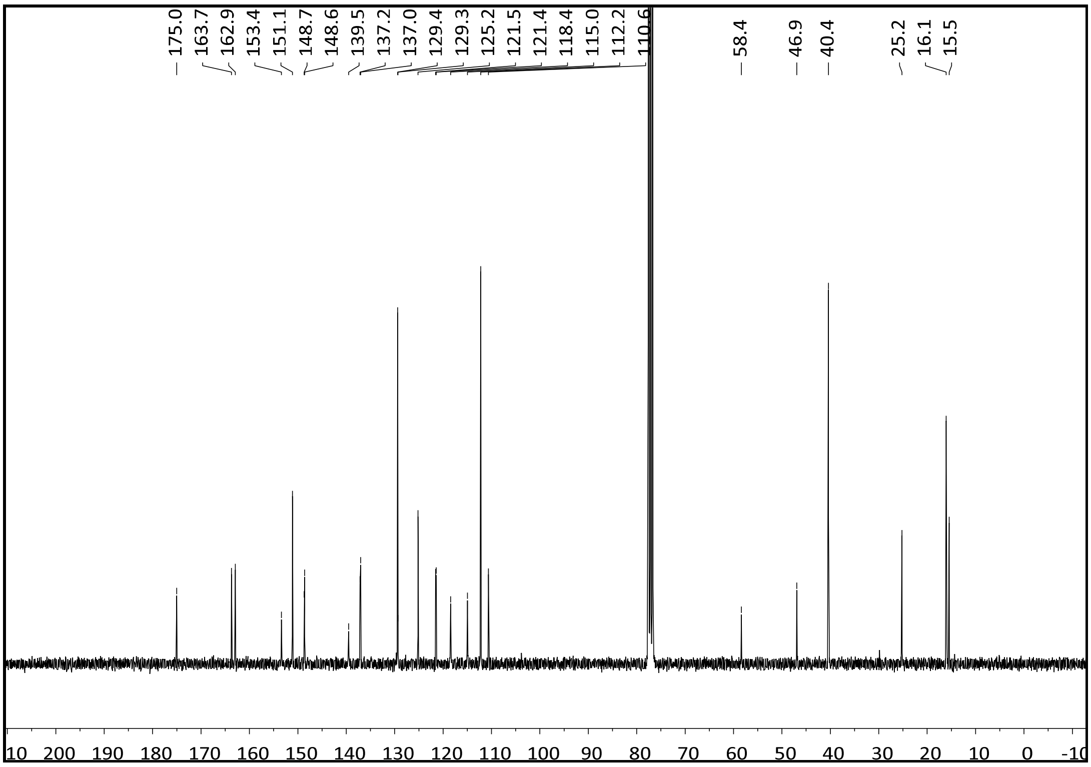
175.0
163.7
162.9
153.4
151.1
148.7
148.6
139.5
137.2
137.0
129.4
129.3
125.2
121.5
121.4
118.4
115.0
112.2
110.6
58.4
46.9
40.4
25.2
16.1
15.5
10 200 190 180 170 160 150 140 130 120 110 100 90 80 70 60 50 40 30 20 10 0 -10

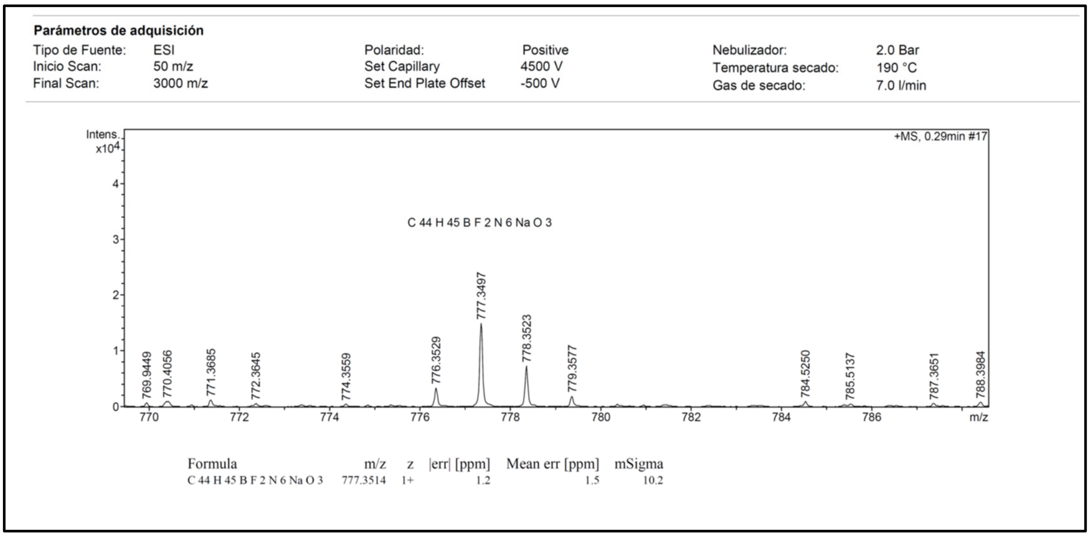
Parámetros de adquisición
Tipo de Fuente: ESI
Inicio Scan: 50 m/z
Final Scan: 3000 m/z
Polaridad: Positive
Set Capillary 4500 V
Set End Plate Offset -500 V
Nebulizador: 2.0 Bar
Temperatura secado: 190 °C
Gas de secado: 7.0 l/min
Intens. x10^4
+MS, 0.29min #17
C 44 H 45 B F 2 N 6 Na O 3
777.3497
778.3523
776.3529
779.3577
m/z
Formula m/z z |err| [ppm] Mean err [ppm] mSigma
C 44 H 45 B F 2 N 6 Na O 3 777.3514 1+ 1.2 1.5 10.2

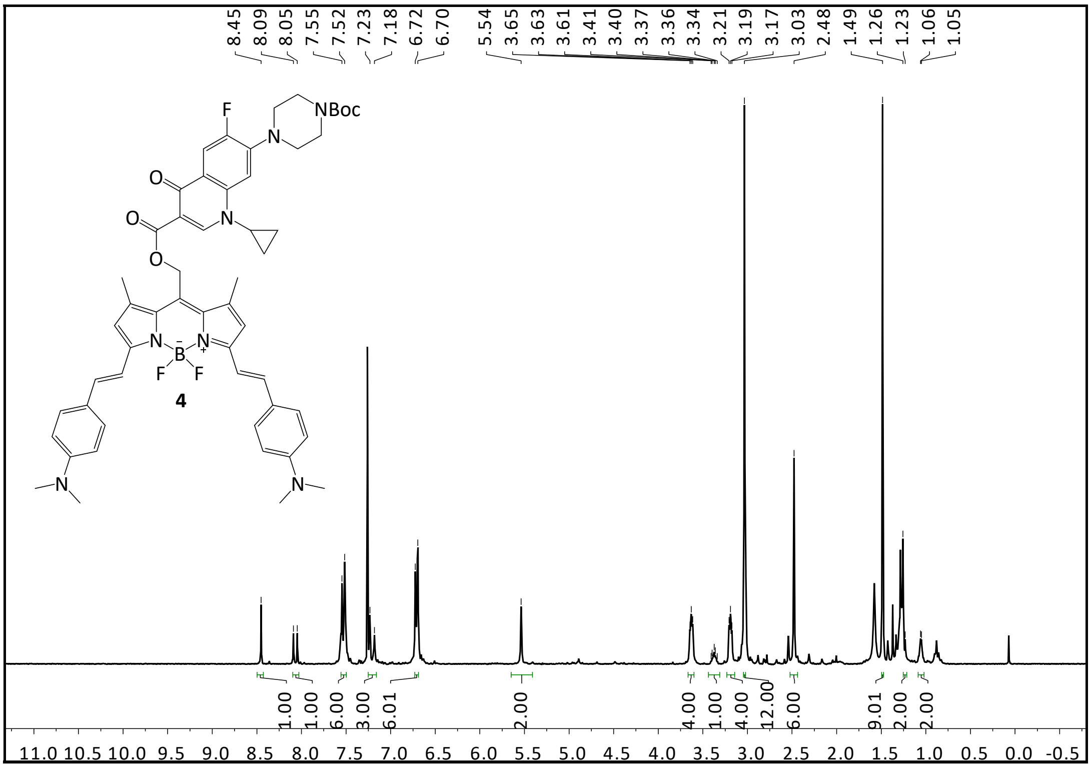
8.45 8.09 8.05 7.55 7.52 7.23 7.18 6.72 6.70 5.54 3.65 3.63 3.61 3.41 3.40 3.37 3.36 3.34 3.21 3.19 3.17 3.03 2.48 1.49 1.26 1.23 1.06 1.05
NBoc
4
1.00 1.00 6.00 3.00 6.01 2.00 4.00 1.00 4.00 12.00 6.00 9.01 2.00 2.00
11.0 10.5 10.0 9.5 9.0 8.5 8.0 7.5 7.0 6.5 6.0 5.5 5.0 4.5 4.0 3.5 3.0 2.5 2.0 1.5 1.0 0.5 0.0 -0.5

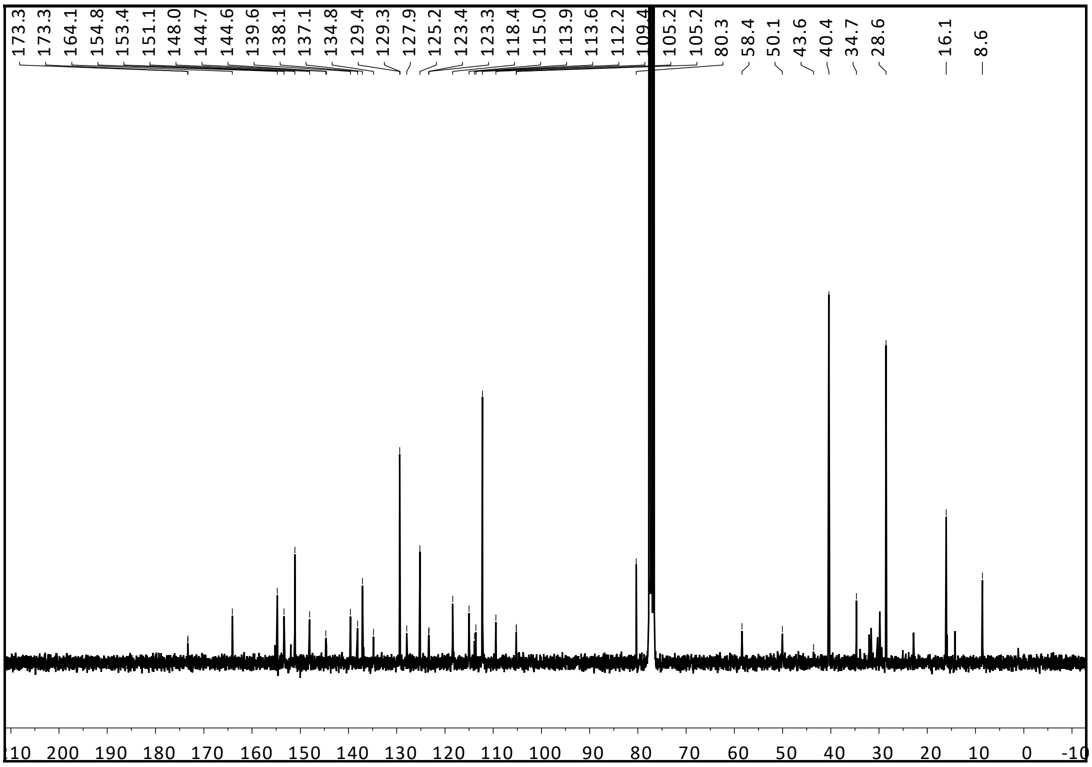
173.3
173.3
164.1
154.8
153.4
151.1
148.0
144.7
144.6
139.6
138.1
137.1
134.8
129.4
129.3
127.9
125.2
123.4
123.3
118.4
115.0
113.9
113.6
112.2
109.4
105.2
105.2
80.3
58.4
50.1
43.6
40.4
34.7
28.6
16.1
8.6
10 200 190 180 170 160 150 140 130 120 110 100 90 80 70 60 50 40 30 20 10 0 -10

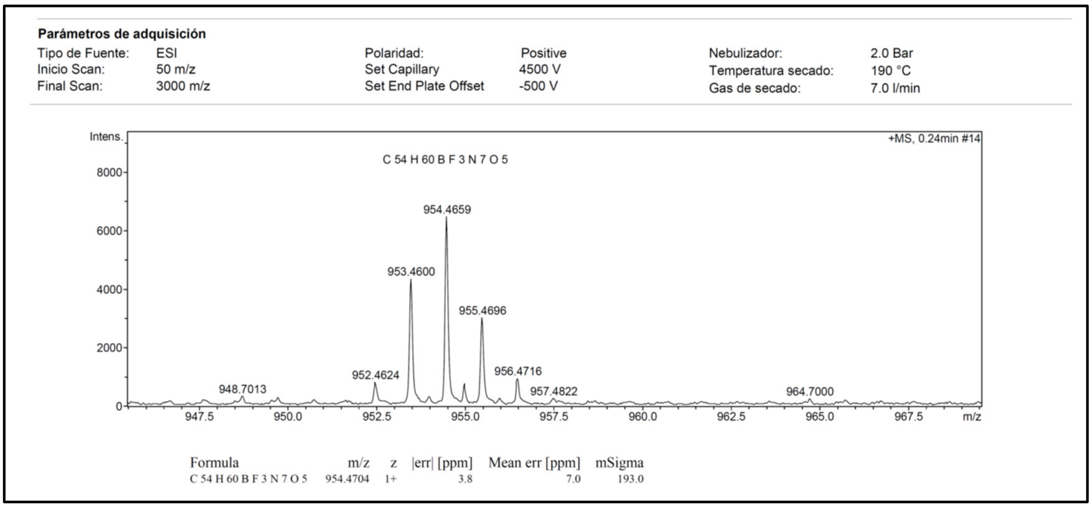
Parámetros de adquisición
Tipo de Fuente: ESI
Inicio Scan: 50 m/z
Final Scan: 3000 m/z
Polaridad: Positive
Set Capillary 4500 V
Set End Plate Offset -500 V
Nebulizador: 2.0 Bar
Temperatura secado: 190 °C
Gas de secado: 7.0 l/min
Intens.
+MS, 0.24min #14
C 54 H 60 B F 3 N 7 O 5
954.4659
953.4600
955.4696
952.4624
956.4716
957.4822
948.7013
964.7000
947.5 950.0 952.5 955.0 957.5 960.0 962.5 965.0 967.5 m/z
Formula m/z z |err| [ppm] Mean err [ppm] mSigma
C 54 H 60 B F 3 N 7 O 5 954.4704 1+ 3.8 7.0 193.0

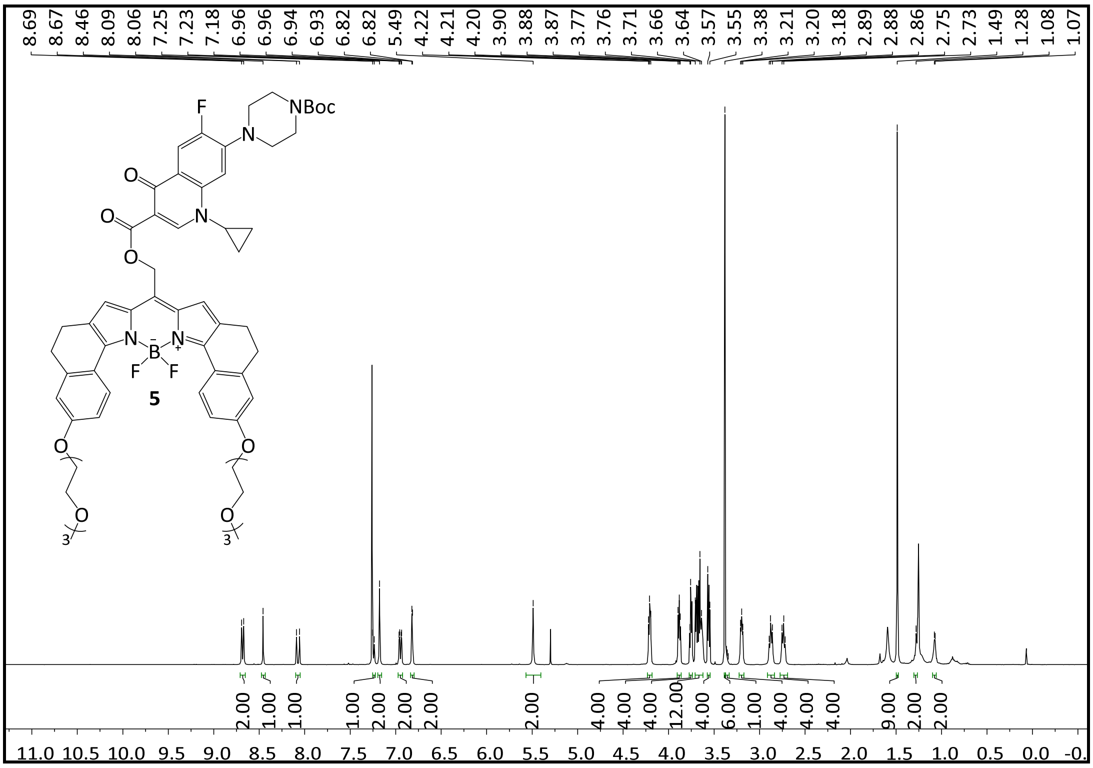

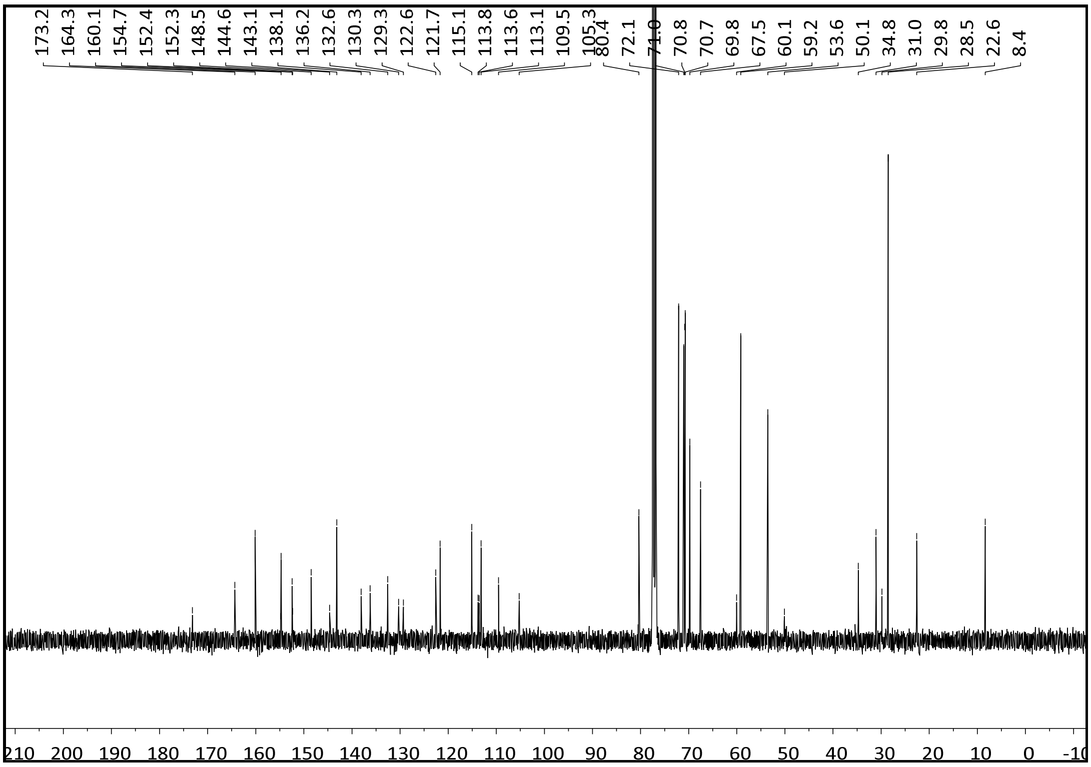

| | | | | | |
|---|---|---|---|---|---|
| **Sample Name** | 18 | **Position** | Vial 1 | **Instrument Name** | QTOF |
| **User Name** | CIF-PC\admin | **Inj Vol** | 1 | **InjPosition** | |
| **Sample Type** | Sample | **IRM Calibration Status** | Success | **Data Filename** | QTOF1918585.d |
| **ACQ Method** | ESI3.m | **Comment** | | **Acquired Time** | 11/19/2019 2:49:37 PM |

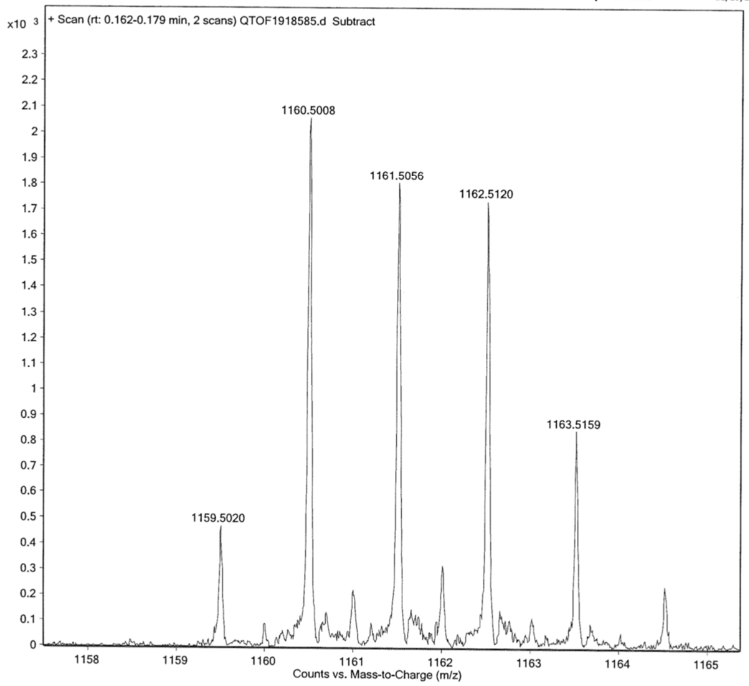


| | Formula (M) | m/z | m/z (Calc) | Diff (ppm) |
|---|---|---|---|---|
| ▸ | C62 H69 B F3 N5 O13 | 1159.502 | 1159.5046 | 2.25 |

## 8. Computational Results

### Computational Methods

The geometries have been optimized in the ground state ($S_0$) at the B3LYP[5,6]/6-31+g(d) level of theory. A single point calculation in the $S_0$ minimum has been performed at the CAM-B3LYP[7]/6-31+g(d) level of theory to compute the excitation energies and evaluate the electronic nature of the $S_0 \rightarrow S_1$ transition. To analyze the photorelease path, both optimizations and relaxed scans along the photorelease coordinate (C-O distance) have been performed in $S_1$ and $T_1$. Calculations in the ground state ($S_0$) and first triplet state ($T_1$) have been performed with the B3LYP functional whereas the ones performed in the first singlet excited state ($S_1$) were performed with the CAM-B3LYP functional. In all cases, the 6-31+g(d) has been selected as the basis set. Moreover, we have also evaluated the solvent effect considering it in two different ways: on one hand implicitly (dichloromethane, DMSO and water) by applying the polarizable continuum model through the integral equation formalism variant (IEF-PCM[8]); on the other hand, two water molecules were explicitly included while keeping the IEF-PCM implicit water description for the surroundings. Frequency calculations have been performed to verify the minimum or transition state character of the optimized stationary points and to get the free energies. The density functional theory (DFT) and its time-dependent version (TD-DFT) were applied for ground and excited states, respectively, as implemented in Gaussian 16 software.[9] It should be remarked that for **5**, the tetraoxaundecane chain present in the synthetic analogue has been simplified with a methoxy group, to decrease the computational time. This simplification should not influence the absorption energy neither the mechanism, as the chain does not contribute to the electronic conjugation extent of the chromophore. The spin-orbit coupling values were computed at the CAM-B3LYP/def2-SVPD level of theory, including water as implicit solvent by the conductor-like polarizable continuum model, CPCM, as implemented in Orca 4.2.1.[12]

The electronic nature of the vertical transition has been analyzed for the three compounds studied from a computational point of view, compounds **2**, **4** and **5**. In all cases the first singlet excited state is optically bright (large oscillation strength value, f, in Table S4) and corresponds to a $\pi,\pi^*$ electronic transition (Figure S10). As experimentally observed, the computed absorption energy of **4** is smaller (that is the wavelength is longer) compared to **5**, whereas the one computed for compound **2** is the largest (shorter wavelength).

**Table S4.** Experimental and computed absorption ($S_0 \rightarrow S_1$) energies in eV (nm) and the corresponding oscillator strength (f), considering dichloromethane as the solvent.

| Compound | 2 | 4 | 5 |
|---|---|---|---|
| **Experimental $\lambda_{max}$ eV (nm)** | 2.38 (520) | 1.67 (744) | 1.85 (671) |
| **Computed Excitation energy eV (nm)** | 2.74 (452) | 1.87 (663) | 2.09 (592) |
| ***f*** | 0.628 | 1.197 | 0.904 |

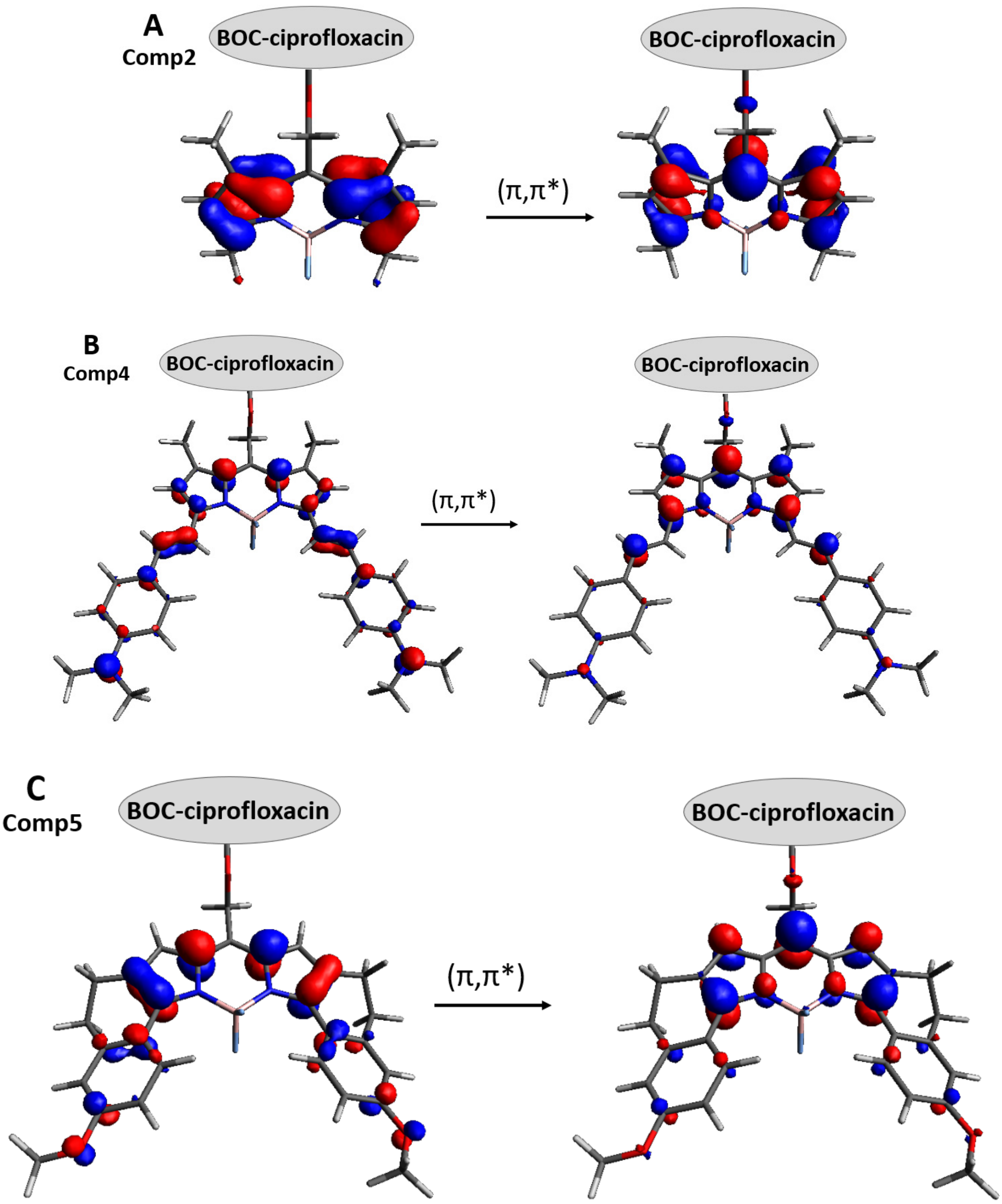


**Figure S10.** Molecular orbitals involved in the $S_0 \rightarrow S_1$ vertical transition of compound A) **2**, B) **4** and C) **5**.

In addition, relaxed scans have been performed from the $S_1$ and $T_1$ minima (Figures S11 and S12, respectively), considering diverse solvents. In particular, the solvents tested are implicit dichloromethane, DMSO and water and explicit water.

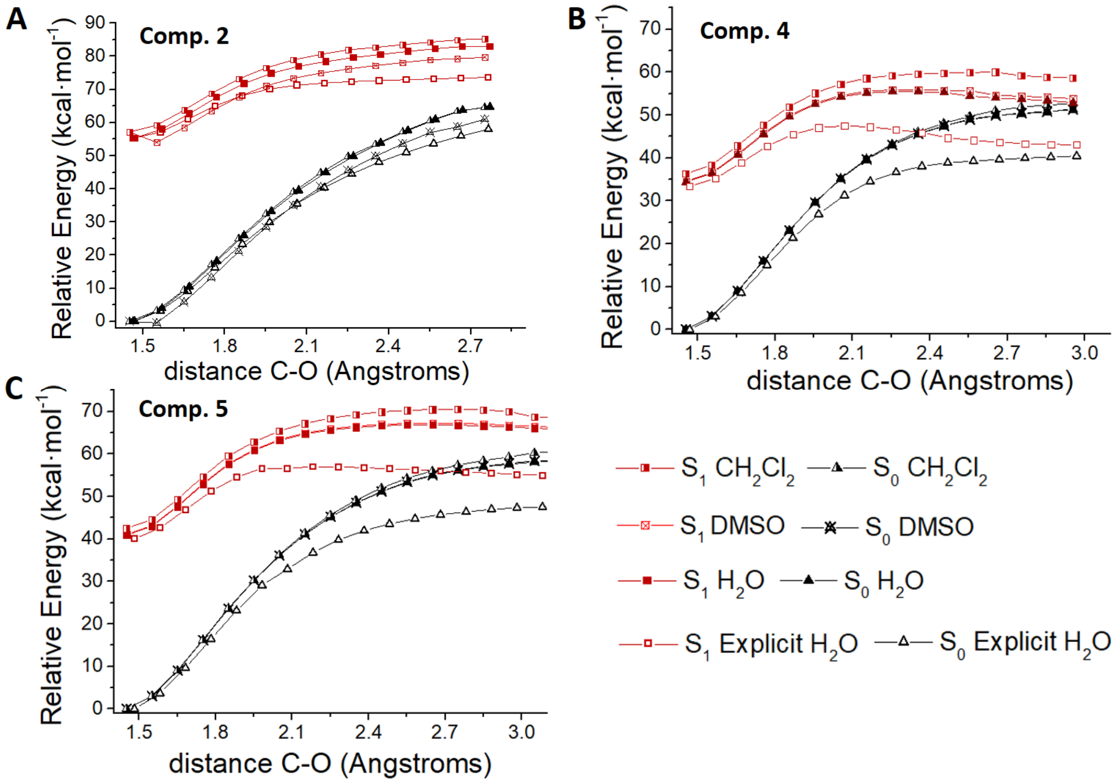


**Figure S11.** Relaxed scan along the photorelease coordinate (distance C-O) in $S_1$ considering diverse solvents for A) compound **2**, B) **4** and C) **5**.

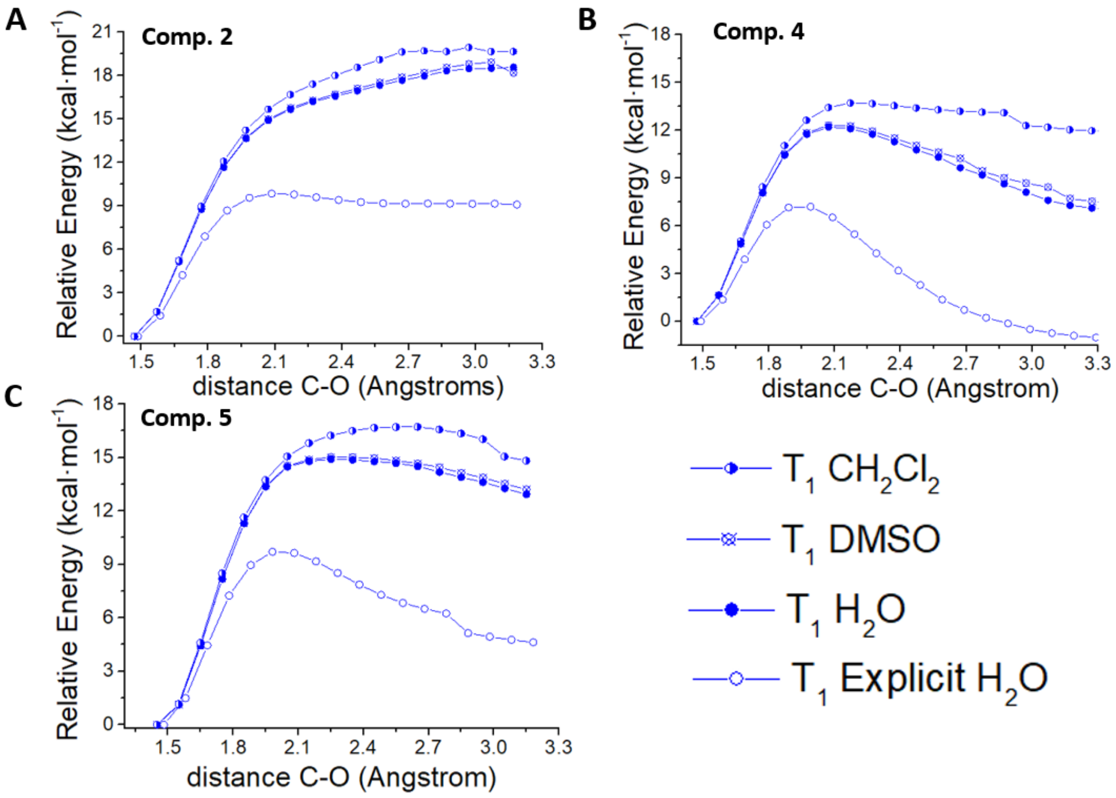


**Figure S12.** Relaxed scan along the photorelease coordinate (distance C-O) in $T_1$ considering diverse solvents for A) compound **2**, B) **4** and C) **5**.

**Table S5.** Energy of the first triplet ($T_1$) and singlet excited state ($S_1$) relative to the ground state ($S_0$) computed at the $S_1$ minimum. The results shown have been computed considering explicit water as the solvent.

| Comp. | Solvent | Relative $S_0$ energy (kcal/mol) | Relative $S_1$ energy (kcal/mol) | Relative $T_1$ energy (kcal/mol) | $\Delta E_{S1-T1}$ (kcal/mol) |
|---|---|---|---|---|---|
| **2** | Explicit water | 0 | 55.3 | 29.7 | 25.6 |
| | Implicit water | 0 | 55.5 | 30.5 | 25.0 |
| **4** | Explicit water | 0 | 33.4 | 16.7 | 16.7 |
| | Implicit water | 0 | 34.4 | 17.7 | 16.7 |
| **5** | Explicit water | 0 | 45.9 | 21.7 | 24.2 |
| | Implicit water | 0 | 46.5 | 22.6 | 23.9 |

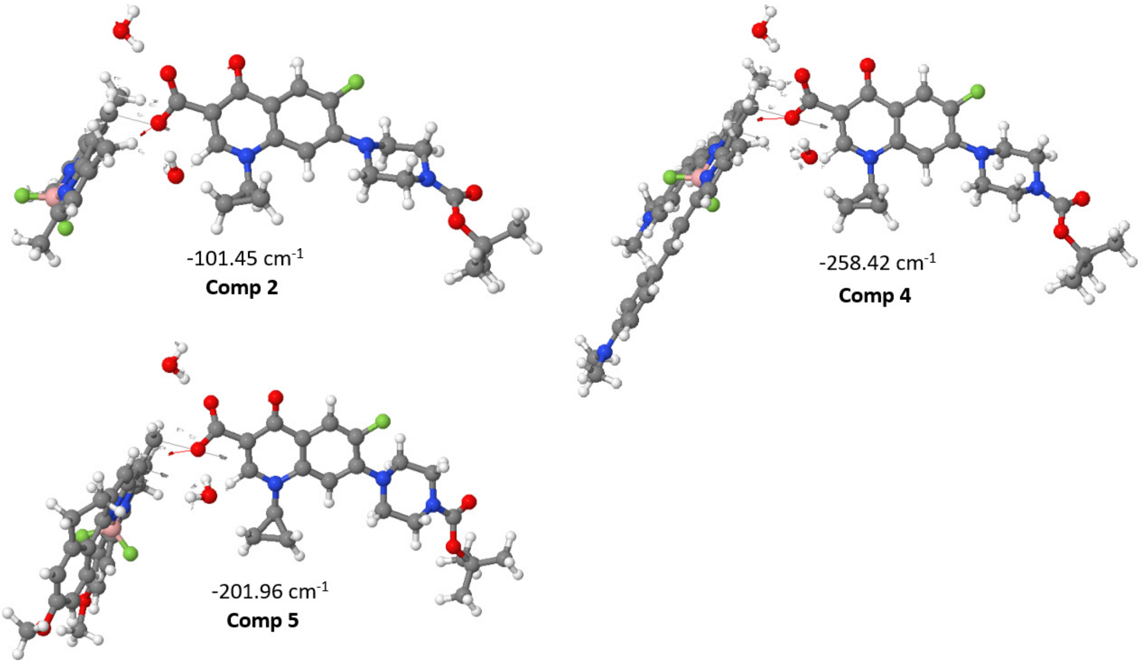


**Figure S13.** Transition state structures optimized in $T_1$ considering explicit water molecules as the solvent. The vector of the negative frequency corresponds to the photorelease process.

**Table S6.** Charge analysis for the ciprofloxacin, PPG and, when applying, the water molecules computed for the minimum, transition state and products in $T_1$. The charge of each moiety is computed as the sum of the Mulliken charges of each atom included in the moiety.

| | | **Min $T_1$** | | **TS $T_1$** | | **Products $T_1$** | |
|---|---|---|---|---|---|---|---|
| | | Implicit water | Explicit water | Implicit water | Explicit water | Implicit water | Explicit water |
| **2** | *Ciprofloxacin* | -0.59 | -0.45 | - | -0.87 | -0.97 | -0.97 |
| | *PPG* | 0.59 | 0.25 | - | 0.76 | 0.97 | 1.00 |
| | *WAT1* | | 0.16 | | 0.09 | | -0.006 |
| | *WAT2* | | 0.04 | | 0.02 | | -0.03 |
| | | | | | | | |
| **4** | *Ciprofloxacin* | -0.71 | -0.47 | -0.85 | -0.81 | -1.0 | -0.98 |
| | *PPG* | 0.71 | 0.26 | 0.85 | 0.64 | 1.0 | 1.00 |
| | *WAT1* | - | 0.17 | - | 0.14 | | 0.00 |
| | *WAT2* | - | 0.04 | - | 0.03 | | -0.02 |
| | | | | | | | |
| **5** | *Ciprofloxacin* | -0.49 | -0.43 | -0.87 | -0.81 | -0.95 | -0.94 |
| | *PPG* | 0.49 | 0.24 | 0.87 | 0.68 | 0.95 | 0.99 |
| | *WAT1* | - | 0.16 | - | 0.11 | - | -0.01 |
| | *WAT2* | - | 0.03 | - | 0.02 | - | -0.04 |

**Table S7.** $S_1$- $T_1$ spin-orbit coupling values (in $cm^{-1}$) calculated at the CAM-B3LYP/def2-SVPD level of theory, including water as implicit solvent, without (implicit) or with (micro-solvation) two explicit water molecules.

| Solvation method | **Compound 2** | **Compound 4** | **Compound 5** |
|---|---|---|---|
| Micro-solvation | 0.118 | 0.024 | 0.037 |
| Implicit | 0.118 | 0.022 | 0.051 |

## 9. Cartesian Coordinates

| **Compound 2** | | | | | | | |
|---|---|---|---|---|---|---|---|
| Min $S_0$ (implicit water) | | | | Min $S_1$ (implicit water) | | | |
| 6 | -1.948701 | 0.940695 | -0.221898 | 6 | -2.192738 | 0.949198 | -0.294893 |
| 6 | -2.556749 | 2.120571 | 0.248514 | 6 | -2.696932 | 2.179861 | 0.177083 |
| 6 | -2.500179 | 3.083773 | -0.755056 | 6 | -2.501460 | 3.146427 | -0.793010 |
| 7 | -1.511791 | 1.147623 | -1.486180 | 7 | -1.685472 | 1.135118 | -1.537707 |
| 6 | -1.833667 | 2.461177 | -1.866237 | 6 | -1.862971 | 2.472378 | -1.900543 |
| 1 | -2.988178 | 2.247429 | 1.233678 | 1 | -3.150449 | 2.327058 | 1.149402 |
| 6 | -3.039562 | 4.477490 | -0.601256 | 6 | -2.874019 | 4.588524 | -0.657052 |
| 1 | -3.357468 | 4.632197 | 0.434215 | 1 | -3.119680 | 4.804772 | 0.386081 |
| 1 | -3.914867 | 4.656546 | -1.238505 | 1 | -3.754217 | 4.851362 | -1.256555 |
| 1 | -2.293508 | 5.243376 | -0.837279 | 1 | -2.059744 | 5.255993 | -0.955006 |
| 5 | -0.798782 | 0.109968 | -2.380905 | 5 | -1.062326 | 0.046956 | -2.419141 |
| 7 | -0.425213 | 0.811826 | -3.704715 | 7 | -0.501427 | 0.739374 | -3.666234 |
| 6 | -1.477890 | 2.935858 | -3.140604 | 6 | -1.438218 | 2.944427 | -3.167046 |
| 6 | -0.766136 | 2.130829 | -4.046897 | 6 | -0.687539 | 2.082040 | -4.003710 |
| 6 | -1.868398 | 4.337648 | -3.545115 | 6 | -1.764866 | 4.331723 | -3.607130 |
| 6 | 0.258970 | 0.258287 | -4.733387 | 6 | 0.226128 | 0.141492 | -4.640792 |
| 6 | -0.245847 | 2.386841 | -5.362101 | 6 | -0.005737 | 2.318391 | -5.255808 |
| 6 | 0.383966 | 1.211446 | -5.762278 | 6 | 0.540095 | 1.102271 | -5.625457 |
| 6 | -0.298164 | 3.630815 | -6.202459 | 6 | 0.136575 | 3.593552 | -6.024261 |
| 1 | 0.894320 | 1.047342 | -6.703179 | 1 | 1.125724 | 0.903963 | -6.514535 |
| 1 | -1.313510 | 3.849396 | -6.557041 | 1 | -0.788876 | 3.875933 | -6.541029 |
| 1 | 0.331178 | 3.498553 | -7.087706 | 1 | 0.908027 | 3.473416 | -6.789599 |
| 1 | 0.064084 | 4.513294 | -5.665388 | 1 | 0.426837 | 4.432267 | -5.384233 |
| 8 | -0.777804 | 5.215829 | -3.173539 | 8 | -0.707969 | 5.227135 | -3.175656 |
| 1 | -2.778472 | 4.661810 | -3.045843 | 1 | -2.704854 | 4.676939 | -3.181358 |
| 1 | -2.040386 | 4.412427 | -4.616611 | 1 | -1.853492 | 4.399320 | -4.689441 |
| 6 | -0.951197 | 6.540392 | -3.462479 | 6 | -0.865823 | 6.532236 | -3.498112 |
| 6 | 0.186824 | 7.361544 | -3.002821 | 6 | 0.240524 | 7.369898 | -2.999135 |
| 8 | -1.952385 | 6.942615 | -4.031755 | 8 | -1.831464 | 6.918593 | -4.128001 |
| 6 | 0.362453 | 8.743632 | -3.442136 | 6 | 0.333806 | 8.786986 | -3.313231 |
| 6 | 1.100170 | 6.798022 | -2.130427 | 6 | 1.205432 | 6.783827 | -2.212325 |
| 7 | 2.158941 | 7.441447 | -1.601568 | 7 | 2.252611 | 7.424716 | -1.673181 |
| 1 | 0.987048 | 5.770140 | -1.814947 | 1 | 1.147753 | 5.728901 | -1.983290 |
| 6 | 1.557127 | 9.407863 | -2.882861 | 6 | 1.519633 | 9.454842 | -2.747073 |
| 8 | -0.387498 | 9.341365 | -4.235658 | 8 | -0.475537 | 9.419882 | -4.003367 |
| 6 | 2.435518 | 8.765000 | -1.984847 | 6 | 2.456677 | 8.781608 | -1.952439 |
| 6 | 3.070644 | 6.722767 | -0.718664 | 6 | 3.225955 | 6.681420 | -0.893031 |
| 6 | 2.525213 | 5.781336 | 0.321483 | 6 | 2.754756 | 5.657890 | 0.095057 |

| | | | | | | | |
|---|---|---|---|---|---|---|---|
| 6 | 3.148916 | 7.088897 | 0.742785 | 6 | 3.353169 | 6.947578 | 0.578958 |
| 1 | 3.994524 | 6.439093 | -1.217218 | 1 | 4.133958 | 6.465633 | -1.449193 |
| 1 | 2.475967 | 7.864150 | 1.098119 | 1 | 2.673527 | 7.675694 | 1.010368 |
| 1 | 4.131652 | 7.086267 | 1.205520 | 1 | 4.352235 | 6.944546 | 1.002587 |
| 1 | 3.073984 | 4.857669 | 0.478700 | 1 | 3.336102 | 4.745127 | 0.168425 |
| 1 | 1.448432 | 5.690226 | 0.427918 | 1 | 1.686796 | 5.525307 | 0.233505 |
| 6 | 1.844288 | 10.735682 | -3.253251 | 6 | 1.726893 | 10.817445 | -3.010027 |
| 6 | 2.952292 | 11.369952 | -2.755309 | 6 | 2.823099 | 11.453735 | -2.506484 |
| 6 | 3.874842 | 10.745128 | -1.875437 | 6 | 3.806717 | 10.798496 | -1.730830 |
| 6 | 3.580420 | 9.426105 | -1.505800 | 6 | 3.591290 | 9.448521 | -1.467168 |
| 1 | 1.167566 | 11.258963 | -3.919934 | 1 | 0.997694 | 11.361984 | -3.598642 |
| 1 | 4.257210 | 8.900428 | -0.848735 | 1 | 4.318941 | 8.900266 | -0.888438 |
| 9 | 3.154348 | 12.676790 | -3.093686 | 9 | 2.958297 | 12.786110 | -2.731615 |
| 7 | 4.972488 | 11.452257 | -1.373241 | 7 | 4.895142 | 11.512216 | -1.225612 |
| 6 | 5.919393 | 12.020832 | -2.354845 | 6 | 5.761845 | 12.178611 | -2.208122 |
| 6 | 5.679101 | 10.887980 | -0.219409 | 6 | 5.681865 | 10.883107 | -0.170184 |
| 6 | 6.786528 | 13.097616 | -1.708333 | 6 | 6.626841 | 13.229319 | -1.531753 |
| 1 | 5.370447 | 12.459885 | -3.186881 | 1 | 5.153741 | 12.658020 | -2.973018 |
| 1 | 6.562195 | 11.217052 | -2.751517 | 1 | 6.405812 | 11.430618 | -2.697812 |
| 6 | 6.544021 | 11.957139 | 0.451713 | 6 | 6.547820 | 11.920374 | 0.533633 |
| 1 | 6.314438 | 10.035773 | -0.516113 | 1 | 6.324124 | 10.082731 | -0.573115 |
| 1 | 4.946396 | 10.533918 | 0.510647 | 1 | 5.006323 | 10.443699 | 0.567093 |
| 7 | 7.452233 | 12.569931 | -0.518398 | 7 | 7.372280 | 12.636545 | -0.430981 |
| 1 | 7.122431 | 11.501648 | 1.253255 | 1 | 7.187479 | 11.426196 | 1.261080 |
| 1 | 5.894861 | 12.731811 | 0.886752 | 1 | 5.902588 | 12.633944 | 1.064528 |
| 1 | 6.160515 | 13.959150 | -1.430621 | 1 | 5.991687 | 14.042160 | -1.152479 |
| 1 | 7.545747 | 13.434839 | -2.414022 | 1 | 7.333312 | 13.647148 | -2.247305 |
| 6 | 8.738329 | 12.947801 | -0.240673 | 6 | 8.655505 | 13.028643 | -0.200834 |
| 8 | 9.438824 | 13.596789 | -1.018977 | 8 | 9.287596 | 13.757254 | -0.959622 |
| 8 | 9.135396 | 12.505189 | 0.972133 | 8 | 9.139073 | 12.509510 | 0.938534 |
| 6 | 10.507289 | 12.724058 | 1.487511 | 6 | 10.522102 | 12.736216 | 1.378545 |
| 6 | 10.456407 | 12.034386 | 2.853839 | 6 | 10.586365 | 11.948260 | 2.681868 |
| 6 | 10.776297 | 14.223186 | 1.650790 | 6 | 10.754529 | 14.220659 | 1.639703 |
| 6 | 11.529212 | 12.037933 | 0.575935 | 6 | 11.498737 | 12.161821 | 0.357823 |
| 1 | 11.271746 | 10.981502 | 0.438388 | 1 | 11.263769 | 11.112144 | 0.154107 |
| 1 | 12.519249 | 12.087260 | 1.043466 | 1 | 12.513630 | 12.209340 | 0.764861 |
| 1 | 11.582592 | 12.520689 | -0.401836 | 1 | 11.472810 | 12.719862 | -0.578814 |
| 1 | 9.991323 | 14.689056 | 2.257454 | 1 | 9.998692 | 14.608911 | 2.329972 |
| 1 | 10.825595 | 14.729438 | 0.684858 | 1 | 10.721969 | 14.798848 | 0.715797 |
| 1 | 11.732403 | 14.363468 | 2.167936 | 1 | 11.737404 | 14.354812 | 2.102321 |
| 1 | 9.696035 | 12.494742 | 3.494032 | 1 | 9.856233 | 12.330705 | 3.401441 |
| 1 | 11.427891 | 12.125151 | 3.351139 | 1 | 11.584237 | 12.037318 | 3.120822 |
| 1 | 10.222594 | 10.969875 | 2.743855 | 1 | 10.379594 | 10.888630 | 2.504179 |
| 9 | 0.364519 | -0.372352 | -1.737569 | 9 | -0.033218 | -0.617684 | -1.723086 |
| 9 | -1.655887 | -0.989256 | -2.634426 | 9 | -2.032288 | -0.915231 | -2.779738 |
| 6 | -1.781977 | -0.347633 | 0.516405 | 6 | -2.184241 | -0.359118 | 0.404704 |
| 1 | -2.190151 | -1.190068 | -0.052263 | 1 | -2.640475 | -1.142475 | -0.211200 |
| 1 | -0.723165 | -0.563922 | 0.700317 | 1 | -1.159325 | -0.685277 | 0.619309 |
| 1 | -2.296204 | -0.292559 | 1.479269 | 1 | -2.731791 | -0.284189 | 1.346032 |
| 6 | 0.781690 | -1.141507 | -4.728052 | 6 | 0.604993 | -1.292715 | -4.615515 |
| 1 | 1.531022 | -1.281654 | -3.940617 | 1 | 1.279108 | -1.508071 | -3.777655 |
| 1 | -0.019413 | -1.865964 | -4.544596 | 1 | -0.273947 | -1.934102 | -4.484084 |
| 1 | 1.244314 | -1.368379 | -5.691947 | 1 | 1.105901 | -1.564314 | -5.546531 |
| Min $T_1$ (implicit water) | | | | Products $T_1$ (implicit water) | | | |
| 6 | -1.970151 | 0.875856 | -0.219058 | 6 | -1.730941 | 0.065024 | -0.725867 |
| 6 | -2.494678 | 2.078312 | 0.286860 | 6 | -2.081808 | 1.306839 | -0.128203 |
| 6 | -2.408651 | 3.057337 | -0.692045 | 6 | -2.375708 | 2.211016 | -1.121626 |
| 7 | -1.547988 | 1.096818 | -1.522510 | 7 | -1.792077 | 0.198972 | -2.086243 |

| | | | | | | | |
|---|---|---|---|---|---|---|---|
| 6 | -1.802696 | 2.409070 | -1.859537 | 6 | -2.191631 | 1.481234 | -2.380546 |
| 1 | -2.890152 | 2.207092 | 1.286798 | 1 | -2.102377 | 1.490538 | 0.937874 |
| 6 | -2.845515 | 4.480112 | -0.519683 | 6 | -2.755765 | 3.639283 | -0.909110 |
| 1 | -3.081740 | 4.664619 | 0.532821 | 1 | -2.679218 | 3.878624 | 0.155197 |
| 1 | -3.748325 | 4.712768 | -1.100042 | 1 | -3.792536 | 3.831877 | -1.214244 |
| 1 | -2.068936 | 5.192554 | -0.819353 | 1 | -2.101930 | 4.325896 | -1.466304 |
| 5 | -0.918151 | 0.040161 | -2.445161 | 5 | -1.506357 | -0.931868 | -3.113413 |
| 7 | -0.525135 | 0.752166 | -3.750105 | 7 | -1.498798 | -0.258486 | -4.516008 |
| 6 | -1.480009 | 2.918943 | -3.148238 | 6 | -2.351225 | 1.914212 | -3.752136 |
| 6 | -0.790363 | 2.069161 | -4.058116 | 6 | -1.895450 | 1.027737 | -4.800805 |
| 6 | -1.842464 | 4.315414 | -3.532241 | 6 | -2.943075 | 3.125194 | -4.052085 |
| 6 | 0.143091 | 0.165547 | -4.815936 | 6 | -1.130462 | -0.867644 | -5.682193 |
| 6 | -0.241743 | 2.332173 | -5.392443 | 6 | -1.745262 | 1.250443 | -6.241425 |
| 6 | 0.316439 | 1.135975 | -5.818484 | 6 | -1.274197 | 0.064587 | -6.749916 |
| 6 | -0.231799 | 3.608446 | -6.177102 | 6 | -2.001535 | 2.489339 | -7.038366 |
| 1 | 0.817248 | 0.965223 | -6.763339 | 1 | -1.037043 | -0.146908 | -7.784098 |
| 1 | -1.228256 | 3.873579 | -6.554956 | 1 | -3.071081 | 2.728765 | -7.086797 |
| 1 | 0.424152 | 3.497451 | -7.046066 | 1 | -1.650422 | 2.342813 | -8.062954 |
| 1 | 0.132201 | 4.456037 | -5.586127 | 1 | -1.480838 | 3.358959 | -6.621791 |
| 8 | -0.738625 | 5.219629 | -3.179008 | 8 | -0.537142 | 5.717409 | -2.449048 |
| 1 | -2.737600 | 4.663120 | -3.020761 | 1 | -3.303457 | 3.788295 | -3.281318 |
| 1 | -2.024112 | 4.413063 | -4.600633 | 1 | -3.095623 | 3.446746 | -5.070724 |
| 6 | -0.926077 | 6.538362 | -3.450743 | 6 | -0.690769 | 6.973452 | -2.529310 |
| 6 | 0.213926 | 7.369474 | -3.000291 | 6 | 0.539409 | 7.830164 | -2.236077 |
| 8 | -1.939103 | 6.948804 | -3.997751 | 8 | -1.767798 | 7.579717 | -2.784464 |
| 6 | 0.397815 | 8.743463 | -3.459581 | 6 | 0.966652 | 8.929447 | -3.082423 |
| 6 | 1.119661 | 6.817852 | -2.113773 | 6 | 1.262587 | 7.527076 | -1.111031 |
| 7 | 2.175907 | 7.467024 | -1.583773 | 7 | 2.336790 | 8.231424 | -0.650542 |
| 1 | 1.001827 | 5.794227 | -1.786159 | 1 | 0.986366 | 6.673985 | -0.503462 |
| 6 | 1.587281 | 9.415107 | -2.897352 | 6 | 2.158376 | 9.652814 | -2.598810 |
| 8 | -0.339746 | 9.330680 | -4.273250 | 8 | 0.422884 | 9.244784 | -4.166774 |
| 6 | 2.456649 | 8.784500 | -1.981882 | 6 | 2.826667 | 9.299903 | -1.402896 |
| 6 | 3.079854 | 6.758896 | -0.685163 | 6 | 3.032174 | 7.783638 | 0.545379 |
| 6 | 2.525766 | 5.830554 | 0.362143 | 6 | 2.242268 | 7.327885 | 1.743837 |
| 6 | 3.147098 | 7.142788 | 0.772370 | 6 | 3.003113 | 8.629416 | 1.795849 |
| 1 | 4.007745 | 6.468315 | -1.172347 | 1 | 3.955562 | 7.250717 | 0.326182 |
| 1 | 2.471943 | 7.922851 | 1.112721 | 1 | 2.419280 | 9.544970 | 1.762145 |
| 1 | 4.126098 | 7.145105 | 1.243000 | 1 | 3.916171 | 8.688865 | 2.381612 |
| 1 | 3.072612 | 4.908548 | 0.535182 | 1 | 2.625868 | 6.469301 | 2.287013 |
| 1 | 1.448106 | 5.741456 | 0.460929 | 1 | 1.158857 | 7.388422 | 1.701910 |
| 6 | 1.878539 | 10.737872 | -3.282900 | 6 | 2.664155 | 10.738606 | -3.343464 |
| 6 | 2.981364 | 11.379001 | -2.782461 | 6 | 3.773955 | 11.417744 | -2.916707 |
| 6 | 3.895049 | 10.766164 | -1.885130 | 6 | 4.491505 | 11.070162 | -1.741736 |
| 6 | 3.597141 | 9.452406 | -1.500706 | 6 | 3.986343 | 9.996683 | -1.002037 |
| 1 | 1.208552 | 11.251846 | -3.963490 | 1 | 2.150665 | 11.044893 | -4.248469 |
| 1 | 4.267294 | 8.935771 | -0.829781 | 1 | 4.503492 | 9.683703 | -0.106853 |
| 9 | 3.186974 | 12.681489 | -3.136234 | 9 | 4.188641 | 12.503547 | -3.638546 |
| 7 | 4.987841 | 11.480709 | -1.381756 | 7 | 5.606975 | 11.822786 | -1.342423 |
| 6 | 5.945120 | 12.033036 | -2.362528 | 6 | 6.736915 | 11.893761 | -2.290932 |
| 6 | 5.682581 | 10.932081 | -0.213322 | 6 | 6.084190 | 11.649057 | 0.031991 |
| 6 | 6.808069 | 13.117731 | -1.723645 | 6 | 7.674006 | 13.042675 | -1.928749 |
| 1 | 5.404814 | 12.460877 | -3.206019 | 1 | 6.360766 | 12.049152 | -3.301645 |
| 1 | 6.590230 | 11.222494 | -2.741419 | 1 | 7.294838 | 10.941845 | -2.273305 |
| 6 | 6.542987 | 12.009438 | 0.450445 | 6 | 7.013266 | 12.799853 | 0.424081 |
| 1 | 6.319380 | 10.074702 | -0.491575 | 1 | 6.618530 | 10.690759 | 0.154616 |
| 1 | 4.942414 | 10.589558 | 0.514744 | 1 | 5.227399 | 11.653677 | 0.711188 |
| 7 | 7.461486 | 12.606443 | -0.519825 | 7 | 8.109586 | 12.927790 | -0.537555 |
| 1 | 7.112937 | 11.564775 | 1.264031 | 1 | 7.420752 | 12.613333 | 1.415966 |
| 1 | 5.891162 | 12.791539 | 0.867844 | 1 | 6.439879 | 13.738588 | 0.453277 |

| | | | | | | | |
|---|---|---|---|---|---|---|---|
| 1 | 6.180948 | 13.984182 | -1.464375 | 1 | 7.155629 | 14.002410 | -2.075544 |
| 1 | 7.574553 | 13.443489 | -2.426862 | 1 | 8.552750 | 13.021958 | -2.573194 |
| 6 | 8.746288 | 12.984200 | -0.236364 | 6 | 9.394889 | 13.259139 | -0.203990 |
| 8 | 9.455947 | 13.619098 | -1.018015 | 8 | 10.270421 | 13.507688 | -1.034586 |
| 8 | 9.131106 | 12.558599 | 0.986478 | 8 | 9.576876 | 13.259670 | 1.134702 |
| 6 | 10.499374 | 12.779918 | 1.510288 | 6 | 10.896969 | 13.515074 | 1.756790 |
| 6 | 10.433965 | 12.111151 | 2.886354 | 6 | 10.583540 | 13.388409 | 3.250440 |
| 6 | 10.773049 | 14.280277 | 1.653341 | 6 | 11.372177 | 14.933143 | 1.425275 |
| 6 | 11.526159 | 12.076103 | 0.617849 | 6 | 11.900299 | 12.441447 | 1.324494 |
| 1 | 11.265421 | 11.018793 | 0.493875 | 1 | 11.501710 | 11.440735 | 1.526904 |
| 1 | 12.512440 | 12.128330 | 1.092934 | 1 | 12.825577 | 12.560314 | 1.899788 |
| 1 | 11.589792 | 12.543978 | -0.366526 | 1 | 12.139591 | 12.520123 | 0.262157 |
| 1 | 9.984778 | 14.758272 | 2.246136 | 1 | 10.603446 | 15.666924 | 1.693351 |
| 1 | 10.832640 | 14.771741 | 0.680379 | 1 | 11.609498 | 15.039348 | 0.365067 |
| 1 | 11.725229 | 14.424641 | 2.176593 | 1 | 12.272491 | 15.156901 | 2.008939 |
| 1 | 9.669791 | 12.583961 | 3.512806 | 1 | 9.833444 | 14.126619 | 3.554368 |
| 1 | 11.401392 | 12.205799 | 3.390787 | 1 | 11.493143 | 13.558913 | 3.835966 |
| 1 | 10.197007 | 11.045984 | 2.790373 | 1 | 10.203985 | 12.387921 | 3.484838 |
| 9 | 0.224435 | -0.532205 | -1.832477 | 9 | -0.259045 | -1.507107 | -2.855908 |
| 9 | -1.841207 | -1.006170 | -2.706639 | 9 | -2.509860 | -1.908610 | -3.051727 |
| 6 | -1.852031 | -0.438922 | 0.467385 | 6 | -1.346020 | -1.191874 | -0.039470 |
| 1 | -2.354167 | -1.232340 | -0.099526 | 1 | -1.869401 | -2.054970 | -0.463554 |
| 1 | -0.802269 | -0.740099 | 0.572558 | 1 | -0.270688 | -1.376820 | -0.163342 |
| 1 | -2.300042 | -0.380620 | 1.462514 | 1 | -1.565290 | -1.120444 | 1.027604 |
| 6 | 0.577357 | -1.257481 | -4.825776 | 6 | -0.652838 | -2.268596 | -5.766236 |
| 1 | 1.303654 | -1.457523 | -4.028214 | 1 | 0.357813 | -2.348690 | -5.343993 |
| 1 | -0.269515 | -1.934741 | -4.660412 | 1 | -1.293899 | -2.944473 | -5.191145 |
| 1 | 1.037831 | -1.499399 | -5.786971 | 1 | -0.620299 | -2.593061 | -6.808100 |
| Min $S_0$ (explicit water) | | | | Min $S_1$ (explicit water) | | | |
| 6 | -1.577312 | 0.872572 | -0.174316 | 6 | -1.494839 | 0.777540 | -0.253079 |
| 6 | -2.125918 | 2.044323 | 0.383440 | 6 | -1.941193 | 1.957863 | 0.378883 |
| 6 | -2.198233 | 3.019518 | -0.605948 | 6 | -2.009920 | 2.970965 | -0.559520 |
| 7 | -1.306156 | 1.095435 | -1.481066 | 7 | -1.284484 | 1.040634 | -1.565704 |
| 6 | -1.673510 | 2.413005 | -1.800569 | 6 | -1.592556 | 2.379886 | -1.811300 |
| 1 | -2.434266 | 2.156078 | 1.415423 | 1 | -2.180788 | 2.040282 | 1.431670 |
| 6 | -2.735793 | 4.402212 | -0.369741 | 6 | -2.434925 | 4.375406 | -0.270432 |
| 1 | -2.947606 | 4.531870 | 0.695744 | 1 | -2.504094 | 4.518750 | 0.811008 |
| 1 | -3.674455 | 4.579539 | -0.909796 | 1 | -3.421142 | 4.607585 | -0.690281 |
| 1 | -2.028744 | 5.184982 | -0.662744 | 1 | -1.727842 | 5.114865 | -0.658363 |
| 5 | -0.691657 | 0.075350 | -2.465327 | 5 | -0.813540 | 0.022498 | -2.610359 |
| 7 | -0.549407 | 0.774577 | -3.835733 | 7 | -0.639056 | 0.775947 | -3.934049 |
| 6 | -1.490185 | 2.899380 | -3.105647 | 6 | -1.465568 | 2.927347 | -3.112962 |
| 6 | -0.932348 | 2.096287 | -4.116926 | 6 | -0.940442 | 2.121590 | -4.154752 |
| 6 | -1.887884 | 4.317885 | -3.430667 | 6 | -1.859089 | 4.338699 | -3.380564 |
| 6 | -0.041785 | 0.219229 | -4.960790 | 6 | -0.169687 | 0.238501 | -5.086003 |
| 6 | -0.633961 | 2.349922 | -5.501474 | 6 | -0.615413 | 2.423452 | -5.531818 |
| 6 | -0.082017 | 1.172264 | -5.996915 | 6 | -0.148340 | 1.242229 | -6.077746 |
| 6 | -0.835088 | 3.584554 | -6.334373 | 6 | -0.719680 | 3.720862 | -6.269396 |
| 1 | 0.264453 | 1.004879 | -7.009054 | 1 | 0.190135 | 1.094991 | -7.095704 |
| 1 | -1.897728 | 3.805739 | -6.495328 | 1 | -1.759200 | 4.005906 | -6.471785 |
| 1 | -0.383938 | 3.434015 | -7.319636 | 1 | -0.217540 | 3.628819 | -7.235994 |
| 1 | -0.372466 | 4.474043 | -5.894012 | 1 | -0.247869 | 4.548905 | -5.730371 |
| 8 | -0.730838 | 5.167370 | -3.170095 | 8 | -0.715629 | 5.208348 | -3.098859 |
| 1 | -2.717036 | 4.658902 | -2.816071 | 1 | -2.685011 | 4.661600 | -2.750611 |
| 1 | -2.177810 | 4.426139 | -4.471842 | 1 | -2.150874 | 4.486121 | -4.415573 |
| 6 | -0.902074 | 6.502039 | -3.415625 | 6 | -0.872373 | 6.521316 | -3.388427 |
| 6 | 0.243825 | 7.312798 | -2.975300 | 6 | 0.257983 | 7.347817 | -2.944428 |
| 8 | -1.920515 | 6.929373 | -3.938190 | 8 | -1.869979 | 6.935581 | -3.951642 |

| | | | | | | | |
|---|---|---|---|---|---|---|---|
| 6 | 0.521110 | 8.617509 | -3.572195 | 6 | 0.551744 | 8.625784 | -3.573820 |
| 6 | 1.047305 | 6.833455 | -1.957254 | 6 | 1.028406 | 6.906537 | -1.894303 |
| 7 | 2.086639 | 7.502205 | -1.423625 | 7 | 2.050042 | 7.588468 | -1.358658 |
| 1 | 0.852792 | 5.867370 | -1.511874 | 1 | 0.824050 | 5.955312 | -1.422836 |
| 6 | 1.694955 | 9.303122 | -3.000496 | 6 | 1.711800 | 9.326329 | -2.999312 |
| 8 | -0.130606 | 9.121042 | -4.504671 | 8 | -0.071663 | 9.099943 | -4.531706 |
| 6 | 2.462038 | 8.750074 | -1.952582 | 6 | 2.443709 | 8.809344 | -1.921511 |
| 6 | 2.878598 | 6.879097 | -0.368580 | 6 | 2.803991 | 7.006936 | -0.262111 |
| 6 | 2.196122 | 6.114768 | 0.733870 | 6 | 2.075735 | 6.319815 | 0.853067 |
| 6 | 2.846625 | 7.443492 | 1.029933 | 6 | 2.743004 | 7.643150 | 1.096105 |
| 1 | 3.826301 | 6.492132 | -0.735488 | 1 | 3.751658 | 6.583359 | -0.582434 |
| 1 | 2.185483 | 8.286177 | 1.211212 | 1 | 2.096928 | 8.508083 | 1.208481 |
| 1 | 3.783465 | 7.468229 | 1.579100 | 1 | 3.661497 | 7.678164 | 1.672913 |
| 1 | 2.680925 | 5.201626 | 1.065614 | 1 | 2.529236 | 5.417858 | 1.249631 |
| 1 | 1.110878 | 6.080934 | 0.751194 | 1 | 0.990750 | 6.309001 | 0.834716 |
| 6 | 2.078760 | 10.557612 | -3.512050 | 6 | 2.110646 | 10.555261 | -3.546840 |
| 6 | 3.173627 | 11.206765 | -3.004743 | 6 | 3.192311 | 11.212573 | -3.038835 |
| 6 | 3.989889 | 10.665870 | -1.976185 | 6 | 3.975037 | 10.704428 | -1.976669 |
| 6 | 3.597101 | 9.421100 | -1.465516 | 6 | 3.567815 | 9.489518 | -1.431740 |
| 1 | 1.486770 | 11.015424 | -4.297034 | 1 | 1.540655 | 10.984973 | -4.362470 |
| 1 | 4.189766 | 8.959462 | -0.689859 | 1 | 4.137546 | 9.053966 | -0.625203 |
| 9 | 3.467757 | 12.448658 | -3.487559 | 9 | 3.512684 | 12.426669 | -3.554872 |
| 7 | 5.080446 | 11.384746 | -1.478559 | 7 | 5.060557 | 11.434247 | -1.489769 |
| 6 | 6.125449 | 11.790206 | -2.441762 | 6 | 6.125745 | 11.752981 | -2.451951 |
| 6 | 5.668564 | 10.945309 | -0.209499 | 6 | 5.616269 | 11.032602 | -0.201575 |
| 6 | 6.989712 | 12.906838 | -1.862837 | 6 | 7.015510 | 12.862818 | -1.916681 |
| 1 | 5.663485 | 12.140855 | -3.363590 | 1 | 5.688226 | 12.072659 | -3.395927 |
| 1 | 6.758329 | 10.919657 | -2.682411 | 1 | 6.732435 | 10.852755 | -2.639813 |
| 6 | 6.530965 | 12.056681 | 0.393027 | 6 | 6.501951 | 12.136963 | 0.361262 |
| 1 | 6.281438 | 10.037789 | -0.345462 | 1 | 6.203249 | 10.103862 | -0.292011 |
| 1 | 4.865816 | 10.716771 | 0.496467 | 1 | 4.799519 | 10.856470 | 0.501926 |
| 7 | 7.538153 | 12.507533 | -0.567890 | 7 | 7.529823 | 12.507096 | -0.602514 |
| 1 | 7.024315 | 11.683804 | 1.288611 | 1 | 6.972168 | 11.792217 | 1.279161 |
| 1 | 5.888757 | 12.904235 | 0.675724 | 1 | 5.885457 | 13.016101 | 0.594235 |
| 1 | 6.384862 | 13.818445 | -1.743013 | 1 | 6.439988 | 13.796574 | -1.847329 |
| 1 | 7.814895 | 13.125496 | -2.540547 | 1 | 7.856300 | 13.023474 | -2.589693 |
| 6 | 8.817379 | 12.864986 | -0.236757 | 6 | 8.814363 | 12.816404 | -0.275184 |
| 8 | 9.605399 | 13.381541 | -1.030355 | 8 | 9.630172 | 13.259832 | -1.077718 |
| 8 | 9.100473 | 12.563702 | 1.049139 | 8 | 9.074590 | 12.562456 | 1.016915 |
| 6 | 10.439794 | 12.789810 | 1.641331 | 6 | 10.407890 | 12.757568 | 1.601528 |
| 6 | 10.252783 | 12.281800 | 3.073854 | 6 | 10.199078 | 12.326982 | 3.048800 |
| 6 | 10.771391 | 14.285307 | 1.639985 | 6 | 10.801389 | 14.229430 | 1.533773 |
| 6 | 11.489964 | 11.951872 | 0.905604 | 6 | 11.420655 | 11.848124 | 0.913877 |
| 1 | 11.188743 | 10.898416 | 0.878051 | 1 | 11.074714 | 10.809610 | 0.932990 |
| 1 | 12.443659 | 12.017400 | 1.441719 | 1 | 12.372821 | 11.898123 | 1.451382 |
| 1 | 11.641180 | 12.304538 | -0.116549 | 1 | 11.588636 | 12.147487 | -0.121264 |
| 1 | 9.969338 | 14.855380 | 2.122642 | 1 | 10.022274 | 14.852875 | 1.984088 |
| 1 | 10.914962 | 14.663820 | 0.626206 | 1 | 10.964382 | 14.552822 | 0.505303 |
| 1 | 11.693811 | 14.450467 | 2.208419 | 1 | 11.726155 | 14.381074 | 2.099323 |
| 1 | 9.470587 | 12.850048 | 3.588850 | 1 | 9.440132 | 12.949724 | 3.531853 |
| 1 | 11.187613 | 12.396064 | 3.632759 | 1 | 11.135315 | 12.428449 | 3.605150 |
| 1 | 9.974951 | 11.222203 | 3.077967 | 1 | 9.877709 | 11.282329 | 3.099343 |
| 8 | 2.156616 | 4.029446 | -3.444461 | 8 | 1.984632 | 3.974771 | -2.740420 |
| 1 | 2.179994 | 3.105246 | -3.740033 | 1 | 2.039942 | 3.015088 | -2.860340 |
| 1 | 1.211317 | 4.261439 | -3.431966 | 1 | 1.067053 | 4.209261 | -2.961665 |
| 8 | -4.499641 | 6.544676 | -5.147497 | 8 | -4.253792 | 6.136222 | -5.248272 |
| 1 | -4.739799 | 7.420078 | -5.488940 | 1 | -4.519784 | 6.860254 | -5.833162 |
| 1 | -3.619641 | 6.663363 | -4.739141 | 1 | -3.437042 | 6.440692 | -4.807152 |
| 9 | 0.574778 | -0.349292 | -2.000120 | 9 | 0.407686 | -0.558137 | -2.212813 |

| | | | | | | | |
|---|---|---|---|---|---|---|---|
| 9 | -1.529260 | -1.058874 | -2.580512 | 9 | -1.760764 | -1.013987 | -2.752747 |
| 6 | -1.315558 | -0.423382 | 0.521047 | 6 | -1.269140 | -0.553910 | 0.360023 |
| 1 | -1.844888 | -1.249840 | 0.034172 | 1 | -1.838717 | -1.332220 | -0.160742 |
| 1 | -0.248058 | -0.671142 | 0.508747 | 1 | -0.213003 | -0.842746 | 0.297325 |
| 1 | -1.646413 | -0.360262 | 1.560697 | 1 | -1.566965 | -0.539126 | 1.409954 |
| 6 | 0.469324 | -1.182255 | -5.040242 | 6 | 0.244993 | -1.179451 | -5.217102 |
| 1 | 1.309295 | -1.337936 | -4.353865 | 1 | 1.098514 | -1.402511 | -4.565613 |
| 1 | -0.308322 | -1.904825 | -4.768943 | 1 | -0.562764 | -1.857927 | -4.919347 |
| 1 | 0.806172 | -1.396819 | -6.057492 | 1 | 0.526034 | -1.394413 | -6.249623 |
| Min $T_1$ (explicit water) | | | | TS $T_1$ (explicit water) | | | |
| 6 | -1.584850 | 0.679536 | -0.276388 | 6 | -1.537996 | 0.597141 | -0.244902 |
| 6 | -2.062422 | 1.845161 | 0.348858 | 6 | -1.921365 | 1.796887 | 0.401517 |
| 6 | -2.109180 | 2.871525 | -0.581993 | 6 | -2.077204 | 2.784768 | -0.549340 |
| 7 | -1.325954 | 0.972675 | -1.608070 | 7 | -1.448500 | 0.838178 | -1.594916 |
| 6 | -1.632628 | 2.294413 | -1.843462 | 6 | -1.776505 | 2.152747 | -1.832239 |
| 1 | -2.341233 | 1.916231 | 1.392788 | 1 | -2.058294 | 1.904965 | 1.469678 |
| 6 | -2.566599 | 4.267723 | -0.289759 | 6 | -2.464519 | 4.200739 | -0.260661 |
| 1 | -2.701709 | 4.388935 | 0.789295 | 1 | -2.475429 | 4.363985 | 0.820530 |
| 1 | -3.529746 | 4.497031 | -0.764829 | 1 | -3.468287 | 4.434822 | -0.638454 |
| 1 | -1.846621 | 5.022712 | -0.623334 | 1 | -1.765974 | 4.915798 | -0.707759 |
| 5 | -0.804281 | -0.029431 | -2.652107 | 5 | -1.079739 | -0.216263 | -2.666808 |
| 7 | -0.620710 | 0.741347 | -3.970575 | 7 | -0.954886 | 0.538417 | -4.012588 |
| 6 | -1.467643 | 2.874618 | -3.134467 | 6 | -1.783451 | 2.690049 | -3.170272 |
| 6 | -0.928242 | 2.068459 | -4.178264 | 6 | -1.283829 | 1.855018 | -4.234640 |
| 6 | -1.853431 | 4.290660 | -3.389508 | 6 | -2.229798 | 3.989618 | -3.426282 |
| 6 | -0.123468 | 0.203512 | -5.148994 | 6 | -0.520414 | -0.013715 | -5.193813 |
| 6 | -0.593208 | 2.389061 | -5.570095 | 6 | -1.024020 | 2.158023 | -5.640871 |
| 6 | -0.106736 | 1.215377 | -6.125270 | 6 | -0.558127 | 0.984184 | -6.197289 |
| 6 | -0.709725 | 3.691933 | -6.301153 | 6 | -1.188564 | 3.451730 | -6.374259 |
| 1 | 0.240888 | 1.086155 | -7.142636 | 1 | -0.258613 | 0.834176 | -7.226426 |
| 1 | -1.754373 | 3.977278 | -6.482479 | 1 | -2.245664 | 3.722044 | -6.493332 |
| 1 | -0.224677 | 3.604946 | -7.278160 | 1 | -0.758081 | 3.361670 | -7.375490 |
| 1 | -0.229936 | 4.518224 | -5.764371 | 1 | -0.691136 | 4.280500 | -5.858944 |
| 8 | -0.704578 | 5.182154 | -3.086376 | 8 | -0.540547 | 5.243218 | -3.243617 |
| 1 | -2.682894 | 4.616542 | -2.766089 | 1 | -2.751899 | 4.549705 | -2.668861 |
| 1 | -2.130205 | 4.460972 | -4.425593 | 1 | -2.404566 | 4.320105 | -4.435794 |
| 6 | -0.875611 | 6.502436 | -3.362061 | 6 | -0.757958 | 6.517721 | -3.299239 |
| 6 | 0.258259 | 7.337517 | -2.920974 | 6 | 0.399088 | 7.385405 | -2.878634 |
| 8 | -1.883023 | 6.924069 | -3.915800 | 8 | -1.839575 | 7.043454 | -3.635506 |
| 6 | 0.569226 | 8.603510 | -3.580283 | 6 | 0.736478 | 8.623817 | -3.563909 |
| 6 | 1.015245 | 6.916437 | -1.845087 | 6 | 1.149421 | 6.973845 | -1.802622 |
| 7 | 2.040204 | 7.609390 | -1.310286 | 7 | 2.178092 | 7.672231 | -1.259314 |
| 1 | 0.795066 | 5.980692 | -1.349718 | 1 | 0.926931 | 6.035901 | -1.310031 |
| 6 | 1.734751 | 9.307472 | -3.013190 | 6 | 1.891986 | 9.340203 | -2.991027 |
| 8 | -0.046036 | 9.061347 | -4.560635 | 8 | 0.151205 | 9.055630 | -4.579178 |
| 6 | 2.456316 | 8.812326 | -1.905655 | 6 | 2.597291 | 8.864102 | -1.862808 |
| 6 | 2.773685 | 7.050836 | -0.180324 | 6 | 2.908583 | 7.116062 | -0.129302 |
| 6 | 2.026235 | 6.389202 | 0.946544 | 6 | 2.157761 | 6.457946 | 0.997755 |
| 6 | 2.698303 | 7.721426 | 1.168796 | 6 | 2.833399 | 7.788346 | 1.219841 |
| 1 | 3.725440 | 6.615432 | -0.475441 | 1 | 3.861215 | 6.677201 | -0.418190 |
| 1 | 2.052748 | 8.590721 | 1.256151 | 1 | 2.190886 | 8.660204 | 1.304678 |
| 1 | 3.611595 | 7.766815 | 1.755042 | 1 | 3.745204 | 7.830985 | 1.808805 |
| 1 | 2.472512 | 5.493547 | 1.367978 | 1 | 2.600267 | 5.562079 | 1.422930 |
| 1 | 0.940611 | 6.382122 | 0.917953 | 1 | 1.072308 | 6.453611 | 0.964976 |
| 6 | 2.159119 | 10.518713 | -3.592862 | 6 | 2.324322 | 10.543210 | -3.584283 |
| 6 | 3.250885 | 11.179696 | -3.094492 | 6 | 3.404537 | 11.216594 | -3.078896 |
| 6 | 4.024139 | 10.692946 | -2.007350 | 6 | 4.159900 | 10.750794 | -1.970326 |
| 6 | 3.589112 | 9.494116 | -1.426797 | 6 | 3.722160 | 9.560476 | -1.378446 |

| | | | | | | | |
|---|---|---|---|---|---|---|---|
| 1 | 1.601697 | 10.933556 | -4.425588 | 1 | 1.779756 | 10.941032 | -4.433779 |
| 1 | 4.147549 | 9.074146 | -0.603467 | 1 | 4.269451 | 9.156567 | -0.539492 |
| 9 | 3.586760 | 12.380483 | -3.649512 | 9 | 3.748133 | 12.410210 | -3.648668 |
| 7 | 5.117868 | 11.421296 | -1.528555 | 7 | 5.242846 | 11.496275 | -1.485574 |
| 6 | 6.199134 | 11.727123 | -2.488798 | 6 | 6.345266 | 11.767933 | -2.431028 |
| 6 | 5.660401 | 11.055842 | -0.216405 | 6 | 5.758730 | 11.160902 | -0.155373 |
| 6 | 7.082797 | 12.856257 | -1.966005 | 6 | 7.223926 | 12.907479 | -1.922735 |
| 1 | 5.771479 | 12.024865 | -3.445211 | 1 | 5.938699 | 12.040934 | -3.404133 |
| 1 | 6.810899 | 10.823805 | -2.650494 | 1 | 6.955793 | 10.857503 | -2.555366 |
| 6 | 6.541318 | 12.181684 | 0.330238 | 6 | 6.632169 | 12.297453 | 0.380618 |
| 1 | 6.249246 | 10.124193 | -0.272145 | 1 | 6.346482 | 10.226844 | -0.176478 |
| 1 | 4.833577 | 10.900053 | 0.481613 | 1 | 4.918056 | 11.023110 | 0.529935 |
| 7 | 7.585826 | 12.533106 | -0.632179 | 7 | 7.697404 | 12.621547 | -0.569302 |
| 1 | 6.999932 | 11.859467 | 1.263158 | 1 | 7.071409 | 11.997715 | 1.330266 |
| 1 | 5.919015 | 13.065740 | 0.534607 | 1 | 6.008793 | 13.188160 | 0.550322 |
| 1 | 6.503705 | 13.791249 | -1.926115 | 1 | 6.648934 | 13.845926 | -1.922630 |
| 1 | 7.931061 | 13.002639 | -2.634579 | 1 | 8.086936 | 13.030572 | -2.577021 |
| 6 | 8.872758 | 12.855996 | -0.296791 | 6 | 8.972016 | 12.969691 | -0.212399 |
| 8 | 9.699278 | 13.281737 | -1.105195 | 8 | 9.813705 | 13.380801 | -1.012759 |
| 8 | 9.115809 | 12.632713 | 1.013165 | 8 | 9.185398 | 12.787862 | 1.109162 |
| 6 | 10.453295 | 12.834336 | 1.618002 | 6 | 10.504032 | 13.027381 | 1.740819 |
| 6 | 10.215093 | 12.435470 | 3.077176 | 6 | 10.235199 | 12.667093 | 3.204724 |
| 6 | 10.858714 | 14.308447 | 1.522871 | 6 | 10.889626 | 14.504311 | 1.612610 |
| 6 | 11.473876 | 11.898046 | 0.963798 | 6 | 11.555114 | 12.088846 | 1.140288 |
| 1 | 11.121790 | 10.860825 | 1.002539 | 1 | 11.216764 | 11.047950 | 1.198113 |
| 1 | 12.420144 | 11.955220 | 1.513824 | 1 | 12.485048 | 12.175001 | 1.713874 |
| 1 | 11.659769 | 12.170969 | -0.076846 | 1 | 11.765725 | 12.336096 | 0.097998 |
| 1 | 10.077749 | 14.948307 | 1.949398 | 1 | 10.089147 | 15.143913 | 2.001716 |
| 1 | 11.038230 | 14.609407 | 0.489082 | 1 | 11.089524 | 14.778195 | 0.575000 |
| 1 | 11.778285 | 14.467100 | 2.097763 | 1 | 11.792623 | 14.693742 | 2.204215 |
| 1 | 9.452051 | 13.074112 | 3.535265 | 1 | 9.451483 | 13.306610 | 3.625106 |
| 1 | 11.143862 | 12.543752 | 3.647235 | 1 | 11.147666 | 12.806261 | 3.794087 |
| 1 | 9.886118 | 11.392959 | 3.147007 | 1 | 9.920185 | 11.622001 | 3.296830 |
| 8 | 2.052837 | 3.891893 | -2.871694 | 8 | 1.927464 | 3.893637 | -3.397791 |
| 1 | 2.018319 | 2.923500 | -2.822652 | 1 | 1.953129 | 3.473924 | -4.271322 |
| 1 | 1.125037 | 4.169534 | -2.981333 | 1 | 1.072396 | 4.380854 | -3.370918 |
| 8 | -4.485253 | 6.429596 | -4.995890 | 8 | -3.965957 | 6.346578 | -5.259670 |
| 1 | -4.771301 | 7.292402 | -5.333933 | 1 | -4.030564 | 7.071989 | -5.899636 |
| 1 | -3.591924 | 6.583244 | -4.627867 | 1 | -3.198769 | 6.577213 | -4.682710 |
| 9 | 0.428099 | -0.585562 | -2.229491 | 9 | 0.141980 | -0.828422 | -2.341776 |
| 9 | -1.727560 | -1.091925 | -2.823392 | 9 | -2.084676 | -1.199925 | -2.749809 |
| 6 | -1.367806 | -0.664754 | 0.322277 | 6 | -1.257249 | -0.723214 | 0.375766 |
| 1 | -1.938632 | -1.435188 | -0.210253 | 1 | -1.815898 | -1.523635 | -0.121906 |
| 1 | -0.312044 | -0.958366 | 0.268487 | 1 | -0.192573 | -0.974374 | 0.288233 |
| 1 | -1.676798 | -0.659852 | 1.370466 | 1 | -1.525258 | -0.703868 | 1.434325 |
| 6 | 0.306189 | -1.214315 | -5.283772 | 6 | -0.086972 | -1.428177 | -5.330653 |
| 1 | 1.139811 | -1.443168 | -4.608257 | 1 | 0.849908 | -1.601076 | -4.785918 |
| 1 | -0.507692 | -1.903735 | -5.028144 | 1 | -0.831021 | -2.114675 | -4.911855 |
| 1 | 0.623993 | -1.410974 | -6.310676 | 1 | 0.072302 | -1.670674 | -6.383590 |
| Products $T_1$ (explicit water) | | | | | | | |
| 6 | -4.741481 | -2.002753 | -1.713004 | | | | |
| 6 | -4.591688 | -1.320346 | -0.471207 | | | | |
| 6 | -3.871577 | -0.169555 | -0.682975 | | | | |
| 7 | -4.102384 | -1.287198 | -2.686271 | | | | |
| 6 | -3.570241 | -0.153904 | -2.115669 | | | | |
| 1 | -4.982535 | -1.674295 | 0.473619 | | | | |
| 6 | -3.472931 | 0.810471 | 0.373472 | | | | |

| | | | |
|---|---|---|---|
| 1 | -3.713829 | 0.403590 | 1.358852 |
| 1 | -4.010025 | 1.761555 | 0.269084 |
| 1 | -2.399232 | 1.027140 | 0.347004 |
| 5 | -4.049504 | -1.662182 | -4.196637 |
| 7 | -3.020205 | -0.696128 | -4.849367 |
| 6 | -2.845947 | 0.808963 | -2.916416 |
| 6 | -2.479887 | 0.422828 | -4.262095 |
| 6 | -2.555363 | 2.068386 | -2.431371 |
| 6 | -2.514989 | -0.805403 | -6.116620 |
| 6 | -1.542583 | 1.038470 | -5.204462 |
| 6 | -1.596385 | 0.259257 | -6.336010 |
| 6 | -0.654781 | 2.220559 | -4.996922 |
| 1 | -1.030987 | 0.403205 | -7.247214 |
| 1 | -1.218252 | 3.158572 | -4.913431 |
| 1 | 0.035548 | 2.310988 | -5.839879 |
| 1 | -0.063231 | 2.117767 | -4.079037 |
| 8 | 1.152807 | 5.350546 | -2.296964 |
| 1 | -2.848772 | 2.363819 | -1.434776 |
| 1 | -2.092624 | 2.837165 | -3.035702 |
| 6 | 0.903443 | 6.534809 | -2.680928 |
| 6 | 1.951911 | 7.595001 | -2.400993 |
| 8 | -0.168842 | 6.905681 | -3.239323 |
| 6 | 2.376139 | 8.574383 | -3.385960 |
| 6 | 2.514999 | 7.604829 | -1.149581 |
| 7 | 3.416113 | 8.522370 | -0.702628 |
| 1 | 2.238634 | 6.850238 | -0.423475 |
| 6 | 3.389852 | 9.530627 | -2.901965 |
| 8 | 1.973038 | 8.610745 | -4.570418 |
| 6 | 3.897825 | 9.494914 | -1.582775 |
| 6 | 3.959304 | 8.401416 | 0.641447 |
| 6 | 3.045440 | 8.066826 | 1.790554 |
| 6 | 3.640990 | 9.448533 | 1.681225 |
| 1 | 4.958072 | 7.969679 | 0.657472 |
| 1 | 2.972603 | 10.252971 | 1.387323 |
| 1 | 4.438823 | 9.739234 | 2.358856 |
| 1 | 3.431117 | 7.382474 | 2.540342 |
| 1 | 1.982037 | 7.968510 | 1.593766 |
| 6 | 3.883341 | 10.521273 | -3.775348 |
| 6 | 4.832556 | 11.411989 | -3.350044 |
| 6 | 5.391888 | 11.384766 | -2.045084 |
| 6 | 4.896621 | 10.404696 | -1.178848 |
| 1 | 3.486775 | 10.583066 | -4.783044 |
| 1 | 5.298632 | 10.333409 | -0.178941 |
| 9 | 5.233692 | 12.394182 | -4.212950 |
| 7 | 6.344085 | 12.340198 | -1.660062 |
| 6 | 7.591274 | 12.394454 | -2.450134 |
| 6 | 6.620315 | 12.482172 | -0.227788 |
| 6 | 8.331884 | 13.705331 | -2.200108 |
| 1 | 7.359052 | 12.315864 | -3.511681 |
| 1 | 8.239688 | 11.545161 | -2.175012 |
| 6 | 7.350318 | 13.798452 | 0.047965 |
| 1 | 7.228267 | 11.641624 | 0.149842 |
| 1 | 5.673673 | 12.494488 | 0.319231 |
| 7 | 8.560684 | 13.897233 | -0.768887 |
| 1 | 7.618364 | 13.848837 | 1.101630 |
| 1 | 6.682665 | 14.642013 | -0.183094 |
| 1 | 7.740192 | 14.545088 | -2.595033 |
| 1 | 9.295168 | 13.690707 | -2.709897 |
| 6 | 9.741533 | 14.439535 | -0.339894 |

| | | | | | | | |
|---|---|---|---|---|---|---|---|
| 8 | 10.704253 | 14.645019 | -1.080926 | | | | |
| 8 | 9.717277 | 14.698142 | 0.985914 | | | | |
| 6 | 10.893994 | 15.228308 | 1.713325 | | | | |
| 6 | 10.372803 | 15.325095 | 3.150070 | | | | |
| 6 | 11.262476 | 16.616360 | 1.180369 | | | | |
| 6 | 12.057849 | 14.236062 | 1.628514 | | | | |
| 1 | 11.739725 | 13.242823 | 1.965477 | | | | |
| 1 | 12.867760 | 14.573318 | 2.285431 | | | | |
| 1 | 12.445706 | 14.158265 | 0.610918 | | | | |
| 1 | 10.389641 | 17.278869 | 1.202672 | | | | |
| 1 | 11.643293 | 16.565924 | 0.158567 | | | | |
| 1 | 12.036813 | 17.054273 | 1.820631 | | | | |
| 1 | 9.511544 | 15.999648 | 3.206555 | | | | |
| 1 | 11.159882 | 15.714213 | 3.804612 | | | | |
| 1 | 10.069919 | 14.340021 | 3.521330 | | | | |
| 8 | 3.570527 | 4.197608 | -1.751056 | | | | |
| 1 | 3.599291 | 3.436320 | -2.350577 | | | | |
| 1 | 2.712292 | 4.656457 | -1.953415 | | | | |
| 8 | -2.103140 | 5.063219 | -3.768697 | | | | |
| 1 | -2.768985 | 5.571239 | -4.257687 | | | | |
| 1 | -1.372418 | 5.712690 | -3.564207 | | | | |
| 9 | -3.618652 | -2.983396 | -4.341755 | | | | |
| 9 | -5.312619 | -1.498790 | -4.781072 | | | | |
| 6 | -5.451946 | -3.280596 | -1.959366 | | | | |
| 1 | -6.093739 | -3.217038 | -2.844074 | | | | |
| 1 | -4.728781 | -4.085741 | -2.145509 | | | | |
| 1 | -6.054285 | -3.549401 | -1.089454 | | | | |
| 6 | -2.888305 | -1.885723 | -7.061150 | | | | |
| 1 | -2.448004 | -2.838989 | -6.740083 | | | | |
| 1 | -3.973307 | -2.030105 | -7.093258 | | | | |
| 1 | -2.521584 | -1.652997 | -8.062703 | | | | |

| **Compound 4** | | | | | | | |
|---|---|---|---|---|---|---|---|
| Min $S_0$ (implicit water) | | | | Min $S_1$ (implicit water) | | | |
| 6 | -1.795776 | -4.258962 | 3.928837 | 6 | -1.684993 | -4.295499 | 3.827375 |
| 6 | -2.453537 | -3.092802 | 4.406820 | 6 | -2.295195 | -3.128451 | 4.355116 |
| 6 | -2.540460 | -1.952551 | 3.621726 | 6 | -2.377956 | -1.974991 | 3.606157 |
| 6 | -1.238820 | -4.195100 | 2.618511 | 6 | -1.170794 | -4.216218 | 2.504412 |
| 6 | -1.336651 | -3.046936 | 1.850750 | 6 | -1.262728 | -3.055173 | 1.771601 |
| 6 | -1.989880 | -1.882814 | 2.321485 | 6 | -1.869014 | -1.886306 | 2.290337 |
| 1 | -2.897105 | -3.079652 | 5.395247 | 1 | -2.704044 | -3.131069 | 5.357412 |
| 1 | -3.052041 | -1.080738 | 4.024805 | 1 | -2.852085 | -1.100291 | 4.044318 |
| 1 | -0.893035 | -3.057388 | 0.858995 | 1 | -0.853285 | -3.052208 | 0.766498 |
| 6 | -2.119006 | -0.657237 | 1.559580 | 6 | -1.988773 | -0.654427 | 1.569816 |
| 7 | -1.700213 | -5.394528 | 4.691573 | 7 | -1.594853 | -5.442614 | 4.554837 |
| 1 | -0.726535 | -5.054232 | 2.201873 | 1 | -0.696349 | -5.078482 | 2.053490 |
| 6 | -2.286943 | -5.433511 | 6.026181 | 6 | -2.136977 | -5.499108 | 5.902139 |
| 6 | -1.019026 | -6.575920 | 4.173779 | 6 | -0.962488 | -6.623290 | 3.990016 |
| 1 | 0.032901 | -6.365011 | 3.939458 | 1 | 0.083795 | -6.430478 | 3.725354 |
| 1 | -1.505035 | -6.957760 | 3.265852 | 1 | -1.488453 | -6.971358 | 3.093028 |
| 1 | -1.046431 | -7.361644 | 4.928867 | 1 | -0.982870 | -7.424149 | 4.727372 |
| 1 | -3.371544 | -5.263012 | 5.995201 | 1 | -3.215179 | -5.299792 | 5.909564 |
| 1 | -1.838221 | -4.679189 | 6.686490 | 1 | -1.646333 | -4.774855 | 6.563279 |
| 1 | -2.110516 | -6.415922 | 6.464423 | 1 | -1.973699 | -6.495399 | 6.309847 |
| 6 | -1.651991 | -0.411043 | 0.300745 | 6 | -1.546602 | -0.401327 | 0.293668 |
| 1 | -2.653121 | 0.140640 | 2.074703 | 1 | -2.484592 | 0.149939 | 2.110122 |
| 6 | -1.822959 | 0.839971 | -0.384428 | 6 | -1.692296 | 0.838486 | -0.366242 |

| | | | | | | | |
|---|---|---|---|---|---|---|---|
| 1 | -1.116904 | -1.186120 | -0.236268 | 1 | -1.050821 | -1.188482 | -0.262471 |
| 6 | -2.467332 | 2.023014 | 0.064957 | 6 | -2.281874 | 2.043862 | 0.099940 |
| 6 | -2.399093 | 2.979884 | -0.933242 | 6 | -2.209604 | 2.994889 | -0.889412 |
| 7 | -1.354264 | 1.070636 | -1.649868 | 7 | -1.250785 | 1.068595 | -1.653667 |
| 6 | -1.689969 | 2.373307 | -2.031837 | 6 | -1.553263 | 2.366406 | -2.014215 |
| 1 | -2.930726 | 2.158809 | 1.033434 | 1 | -2.711048 | 2.192129 | 1.082339 |
| 6 | -2.967107 | 4.364867 | -0.794325 | 6 | -2.711421 | 4.400064 | -0.746892 |
| 1 | -3.311868 | 4.515258 | 0.233436 | 1 | -2.982250 | 4.584869 | 0.296245 |
| 1 | -3.830157 | 4.528896 | -1.451858 | 1 | -3.606718 | 4.588222 | -1.350583 |
| 1 | -2.229101 | 5.143425 | -1.013787 | 1 | -1.957997 | 5.141115 | -1.030491 |
| 5 | -0.582519 | 0.064653 | -2.525402 | 5 | -0.539569 | 0.049922 | -2.541074 |
| 7 | -0.214871 | 0.763803 | -3.848302 | 7 | -0.172848 | 0.741193 | -3.851994 |
| 6 | -1.315806 | 2.865537 | -3.296353 | 6 | -1.211904 | 2.865562 | -3.290106 |
| 6 | -0.567844 | 2.073589 | -4.187768 | 6 | -0.485185 | 2.045136 | -4.180317 |
| 6 | -1.725630 | 4.257411 | -3.703248 | 6 | -1.614405 | 4.244624 | -3.693552 |
| 6 | 0.504727 | 0.213435 | -4.874782 | 6 | 0.528909 | 0.164944 | -4.891740 |
| 6 | -0.023510 | 2.346405 | -5.494223 | 6 | 0.062789 | 2.313096 | -5.491357 |
| 6 | 0.630089 | 1.193525 | -5.894834 | 6 | 0.673748 | 1.152763 | -5.902263 |
| 6 | -0.079995 | 3.598753 | -6.323985 | 6 | 0.036622 | 3.581131 | -6.290201 |
| 6 | 1.006849 | -1.132224 | -4.847697 | 6 | 0.980143 | -1.172877 | -4.874393 |
| 1 | 1.157370 | 1.064807 | -6.831098 | 1 | 1.192675 | 1.016296 | -6.842163 |
| 1 | -1.090305 | 3.803518 | -6.700235 | 1 | -0.959979 | 3.805138 | -6.687679 |
| 1 | 0.571994 | 3.487046 | -7.195906 | 1 | 0.710118 | 3.484787 | -7.146262 |
| 1 | 0.254205 | 4.482368 | -5.770451 | 1 | 0.363347 | 4.446775 | -5.706138 |
| 6 | 1.712681 | -1.701042 | -5.868226 | 6 | 1.663651 | -1.772246 | -5.904749 |
| 1 | 0.796429 | -1.707385 | -3.953070 | 1 | 0.758634 | -1.744959 | -3.980866 |
| 6 | 2.248786 | -3.046009 | -5.922804 | 6 | 2.142279 | -3.121438 | -5.944880 |
| 1 | 1.909226 | -1.099687 | -6.755317 | 1 | 1.873297 | -1.181048 | -6.794295 |
| 6 | 2.109102 | -3.999879 | -4.886456 | 6 | 1.976564 | -4.054916 | -4.894173 |
| 6 | 2.959884 | -3.463904 | -7.070950 | 6 | 2.826678 | -3.583489 | -7.092182 |
| 6 | 2.638927 | -5.275226 | -4.986249 | 6 | 2.453495 | -5.342748 | -4.980368 |
| 6 | 3.356031 | -5.690098 | -6.145943 | 6 | 3.140626 | -5.798337 | -6.138510 |
| 6 | 3.498400 | -4.736653 | -7.190849 | 6 | 3.311142 | -4.869045 | -7.196971 |
| 1 | 1.571774 | -3.740374 | -3.978464 | 1 | 1.459533 | -3.761465 | -3.986153 |
| 1 | 2.497783 | -5.960612 | -4.158953 | 1 | 2.296112 | -6.011684 | -4.143887 |
| 7 | 3.883641 | -6.951855 | -6.246365 | 7 | 3.613942 | -7.071705 | -6.226010 |
| 1 | 3.090903 | -2.763627 | -7.893515 | 1 | 2.975951 | -2.899345 | -7.923694 |
| 1 | 4.031511 | -4.993144 | -8.098634 | 1 | 3.826011 | -5.161129 | -8.103341 |
| 6 | 3.708938 | -7.912812 | -5.162912 | 6 | 3.421926 | -8.004225 | -5.127663 |
| 6 | 4.604264 | -7.352251 | -7.449386 | 6 | 4.309612 | -7.513036 | -7.423307 |
| 1 | 5.482516 | -6.716221 | -7.622792 | 1 | 5.211474 | -6.916833 | -7.605894 |
| 1 | 3.962739 | -7.304412 | -8.339822 | 1 | 3.666082 | -7.447676 | -8.308728 |
| 1 | 4.948114 | -8.380186 | -7.333336 | 1 | 4.609749 | -8.552116 | -7.297889 |
| 1 | 2.647343 | -8.124879 | -4.977177 | 1 | 2.357698 | -8.172028 | -4.923839 |
| 1 | 4.157604 | -7.552223 | -4.227785 | 1 | 3.898640 | -7.645106 | -4.207999 |
| 1 | 4.198911 | -8.847844 | -5.434703 | 1 | 3.871372 | -8.960298 | -5.390942 |
| 8 | -0.675485 | 5.172938 | -3.289331 | 8 | -0.567534 | 5.177876 | -3.307522 |
| 1 | -2.660043 | 4.557158 | -3.234670 | 1 | -2.539403 | 4.556413 | -3.214938 |
| 1 | -1.861458 | 4.340595 | -4.779024 | 1 | -1.763634 | 4.328439 | -4.766798 |
| 6 | -0.883548 | 6.492300 | -3.568173 | 6 | -0.784707 | 6.477083 | -3.601099 |
| 6 | 0.222358 | 7.344477 | -3.081815 | 6 | 0.302912 | 7.349710 | -3.115957 |
| 8 | -1.886537 | 6.871187 | -4.152364 | 8 | -1.782134 | 6.842611 | -4.194394 |
| 6 | 0.290927 | 8.771144 | -3.390075 | 6 | 0.409016 | 8.743256 | -3.516745 |
| 6 | 1.214762 | 6.764097 | -2.312123 | 6 | 1.233026 | 6.817322 | -2.254115 |
| 7 | 2.265227 | 7.420536 | -1.782442 | 7 | 2.253558 | 7.495714 | -1.708034 |
| 1 | 1.179061 | 5.707037 | -2.088773 | 1 | 1.165829 | 5.778212 | -1.963215 |
| 6 | 1.478970 | 9.453543 | -2.837181 | 6 | 1.567227 | 9.450549 | -2.941271 |
| 8 | -0.542253 | 9.398362 | -4.069607 | 8 | -0.365501 | 9.324788 | -4.288098 |
| 6 | 2.446237 | 8.786856 | -2.056074 | 6 | 2.467380 | 8.832763 | -2.062844 |

| | | | | | | | |
|---|---|---|---|---|---|---|---|
| 6 | 3.264981 | 6.682139 | -1.019282 | 6 | 3.189635 | 6.808233 | -0.836917 |
| 6 | 2.832238 | 5.623267 | -0.041075 | 6 | 2.674177 | 5.847678 | 0.191604 |
| 6 | 3.408362 | 6.923328 | 0.463091 | 6 | 3.246990 | 7.168441 | 0.619384 |
| 1 | 4.172082 | 6.497917 | -1.590229 | 1 | 4.123432 | 6.561272 | -1.334216 |
| 1 | 2.716490 | 7.624068 | 0.921786 | 1 | 2.546664 | 7.919510 | 0.971015 |
| 1 | 4.414902 | 6.937933 | 0.871211 | 1 | 4.225059 | 7.197262 | 1.088627 |
| 1 | 3.437814 | 4.723089 | 0.002933 | 1 | 3.252949 | 4.944176 | 0.350364 |
| 1 | 1.769876 | 5.460141 | 0.113553 | 1 | 1.601129 | 5.719100 | 0.287652 |
| 6 | 1.667186 | 10.825042 | -3.094358 | 6 | 1.786493 | 10.794126 | -3.281851 |
| 6 | 2.767750 | 11.477560 | -2.603730 | 6 | 2.858173 | 11.465128 | -2.770547 |
| 6 | 3.779557 | 10.831811 | -1.845335 | 6 | 3.804145 | 10.864873 | -1.908497 |
| 6 | 3.582322 | 9.469113 | -1.585447 | 6 | 3.577285 | 9.534204 | -1.568769 |
| 1 | 0.920859 | 11.364954 | -3.666793 | 1 | 1.085607 | 11.297277 | -3.937835 |
| 1 | 4.328136 | 8.926960 | -1.023264 | 1 | 4.276932 | 9.027022 | -0.922100 |
| 9 | 2.873236 | 12.820551 | -2.825033 | 9 | 3.004632 | 12.780263 | -3.076269 |
| 7 | 4.867981 | 11.555345 | -1.346979 | 7 | 4.867940 | 11.613361 | -1.399652 |
| 6 | 5.711392 | 12.272735 | -2.325358 | 6 | 5.782199 | 12.210959 | -2.383568 |
| 6 | 5.682422 | 10.932722 | -0.299132 | 6 | 5.602493 | 11.053810 | -0.269890 |
| 6 | 6.554340 | 13.339160 | -1.631614 | 6 | 6.623412 | 13.300608 | -1.739236 |
| 1 | 5.082925 | 12.749858 | -3.076114 | 1 | 5.211615 | 12.641766 | -3.204333 |
| 1 | 6.372924 | 11.552960 | -2.836101 | 1 | 6.442269 | 11.430162 | -2.794364 |
| 6 | 6.527438 | 11.988565 | 0.417686 | 6 | 6.441780 | 12.132923 | 0.402257 |
| 1 | 6.344205 | 10.153796 | -0.715385 | 1 | 6.258250 | 10.227069 | -0.589247 |
| 1 | 5.022385 | 10.466608 | 0.437182 | 1 | 4.891709 | 10.665479 | 0.462991 |
| 7 | 7.329565 | 12.747036 | -0.542783 | 7 | 7.314458 | 12.778882 | -0.569334 |
| 1 | 7.184895 | 11.499181 | 1.133762 | 1 | 7.043865 | 11.686632 | 1.190165 |
| 1 | 5.864976 | 12.675637 | 0.965168 | 1 | 5.777872 | 12.883369 | 0.853244 |
| 1 | 5.898248 | 14.127332 | -1.232258 | 1 | 5.977426 | 14.138978 | -1.442990 |
| 1 | 7.242486 | 13.790539 | -2.346193 | 1 | 7.364005 | 13.667874 | -2.448039 |
| 6 | 8.611555 | 13.170253 | -0.317807 | 6 | 8.592651 | 13.170038 | -0.312197 |
| 8 | 9.219943 | 13.927315 | -1.075823 | 8 | 9.266136 | 13.838764 | -1.090340 |
| 8 | 9.117699 | 12.641798 | 0.817662 | 8 | 9.021007 | 12.722536 | 0.878579 |
| 6 | 10.512874 | 12.885500 | 1.253090 | 6 | 10.387662 | 12.960891 | 1.361237 |
| 6 | 10.596617 | 12.074202 | 2.549212 | 6 | 10.385905 | 12.259543 | 2.714674 |
| 6 | 10.725035 | 14.375769 | 1.536805 | 6 | 10.631034 | 14.456422 | 1.534347 |
| 6 | 11.493447 | 12.338607 | 0.211162 | 6 | 11.397415 | 12.308816 | 0.422782 |
| 1 | 11.274044 | 11.287122 | -0.006807 | 1 | 11.156074 | 11.250745 | 0.278741 |
| 1 | 12.512491 | 12.396411 | 0.610371 | 1 | 12.395212 | 12.371497 | 0.868236 |
| 1 | 11.451906 | 12.909870 | -0.718351 | 1 | 11.418110 | 12.804034 | -0.548595 |
| 1 | 9.970366 | 14.742450 | 2.241981 | 1 | 9.851342 | 14.899023 | 2.162592 |
| 1 | 10.674044 | 14.969442 | 0.622156 | 1 | 10.648360 | 14.972267 | 0.573806 |
| 1 | 11.711935 | 14.518709 | 1.991671 | 1 | 11.594336 | 14.609007 | 2.031174 |
| 1 | 9.865211 | 12.434023 | 3.281013 | 1 | 9.633148 | 12.697871 | 3.376760 |
| 1 | 11.597048 | 12.172152 | 2.983810 | 1 | 11.366287 | 12.363926 | 3.188278 |
| 1 | 10.405485 | 11.012810 | 2.357194 | 1 | 10.169771 | 11.193370 | 2.597570 |
| 9 | 0.593943 | -0.365949 | -1.855750 | 9 | 0.627451 | -0.439041 | -1.896314 |
| 9 | -1.380050 | -1.083477 | -2.778121 | 9 | -1.373594 | -1.073875 | -2.782113 |
| Min $T_1$ (implicit water) | | | | TS $T_1$ (implicit water) | | | |
| 6 | -1.796701 | -4.347280 | 3.837959 | 6 | -1.234142 | -4.286660 | 3.926734 |
| 6 | -2.440841 | -3.187587 | 4.355528 | 6 | -1.751383 | -3.075979 | 4.475127 |
| 6 | -2.525553 | -2.027377 | 3.606304 | 6 | -1.889135 | -1.946791 | 3.692203 |
| 6 | -1.250709 | -4.251849 | 2.523571 | 6 | -0.871877 | -4.273342 | 2.544430 |
| 6 | -1.344800 | -3.084632 | 1.789131 | 6 | -1.015815 | -3.135761 | 1.778008 |
| 6 | -1.985335 | -1.923047 | 2.297826 | 6 | -1.529059 | -1.924371 | 2.317959 |
| 1 | -2.874388 | -3.199565 | 5.348351 | 1 | -2.043392 | -3.028151 | 5.517037 |
| 1 | -3.025370 | -1.162658 | 4.037782 | 1 | -2.287235 | -1.041295 | 4.144572 |
| 1 | -0.910151 | -3.072504 | 0.793852 | 1 | -0.724279 | -3.183794 | 0.733184 |
| 6 | -2.110640 | -0.685229 | 1.578080 | 6 | -1.696244 | -0.714221 | 1.569126 |

| | | | | | | | |
|---|---|---|---|---|---|---|---|
| 7 | -1.704619 | -5.502339 | 4.567268 | 7 | -1.090614 | -5.409761 | 4.686863 |
| 1 | -0.749972 | -5.103834 | 2.079333 | 1 | -0.475401 | -5.166687 | 2.077566 |
| 6 | -2.279535 | -5.575198 | 5.906925 | 6 | -1.466499 | -5.399564 | 6.099423 |
| 6 | -1.035700 | -6.675528 | 4.012931 | 6 | -0.559781 | -6.640017 | 4.101919 |
| 1 | 0.014715 | -6.463592 | 3.775042 | 1 | 0.457089 | -6.492413 | 3.717717 |
| 1 | -1.533671 | -7.028902 | 3.100546 | 1 | -1.195120 | -6.998614 | 3.282657 |
| 1 | -1.061792 | -7.479720 | 4.747987 | 1 | -0.525670 | -7.411701 | 4.870168 |
| 1 | -3.361857 | -5.392344 | 5.889429 | 1 | -2.532580 | -5.173767 | 6.226504 |
| 1 | -1.815532 | -4.845556 | 6.583444 | 1 | -0.882689 | -4.659930 | 6.661188 |
| 1 | -2.109625 | -6.572066 | 6.313260 | 1 | -1.272417 | -6.383190 | 6.525661 |
| 6 | -1.644184 | -0.417034 | 0.303261 | 6 | -1.391349 | -0.515813 | 0.236129 |
| 1 | -2.630247 | 0.109079 | 2.111952 | 1 | -2.105687 | 0.123794 | 2.130706 |
| 6 | -1.792006 | 0.820558 | -0.359277 | 6 | -1.580272 | 0.708548 | -0.444874 |
| 1 | -1.126183 | -1.198271 | -0.242201 | 1 | -0.979462 | -1.335784 | -0.341061 |
| 6 | -2.407936 | 2.021414 | 0.092914 | 6 | -2.095954 | 1.943392 | 0.037737 |
| 6 | -2.325197 | 2.978326 | -0.895493 | 6 | -2.107545 | 2.861561 | -0.993336 |
| 7 | -1.320205 | 1.063016 | -1.652770 | 7 | -1.269089 | 0.889969 | -1.789349 |
| 6 | -1.629817 | 2.355740 | -2.014839 | 6 | -1.581741 | 2.174762 | -2.153114 |
| 1 | -2.862279 | 2.162631 | 1.065465 | 1 | -2.421293 | 2.133853 | 1.052202 |
| 6 | -2.855219 | 4.377750 | -0.759200 | 6 | -2.575741 | 4.281388 | -0.854100 |
| 1 | -3.171588 | 4.547472 | 0.275081 | 1 | -2.787194 | 4.494382 | 0.198401 |
| 1 | -3.729224 | 4.560085 | -1.397775 | 1 | -3.499320 | 4.470850 | -1.416335 |
| 1 | -2.102959 | 5.135328 | -1.004911 | 1 | -1.824671 | 5.000099 | -1.199341 |
| 5 | -0.576321 | 0.049856 | -2.531253 | 5 | -0.671163 | -0.186858 | -2.714434 |
| 7 | -0.196110 | 0.751017 | -3.840945 | 7 | -0.403604 | 0.459922 | -4.086365 |
| 6 | -1.268061 | 2.869323 | -3.288618 | 6 | -1.371604 | 2.634969 | -3.506239 |
| 6 | -0.515984 | 2.049515 | -4.171553 | 6 | -0.711505 | 1.749274 | -4.437231 |
| 6 | -1.665997 | 4.251531 | -3.690604 | 6 | -1.771897 | 3.913343 | -3.898499 |
| 6 | 0.534621 | 0.175752 | -4.884879 | 6 | 0.204295 | -0.173172 | -5.166690 |
| 6 | 0.048982 | 2.327663 | -5.485861 | 6 | -0.269798 | 1.969809 | -5.797173 |
| 6 | 0.679016 | 1.171138 | -5.892067 | 6 | 0.285625 | 0.778821 | -6.220729 |
| 6 | 0.016355 | 3.596455 | -6.290211 | 6 | -0.344609 | 3.213004 | -6.636077 |
| 6 | 1.001103 | -1.156248 | -4.864676 | 6 | 0.625332 | -1.522325 | -5.136998 |
| 1 | 1.209603 | 1.041969 | -6.826966 | 1 | 0.720048 | 0.600222 | -7.195747 |
| 1 | -0.983906 | 3.818726 | -6.683920 | 1 | -1.375640 | 3.458852 | -6.922192 |
| 1 | 0.686269 | 3.498633 | -7.150636 | 1 | 0.221477 | 3.063707 | -7.560848 |
| 1 | 0.343997 | 4.466439 | -5.710626 | 1 | 0.072539 | 4.084230 | -6.119589 |
| 6 | 1.711220 | -1.755444 | -5.890045 | 6 | 1.220763 | -2.170355 | -6.202239 |
| 1 | 0.774333 | -1.733887 | -3.975223 | 1 | 0.460529 | -2.063814 | -4.212412 |
| 6 | 2.204708 | -3.105048 | -5.920062 | 6 | 1.668135 | -3.531141 | -6.238438 |
| 1 | 1.929334 | -1.163541 | -6.777737 | 1 | 1.376918 | -1.607520 | -7.120996 |
| 6 | 2.032120 | -4.044609 | -4.868514 | 6 | 1.557040 | -4.439363 | -5.149778 |
| 6 | 2.914261 | -3.563960 | -7.060556 | 6 | 2.265613 | -4.035570 | -7.424722 |
| 6 | 2.525472 | -5.333362 | -4.947745 | 6 | 2.004248 | -5.741139 | -5.236709 |
| 6 | 3.237659 | -5.785591 | -6.098060 | 6 | 2.604953 | -6.240290 | -6.433437 |
| 6 | 3.414727 | -4.850360 | -7.157229 | 6 | 2.719631 | -5.335521 | -7.529897 |
| 1 | 1.497825 | -3.757817 | -3.967414 | 1 | 1.109345 | -4.114328 | -4.215462 |
| 1 | 2.360463 | -6.002975 | -4.112052 | 1 | 1.892681 | -6.386868 | -4.374288 |
| 7 | 3.728169 | -7.061319 | -6.178080 | 7 | 3.048726 | -7.526822 | -6.520882 |
| 1 | 3.070331 | -2.879276 | -7.891495 | 1 | 2.369354 | -3.373913 | -8.281665 |
| 1 | 3.946277 | -5.136431 | -8.056982 | 1 | 3.165214 | -5.659445 | -8.462502 |
| 6 | 3.525128 | -8.001802 | -5.080207 | 6 | 2.910676 | -8.439016 | -5.386776 |
| 6 | 4.445734 | -7.500901 | -7.370710 | 6 | 3.664688 | -8.014454 | -7.753559 |
| 1 | 5.343755 | -6.894027 | -7.544459 | 1 | 4.558594 | -7.432110 | -8.008617 |
| 1 | 3.811093 | -7.445010 | -8.264883 | 1 | 2.963483 | -7.966145 | -8.596077 |
| 1 | 4.756134 | -8.537082 | -7.237585 | 1 | 3.962198 | -9.053330 | -7.615117 |
| 1 | 2.457770 | -8.182028 | -4.896876 | 1 | 1.858039 | -8.572213 | -5.108222 |
| 1 | 3.980224 | -7.637111 | -4.150340 | 1 | 3.461232 | -8.073221 | -4.511202 |
| 1 | 3.991647 | -8.952809 | -5.336356 | 1 | 3.315998 | -9.411733 | -5.663093 |

| | | | | | | | |
|---|---|---|---|---|---|---|---|
| 8 | -0.614671 | 5.198865 | -3.285295 | 8 | -0.157574 | 5.209592 | -3.548779 |
| 1 | -2.594876 | 4.567168 | -3.221416 | 1 | -2.411177 | 4.508842 | -3.269885 |
| 1 | -1.799369 | 4.346850 | -4.765716 | 1 | -1.783234 | 4.189887 | -4.938465 |
| 6 | -0.838731 | 6.509571 | -3.560585 | 6 | -0.425563 | 6.464924 | -3.679628 |
| 6 | 0.260045 | 7.379217 | -3.077237 | 6 | 0.679516 | 7.393576 | -3.214083 |
| 8 | -1.847899 | 6.886107 | -4.139866 | 8 | -1.496441 | 6.939616 | -4.109444 |
| 6 | 0.327060 | 8.801531 | -3.403894 | 6 | 1.034328 | 8.617022 | -3.914350 |
| 6 | 1.247306 | 6.814268 | -2.290800 | 6 | 1.362112 | 7.042340 | -2.074737 |
| 7 | 2.290965 | 7.481522 | -1.758695 | 7 | 2.324744 | 7.792856 | -1.475537 |
| 1 | 1.212685 | 5.759927 | -2.054549 | 1 | 1.136432 | 6.107701 | -1.577304 |
| 6 | 1.507344 | 9.496103 | -2.848379 | 6 | 2.113241 | 9.396150 | -3.275290 |
| 8 | -0.499940 | 9.418182 | -4.101314 | 8 | 0.524955 | 8.995247 | -4.992123 |
| 6 | 2.469643 | 8.844023 | -2.048962 | 6 | 2.743826 | 8.982214 | -2.079746 |
| 6 | 3.285984 | 6.756446 | -0.977410 | 6 | 2.992675 | 7.290546 | -0.284361 |
| 6 | 2.847471 | 5.710684 | 0.012312 | 6 | 2.189299 | 6.624111 | 0.800752 |
| 6 | 3.417290 | 7.018801 | 0.502561 | 6 | 2.793861 | 7.985220 | 1.041082 |
| 1 | 4.198252 | 6.565786 | -1.538039 | 1 | 3.980959 | 6.885756 | -0.492813 |
| 1 | 2.720749 | 7.724786 | 0.945893 | 1 | 2.113203 | 8.831744 | 1.060355 |
| 1 | 4.420555 | 7.040871 | 0.918391 | 1 | 3.658094 | 8.076114 | 1.693049 |
| 1 | 3.454225 | 4.812333 | 0.074161 | 1 | 2.635048 | 5.755969 | 1.277119 |
| 1 | 1.784120 | 5.547858 | 0.160316 | 1 | 1.110326 | 6.573569 | 0.689526 |
| 6 | 1.693063 | 10.864865 | -3.122063 | 6 | 2.544772 | 10.599603 | -3.869100 |
| 6 | 2.786264 | 11.528393 | -2.629783 | 6 | 3.551631 | 11.333023 | -3.299842 |
| 6 | 3.793330 | 10.896984 | -1.853257 | 6 | 4.232282 | 10.931265 | -2.120311 |
| 6 | 3.598829 | 9.537309 | -1.577008 | 6 | 3.797965 | 9.739683 | -1.529831 |
| 1 | 0.950335 | 11.394043 | -3.709026 | 1 | 2.055632 | 10.949853 | -4.771626 |
| 1 | 4.341125 | 9.005551 | -1.000427 | 1 | 4.291465 | 9.383258 | -0.637633 |
| 9 | 2.888697 | 12.868914 | -2.868525 | 9 | 3.891470 | 12.525234 | -3.876749 |
| 7 | 4.874706 | 11.631541 | -1.354444 | 7 | 5.239634 | 11.738027 | -1.571517 |
| 6 | 5.724353 | 12.337741 | -2.335609 | 6 | 6.405731 | 12.027628 | -2.431098 |
| 6 | 5.682583 | 11.023973 | -0.292756 | 6 | 5.655623 | 11.460652 | -0.193918 |
| 6 | 6.560427 | 13.414452 | -1.649397 | 6 | 7.195458 | 13.215204 | -1.887588 |
| 1 | 5.100658 | 12.804245 | -3.096951 | 1 | 6.070887 | 12.257653 | -3.441982 |
| 1 | 6.390903 | 11.612738 | -2.832311 | 1 | 7.059002 | 11.139700 | -2.477796 |
| 6 | 6.520693 | 12.090214 | 0.416649 | 6 | 6.437286 | 12.645923 | 0.376861 |
| 1 | 6.348794 | 10.240946 | -0.694115 | 1 | 6.278682 | 10.551137 | -0.137588 |
| 1 | 5.018009 | 10.566022 | 0.444618 | 1 | 4.766698 | 11.307021 | 0.423933 |
| 7 | 7.328685 | 12.837568 | -0.547575 | 7 | 7.565092 | 12.985950 | -0.491739 |
| 1 | 7.173585 | 11.611147 | 1.143788 | 1 | 6.806827 | 12.391208 | 1.368407 |
| 1 | 5.853235 | 12.783284 | 0.950314 | 1 | 5.768740 | 13.515291 | 0.466302 |
| 1 | 5.900132 | 14.206452 | -1.264804 | 1 | 6.587072 | 14.129198 | -1.963007 |
| 1 | 7.252984 | 13.858007 | -2.364615 | 1 | 8.104588 | 13.355533 | -2.472323 |
| 6 | 8.609404 | 13.263164 | -0.320361 | 6 | 8.794618 | 13.385400 | -0.043264 |
| 8 | 9.222481 | 14.010779 | -1.083990 | 8 | 9.686359 | 13.801987 | -0.784520 |
| 8 | 9.108790 | 12.748517 | 0.824458 | 8 | 8.904202 | 13.247405 | 1.296229 |
| 6 | 10.501538 | 12.996871 | 1.264901 | 6 | 10.158268 | 13.548289 | 2.025477 |
| 6 | 10.577723 | 12.200443 | 2.570677 | 6 | 9.779130 | 13.225407 | 3.473641 |
| 6 | 10.712535 | 14.490255 | 1.532606 | 6 | 10.511133 | 15.031795 | 1.879783 |
| 6 | 11.488005 | 12.437898 | 0.235022 | 6 | 11.282180 | 12.625204 | 1.544902 |
| 1 | 11.269435 | 11.384110 | 0.027589 | 1 | 10.972163 | 11.576179 | 1.613451 |
| 1 | 12.504647 | 12.499806 | 0.639707 | 1 | 12.159992 | 12.761696 | 2.186819 |
| 1 | 11.452289 | 12.998737 | -0.701059 | 1 | 11.568362 | 12.844092 | 0.514298 |
| 1 | 9.953725 | 14.865377 | 2.228838 | 1 | 9.661933 | 15.658113 | 2.176207 |
| 1 | 10.667403 | 15.073242 | 0.610799 | 1 | 10.792746 | 15.278287 | 0.854328 |
| 1 | 11.696705 | 14.638179 | 1.991774 | 1 | 11.353401 | 15.268309 | 2.540017 |
| 1 | 9.842532 | 12.568941 | 3.294322 | 1 | 8.946226 | 13.853954 | 3.807015 |
| 1 | 11.575833 | 12.302830 | 3.009563 | 1 | 10.635566 | 13.409412 | 4.130886 |
| 1 | 10.387044 | 11.137029 | 2.389715 | 1 | 9.485900 | 12.174760 | 3.574144 |
| 9 | 0.591402 | -0.423453 | -1.864168 | 9 | -1.567827 | -1.277247 | -2.847822 |

| | |
|---|---|
| 9 -1.396164 -1.087613 -2.789835 | 9 0.538467 -0.682688 -2.165984 |
| Products $T_1$ (implicit water) | |
| 6 -3.939495 -9.053435 5.596117<br>6 -4.503076 -7.834796 6.086632<br>6 -4.555674 -6.713242 5.289790<br>6 -3.439383 -9.052632 4.253910<br>6 -3.499767 -7.922523 3.472448<br>6 -4.059588 -6.703359 3.954723<br>1 -4.896650 -7.780737 7.093826<br>1 -4.990406 -5.801947 5.693004<br>1 -3.106058 -7.976393 2.462447<br>6 -4.146305 -5.502987 3.191324<br>7 -3.879666 -10.167420 6.369497<br>1 -3.002769 -9.951310 3.836499<br>6 -4.388795 -10.148403 7.742541<br>6 -3.304038 -11.409321 5.848246<br>1 -2.250777 -11.273207 5.576575<br>1 -3.854570 -11.760073 4.967853<br>1 -3.363821 -12.176752 6.618519<br>1 -5.460791 -9.920014 7.762796<br>1 -3.857117 -9.406751 8.349787<br>1 -4.237836 -11.129885 8.189357<br>6 -3.710451 -5.314564 1.890646<br>1 -4.604605 -4.659016 3.703217<br>6 -3.825441 -4.090903 1.196004<br>1 -3.249778 -6.139225 1.359283<br>6 -4.377946 -2.852731 1.629624<br>6 -4.250531 -1.924737 0.614066<br>7 -3.369955 -3.907738 -0.102661<br>6 -3.601575 -2.609889 -0.474452<br>1 -4.830654 -2.669761 2.595163<br>6 -4.742767 -0.509035 0.697052<br>1 -5.304078 -0.371043 1.626007<br>1 -5.407753 -0.252940 -0.135994<br>1 -3.920536 0.217312 0.700938<br>5 -2.717428 -4.999716 -0.976982<br>7 -2.476141 -4.403486 -2.380259<br>6 -3.201384 -2.129551 -1.791493<br>6 -2.706965 -3.106193 -2.754542<br>6 -3.255615 -0.796451 -2.102986<br>6 -1.993992 -5.107840 -3.475341<br>6 -2.381730 -2.961800 -4.150511<br>6 -1.946274 -4.202999 -4.573162<br>6 -2.490386 -1.757752 -5.040385<br>6 -1.628332 -6.470239 -3.418159<br>1 -1.626405 -4.448247 -5.577279<br>1 -3.491438 -1.311676 -5.013073<br>1 -2.283105 -2.048312 -6.074636<br>1 -1.770475 -0.975096 -4.770277<br>6 -1.134941 -7.180157 -4.499747<br>1 -1.746356 -6.969942 -2.463772<br>6 -0.740730 -8.549793 -4.512716<br>1 -1.028462 -6.657199 -5.448168<br>6 -0.795697 -9.411613 -3.378336<br>6 -0.254866 -9.120012 -5.724200<br>6 -0.399714 -10.726898 -3.446209<br>6 0.088494 -11.291983 -4.668481<br>6 0.145285 -10.434551 -5.810808 | |

| | | | | |
|---|---|---|---|---|
| 1 | -1.157520 | -9.034347 | -2.427083 | |
| 1 | -0.463340 | -11.336556 | -2.553598 | |
| 7 | 0.481112 | -12.589549 | -4.737134 | |
| 1 | -0.198004 | -8.493729 | -6.611096 | |
| 1 | 0.506124 | -10.811243 | -6.759656 | |
| 6 | 0.420540 | -13.452338 | -3.555082 | |
| 6 | 0.970771 | -13.153266 | -5.996964 | |
| 1 | 1.866808 | -12.625364 | -6.343371 | |
| 1 | 0.203502 | -13.098585 | -6.777889 | |
| 1 | 1.227316 | -14.199824 | -5.839991 | |
| 1 | -0.608562 | -13.551735 | -3.190843 | |
| 1 | 1.046090 | -13.057553 | -2.746319 | |
| 1 | 0.788148 | -14.442227 | -3.821147 | |
| 8 | -2.203003 | 3.845090 | -2.126872 | |
| 1 | -3.591469 | -0.056913 | -1.392632 | |
| 1 | -2.936740 | -0.420800 | -3.062939 | |
| 6 | -2.464786 | 5.005343 | -1.691004 | |
| 6 | -1.280267 | 5.829310 | -1.187969 | |
| 8 | -3.609921 | 5.528429 | -1.593094 | |
| 6 | -1.025980 | 7.194178 | -1.609994 | |
| 6 | -0.426930 | 5.230365 | -0.297674 | |
| 7 | 0.631366 | 5.840560 | 0.312167 | |
| 1 | -0.573011 | 4.193854 | -0.018827 | |
| 6 | 0.149543 | 7.825623 | -0.981790 | |
| 8 | -1.701332 | 7.808956 | -2.468932 | |
| 6 | 0.959609 | 7.150430 | -0.038641 | |
| 6 | 1.477692 | 5.079075 | 1.216512 | |
| 6 | 0.852092 | 4.147503 | 2.220824 | |
| 6 | 1.511758 | 5.421627 | 2.686699 | |
| 1 | 2.412115 | 4.760775 | 0.758011 | |
| 1 | 0.858887 | 6.221321 | 3.025081 | |
| 1 | 2.472766 | 5.369534 | 3.190809 | |
| 1 | 1.354290 | 3.199644 | 2.390435 | |
| 1 | -0.231327 | 4.100852 | 2.277048 | |
| 6 | 0.493384 | 9.150330 | -1.322584 | |
| 6 | 1.585670 | 9.752232 | -0.757420 | |
| 6 | 2.441806 | 9.095090 | 0.165404 | |
| 6 | 2.097049 | 7.783867 | 0.505989 | |
| 1 | -0.131256 | 9.694811 | -2.022604 | |
| 1 | 2.724296 | 7.236015 | 1.193805 | |
| 9 | 1.843135 | 11.058820 | -1.071484 | |
| 7 | 3.532355 | 9.775752 | 0.729930 | |
| 6 | 4.553959 | 10.285058 | -0.207367 | |
| 6 | 4.155760 | 9.186543 | 1.917909 | |
| 6 | 5.438735 | 11.326448 | 0.472334 | |
| 1 | 4.068360 | 10.741591 | -1.069395 | |
| 1 | 5.176983 | 9.447781 | -0.565959 | |
| 6 | 5.035670 | 10.218474 | 2.626570 | |
| 1 | 4.766425 | 8.304583 | 1.656796 | |
| 1 | 3.372907 | 8.868617 | 2.611921 | |
| 7 | 6.018497 | 10.781385 | 1.699184 | |
| 1 | 5.552415 | 9.742688 | 3.458012 | |
| 1 | 4.403253 | 11.026039 | 3.025019 | |
| 1 | 4.841407 | 12.219533 | 0.710970 | |
| 1 | 6.247820 | 11.620209 | -0.196373 | |
| 6 | 7.303650 | 11.106221 | 2.039404 | |
| 8 | 8.070947 | 11.716493 | 1.293051 | |
| 8 | 7.619241 | 10.659189 | 3.274646 | |
| 6 | 8.970098 | 10.827895 | 3.859233 | |

| | | | | | | | |
|---|---|---|---|---|---|---|---|
| 6 | 8.820617 | 10.153180 | 5.225816 | | | | |
| 6 | 9.290223 | 12.316534 | 4.026346 | | | | |
| 6 | 10.010054 | 10.093645 | 3.007407 | | | | |
| 1 | 9.717053 | 9.047535 | 2.862282 | | | | |
| 1 | 10.975052 | 10.106160 | 3.526900 | | | | |
| 1 | 10.135093 | 10.566039 | 2.031132 | | | | |
| 1 | 8.495679 | 12.817914 | 4.590758 | | | | |
| 1 | 9.406697 | 12.812593 | 3.060926 | | | | |
| 1 | 10.224768 | 12.423274 | 4.588956 | | | | |
| 1 | 8.044971 | 10.647508 | 5.820757 | | | | |
| 1 | 9.766805 | 10.212387 | 5.774002 | | | | |
| 1 | 8.553033 | 9.097134 | 5.111262 | | | | |
| 9 | -3.577483 | -6.117802 | -1.071243 | | | | |
| 9 | -1.496352 | -5.424600 | -0.404947 | | | | |
| Min $S_0$ (explicit water) | | | | Min $S_1$ (explicit water) | | | |
| 6 | -1.044450 | -4.176343 | 4.109990 | 6 | -1.040071 | -4.213389 | 3.978642 |
| 6 | -1.584302 | -2.987314 | 4.672672 | 6 | -1.564876 | -3.039388 | 4.578888 |
| 6 | -1.756353 | -1.848910 | 3.899598 | 6 | -1.733227 | -1.886463 | 3.844370 |
| 6 | -0.695760 | -4.137093 | 2.728396 | 6 | -0.701205 | -4.140940 | 2.599491 |
| 6 | -0.874650 | -2.990036 | 1.974255 | 6 | -0.875664 | -2.980024 | 1.882404 |
| 6 | -1.410187 | -1.802884 | 2.529363 | 6 | -1.397975 | -1.804401 | 2.473174 |
| 1 | -1.869876 | -2.955696 | 5.717350 | 1 | -1.840722 | -3.037211 | 5.625621 |
| 1 | -2.173063 | -0.959408 | 4.367997 | 1 | -2.138704 | -1.006736 | 4.337900 |
| 1 | -0.590906 | -3.019610 | 0.925903 | 1 | -0.599987 | -2.982018 | 0.832692 |
| 6 | -1.615279 | -0.577477 | 1.785798 | 6 | -1.597244 | -0.572471 | 1.772401 |
| 7 | -0.866650 | -5.309994 | 4.859816 | 7 | -0.868133 | -5.360072 | 4.690476 |
| 1 | -0.281759 | -5.014522 | 2.245892 | 1 | -0.297171 | -5.008292 | 2.093147 |
| 6 | -1.234296 | -5.321234 | 6.271241 | 6 | -1.214895 | -5.404606 | 6.101405 |
| 6 | -0.318651 | -6.518133 | 4.252768 | 6 | -0.332418 | -6.550146 | 4.049887 |
| 1 | 0.691351 | -6.348211 | 3.856726 | 1 | 0.678181 | -6.377161 | 3.661463 |
| 1 | -0.953739 | -6.882851 | 3.434404 | 1 | -0.970241 | -6.881740 | 3.222253 |
| 1 | -0.258534 | -7.300425 | 5.009491 | 1 | -0.281530 | -7.354816 | 4.781517 |
| 1 | -2.305822 | -5.124395 | 6.411043 | 1 | -2.280504 | -5.200454 | 6.259519 |
| 1 | -0.668724 | -4.571422 | 6.840309 | 1 | -0.632942 | -4.677647 | 6.680077 |
| 1 | -1.012027 | -6.302924 | 6.689654 | 1 | -0.998487 | -6.398443 | 6.489717 |
| 6 | -1.330716 | -0.348659 | 0.469879 | 6 | -1.319184 | -0.323822 | 0.449534 |
| 1 | -2.044481 | 0.237580 | 2.367650 | 1 | -2.011597 | 0.237098 | 2.370373 |
| 6 | -1.566537 | 0.903878 | -0.190659 | 6 | -1.537291 | 0.916947 | -0.187236 |
| 1 | -0.899922 | -1.139622 | -0.133594 | 1 | -0.904119 | -1.115123 | -0.163783 |
| 6 | -2.117707 | 2.101815 | 0.337785 | 6 | -2.061146 | 2.124308 | 0.345999 |
| 6 | -2.162668 | 3.056801 | -0.662565 | 6 | -2.100114 | 3.076772 | -0.643994 |
| 7 | -1.270668 | 1.123062 | -1.509352 | 7 | -1.251367 | 1.146568 | -1.518389 |
| 6 | -1.619661 | 2.434514 | -1.844862 | 6 | -1.580602 | 2.447548 | -1.837356 |
| 1 | -2.448409 | 2.246524 | 1.357931 | 1 | -2.376470 | 2.271996 | 1.370654 |
| 6 | -2.697319 | 4.446515 | -0.455407 | 6 | -2.596842 | 4.476681 | -0.443812 |
| 1 | -2.925055 | 4.593536 | 0.604810 | 1 | -2.779411 | 4.647734 | 0.620507 |
| 1 | -3.626600 | 4.620897 | -1.012335 | 1 | -3.541889 | 4.662854 | -0.966872 |
| 1 | -1.982508 | 5.221910 | -0.749082 | 1 | -1.877386 | 5.229487 | -0.779389 |
| 5 | -0.656170 | 0.095538 | -2.479283 | 5 | -0.666095 | 0.120723 | -2.486078 |
| 7 | -0.486868 | 0.774273 | -3.852580 | 7 | -0.480410 | 0.801358 | -3.840548 |
| 6 | -1.408591 | 2.916014 | -3.150537 | 6 | -1.383243 | 2.946973 | -3.144634 |
| 6 | -0.839390 | 2.095575 | -4.144281 | 6 | -0.808997 | 2.110226 | -4.128703 |
| 6 | -1.789416 | 4.331059 | -3.491309 | 6 | -1.761263 | 4.347772 | -3.480411 |
| 6 | 0.034718 | 0.190677 | -4.976033 | 6 | 0.055198 | 0.206917 | -4.965914 |
| 6 | -0.507564 | 2.339233 | -5.527129 | 6 | -0.450497 | 2.361571 | -5.507299 |
| 6 | 0.026172 | 1.159303 | -6.014839 | 6 | 0.071837 | 1.186988 | -5.993273 |
| 6 | -0.659408 | 3.581912 | -6.360286 | 6 | -0.572357 | 3.620880 | -6.311787 |
| 6 | 0.488484 | -1.170747 | -5.015543 | 6 | 0.483541 | -1.137555 | -5.002212 |

| | | | | | | | |
|---|---|---|---|---|---|---|---|
| 1 | 0.383987 | 1.004096 | -7.024222 | 1 | 0.444211 | 1.035863 | -6.998092 |
| 1 | -1.711148 | 3.834131 | -6.544735 | 1 | -1.614535 | 3.879806 | -6.530855 |
| 1 | -0.190757 | 3.422733 | -7.336308 | 1 | -0.066008 | 3.486669 | -7.271488 |
| 1 | -0.181191 | 4.456178 | -5.905699 | 1 | -0.112321 | 4.481848 | -5.816405 |
| 6 | 0.995507 | -1.768638 | -6.133708 | 6 | 1.009139 | -1.751350 | -6.114098 |
| 1 | 0.409641 | -1.733545 | -4.092287 | 1 | 0.379919 | -1.702259 | -4.082979 |
| 6 | 1.471316 | -3.129976 | -6.263759 | 6 | 1.460154 | -3.106126 | -6.211806 |
| 1 | 1.060058 | -1.178345 | -7.047111 | 1 | 1.101025 | -1.166771 | -7.027522 |
| 6 | 1.477885 | -4.074212 | -5.209041 | 6 | 1.429661 | -4.032911 | -5.142264 |
| 6 | 1.965205 | -3.575994 | -7.511387 | 6 | 1.974618 | -3.581639 | -7.440065 |
| 6 | 1.943456 | -5.366161 | -5.383842 | 6 | 1.875128 | -5.326428 | -5.285426 |
| 6 | 2.442925 | -5.808922 | -6.643352 | 6 | 2.390998 | -5.795619 | -6.524733 |
| 6 | 2.435447 | -4.865846 | -7.707448 | 6 | 2.424805 | -4.873190 | -7.602745 |
| 1 | 1.108347 | -3.793484 | -4.226660 | 1 | 1.045667 | -3.729128 | -4.173733 |
| 1 | 1.920414 | -6.043583 | -4.538519 | 1 | 1.825973 | -5.990085 | -4.431655 |
| 7 | 2.909304 | -7.086211 | -6.817798 | 7 | 2.832441 | -7.074519 | -6.668347 |
| 1 | 1.977482 | -2.884095 | -8.351210 | 1 | 2.016579 | -2.902785 | -8.288095 |
| 1 | 2.799245 | -5.143895 | -8.689435 | 1 | 2.807202 | -5.175431 | -8.569341 |
| 6 | 2.881641 | -8.039238 | -5.713531 | 6 | 2.785434 | -7.999023 | -5.547295 |
| 6 | 3.378864 | -7.523369 | -8.127738 | 6 | 3.344207 | -7.533024 | -7.949297 |
| 1 | 4.228949 | -6.919603 | -8.471497 | 1 | 4.221972 | -6.954604 | -8.260612 |
| 1 | 2.584125 | -7.462599 | -8.883822 | 1 | 2.583208 | -7.458621 | -8.735101 |
| 1 | 3.705867 | -8.561084 | -8.060857 | 1 | 3.639744 | -8.577202 | -7.861158 |
| 1 | 1.856483 | -8.232747 | -5.368949 | 1 | 1.757530 | -8.159626 | -5.200931 |
| 1 | 3.471123 | -7.680981 | -4.859752 | 1 | 3.383362 | -7.636186 | -4.703225 |
| 1 | 3.312048 | -8.983205 | -6.048070 | 1 | 3.191623 | -8.958960 | -5.861670 |
| 8 | -0.644664 | 5.196028 | -3.191892 | 8 | -0.627376 | 5.229835 | -3.177968 |
| 1 | -2.639282 | 4.678653 | -2.909231 | 1 | -2.611417 | 4.700518 | -2.902649 |
| 1 | -2.037963 | 4.441934 | -4.542262 | 1 | -2.002085 | 4.466163 | -4.531571 |
| 6 | -0.793199 | 6.516224 | -3.500774 | 6 | -0.772591 | 6.533742 | -3.492887 |
| 6 | 0.335528 | 7.342663 | -3.039262 | 6 | 0.349163 | 7.366671 | -3.033785 |
| 8 | -1.780324 | 6.930982 | -4.091637 | 8 | -1.754604 | 6.945364 | -4.087224 |
| 6 | 0.662561 | 8.609584 | -3.689527 | 6 | 0.660975 | 8.635749 | -3.671491 |
| 6 | 1.070516 | 6.916188 | -1.949237 | 6 | 1.092511 | 6.937904 | -1.959624 |
| 7 | 2.084134 | 7.606082 | -1.391733 | 7 | 2.100449 | 7.626194 | -1.405004 |
| 1 | 0.838548 | 5.979079 | -1.461829 | 1 | 0.874406 | 5.991975 | -1.483475 |
| 6 | 1.813939 | 9.312387 | -3.093166 | 6 | 1.804378 | 9.344413 | -3.073717 |
| 8 | 0.069647 | 9.069372 | -4.682273 | 8 | 0.065047 | 9.098253 | -4.652628 |
| 6 | 2.510147 | 8.814096 | -1.970928 | 6 | 2.506827 | 8.841619 | -1.969836 |
| 6 | 2.795686 | 7.042121 | -0.250130 | 6 | 2.827333 | 7.056448 | -0.284418 |
| 6 | 2.028040 | 6.366585 | 0.854642 | 6 | 2.072917 | 6.377117 | 0.818145 |
| 6 | 2.687107 | 7.700841 | 1.102509 | 6 | 2.730501 | 7.704383 | 1.066185 |
| 1 | 3.756067 | 6.614818 | -0.528475 | 1 | 3.783951 | 6.632580 | -0.576507 |
| 1 | 2.034397 | 8.565449 | 1.183101 | 1 | 2.079560 | 8.568480 | 1.154283 |
| 1 | 3.587253 | 7.746730 | 1.708696 | 1 | 3.633945 | 7.747025 | 1.665810 |
| 1 | 2.470726 | 5.469964 | 1.277759 | 1 | 2.518680 | 5.479986 | 1.233973 |
| 1 | 0.943326 | 6.353232 | 0.803259 | 1 | 0.988780 | 6.363027 | 0.772852 |
| 6 | 2.248235 | 10.527534 | -3.656810 | 6 | 2.215903 | 10.567835 | -3.624277 |
| 6 | 3.324136 | 11.190673 | -3.127692 | 6 | 3.280564 | 11.234370 | -3.093131 |
| 6 | 4.070269 | 10.703050 | -2.022144 | 6 | 4.032843 | 10.741682 | -2.002093 |
| 6 | 3.626926 | 9.498358 | -1.459806 | 6 | 3.614449 | 9.531311 | -1.455747 |
| 1 | 1.710030 | 10.944325 | -4.501132 | 1 | 1.668249 | 10.986166 | -4.460866 |
| 1 | 4.166001 | 9.077250 | -0.624220 | 1 | 4.162066 | 9.107297 | -0.627964 |
| 9 | 3.668782 | 12.395309 | -3.668444 | 9 | 3.612700 | 12.443275 | -3.614269 |
| 7 | 5.143903 | 11.436345 | -1.508142 | 7 | 5.099338 | 11.482776 | -1.490601 |
| 6 | 6.250427 | 11.760166 | -2.432560 | 6 | 6.193129 | 11.795558 | -2.421721 |
| 6 | 5.650256 | 11.071602 | -0.181615 | 6 | 5.616174 | 11.101790 | -0.180360 |
| 6 | 7.100908 | 12.899813 | -1.878861 | 6 | 7.055952 | 12.920503 | -1.874330 |
| 1 | 5.849548 | 12.055025 | -3.401339 | 1 | 5.784874 | 12.097632 | -3.384415 |

| | | | | | | | |
|---|---|---|---|---|---|---|---|
| 1 | 6.879414 | 10.865843 | -2.577058 | 1 | 6.812498 | 10.897528 | -2.575782 |
| 6 | 6.496425 | 12.207617 | 0.397583 | 6 | 6.473508 | 12.221115 | 0.396490 |
| 1 | 6.253951 | 10.148798 | -0.223520 | 1 | 6.213517 | 10.177148 | -0.240062 |
| 1 | 4.804572 | 10.900436 | 0.489660 | 1 | 4.778709 | 10.927313 | 0.498798 |
| 7 | 7.565284 | 12.580535 | -0.529818 | 7 | 7.528978 | 12.589285 | -0.538162 |
| 1 | 6.930990 | 11.887059 | 1.342575 | 1 | 6.917164 | 11.891982 | 1.333208 |
| 1 | 5.854128 | 13.080621 | 0.587495 | 1 | 5.841699 | 13.097036 | 0.598875 |
| 1 | 6.508129 | 13.826859 | -1.856566 | 1 | 6.471434 | 13.850641 | -1.839344 |
| 1 | 7.968336 | 13.059160 | -2.519388 | 1 | 7.917594 | 13.077396 | -2.521425 |
| 6 | 8.827535 | 12.946556 | -0.146705 | 6 | 8.793199 | 12.934007 | -0.168751 |
| 8 | 9.668693 | 13.400035 | -0.924204 | 8 | 9.627656 | 13.384347 | -0.947850 |
| 8 | 9.029137 | 12.730283 | 1.171283 | 8 | 9.011317 | 12.705487 | 1.135777 |
| 6 | 10.334242 | 12.981373 | 1.826576 | 6 | 10.317273 | 12.941350 | 1.765538 |
| 6 | 10.055428 | 12.574479 | 3.276333 | 6 | 10.066075 | 12.526793 | 3.210755 |
| 6 | 10.688215 | 14.469432 | 1.744402 | 6 | 10.677674 | 14.421264 | 1.689619 |
| 6 | 11.413332 | 12.083535 | 1.213719 | 6 | 11.376051 | 12.046949 | 1.129408 |
| 1 | 11.098818 | 11.033944 | 1.240229 | 1 | 11.054483 | 11.000704 | 1.151996 |
| 1 | 12.334798 | 12.175914 | 1.799881 | 1 | 12.306292 | 12.128028 | 1.700436 |
| 1 | 11.629399 | 12.363264 | 0.180785 | 1 | 11.574939 | 12.335069 | 0.096606 |
| 1 | 9.868188 | 15.080175 | 2.139104 | 1 | 9.867992 | 15.031833 | 2.102069 |
| 1 | 10.896674 | 14.776415 | 0.717801 | 1 | 10.870205 | 14.733926 | 0.662919 |
| 1 | 11.578248 | 14.662045 | 2.354317 | 1 | 11.577337 | 14.603459 | 2.285806 |
| 1 | 9.252646 | 13.184773 | 3.704126 | 1 | 9.275503 | 13.137753 | 3.656680 |
| 1 | 10.957234 | 12.717036 | 3.881081 | 1 | 10.978778 | 12.659143 | 3.798808 |
| 1 | 9.762593 | 11.520588 | 3.335454 | 1 | 9.768148 | 11.475438 | 3.265724 |
| 8 | 2.172479 | 3.933944 | -3.071771 | 8 | 2.021104 | 3.928807 | -2.791183 |
| 1 | 2.160388 | 2.968418 | -3.168831 | 1 | 2.014031 | 2.960727 | -2.827672 |
| 1 | 1.242755 | 4.203413 | -3.178860 | 1 | 1.108930 | 4.196297 | -2.999728 |
| 8 | -4.348256 | 6.458166 | -5.278016 | 8 | -4.194734 | 6.143744 | -5.260746 |
| 1 | -4.557959 | 7.286528 | -5.736700 | 1 | -4.512609 | 6.900997 | -5.773129 |
| 1 | -3.468252 | 6.604448 | -4.877595 | 1 | -3.351244 | 6.432737 | -4.860194 |
| 9 | 0.602664 | -0.347291 | -1.993030 | 9 | 0.576731 | -0.365470 | -2.001732 |
| 9 | -1.500301 | -1.040639 | -2.592309 | 9 | -1.526397 | -1.001948 | -2.604702 |
| Min $T_1$ (explicit water) | | | | TS $T_1$ (explicit water) | | | |
| 6 | -1.042338 | -4.281280 | 4.000360 | 6 | -0.893194 | -4.236799 | 4.031874 |
| 6 | -1.563355 | -3.101584 | 4.604896 | 6 | -1.395202 | -3.045025 | 4.632358 |
| 6 | -1.733549 | -1.942293 | 3.869671 | 6 | -1.594835 | -1.903317 | 3.880745 |
| 6 | -0.709596 | -4.206270 | 2.614906 | 6 | -0.610840 | -4.191289 | 2.632158 |
| 6 | -0.885311 | -3.039217 | 1.896054 | 6 | -0.816476 | -3.041787 | 1.897300 |
| 6 | -1.403570 | -1.857706 | 2.491436 | 6 | -1.316354 | -1.849336 | 2.488829 |
| 1 | -1.834591 | -3.098068 | 5.653798 | 1 | -1.626881 | -3.020965 | 5.690195 |
| 1 | -2.134732 | -1.062215 | 4.367878 | 1 | -1.979157 | -1.013038 | 4.373536 |
| 1 | -0.614321 | -3.042782 | 0.844300 | 1 | -0.584622 | -3.065761 | 0.836727 |
| 6 | -1.603960 | -0.618635 | 1.792537 | 6 | -1.546955 | -0.628418 | 1.773468 |
| 7 | -0.868485 | -5.435602 | 4.714496 | 7 | -0.690797 | -5.372570 | 4.760700 |
| 1 | -0.310005 | -5.073951 | 2.103619 | 1 | -0.228042 | -5.069018 | 2.125732 |
| 6 | -1.209457 | -5.482503 | 6.133214 | 6 | -0.989348 | -5.395743 | 6.191075 |
| 6 | -0.334028 | -6.631481 | 4.069459 | 6 | -0.173362 | -6.581746 | 4.123123 |
| 1 | 0.676008 | -6.458314 | 3.675966 | 1 | 0.817873 | -6.409292 | 3.685744 |
| 1 | -0.976589 | -6.962651 | 3.243590 | 1 | -0.847256 | -6.936661 | 3.333423 |
| 1 | -0.280503 | -7.436388 | 4.802167 | 1 | -0.083281 | -7.366943 | 4.873009 |
| 1 | -2.274807 | -5.274392 | 6.296804 | 1 | -2.049318 | -5.185001 | 6.380619 |
| 1 | -0.621389 | -4.757525 | 6.710737 | 1 | -0.383926 | -4.661197 | 6.736703 |
| 1 | -0.994258 | -6.479059 | 6.518098 | 1 | -0.762274 | -6.385757 | 6.585166 |
| 6 | -1.325363 | -0.363405 | 0.461399 | 6 | -1.328508 | -0.403517 | 0.427559 |
| 1 | -2.016503 | 0.189762 | 2.394311 | 1 | -1.933399 | 0.193469 | 2.373978 |
| 6 | -1.540615 | 0.877093 | -0.176560 | 6 | -1.575154 | 0.827676 | -0.221080 |
| 1 | -0.910411 | -1.156634 | -0.150746 | 1 | -0.943476 | -1.208979 | -0.187479 |

| | | | | | | | |
|---|---|---|---|---|---|---|---|
| 6 | -2.066756 | 2.088267 | 0.353221 | 6 | -2.072431 | 2.048891 | 0.311561 |
| 6 | -2.100431 | 3.044879 | -0.638722 | 6 | -2.159205 | 2.982602 | -0.701540 |
| 7 | -1.245720 | 1.112040 | -1.522742 | 7 | -1.352837 | 1.033007 | -1.581291 |
| 6 | -1.570266 | 2.411970 | -1.838760 | 6 | -1.699257 | 2.320090 | -1.904833 |
| 1 | -2.388755 | 2.235270 | 1.376340 | 1 | -2.336993 | 2.219854 | 1.347018 |
| 6 | -2.606444 | 4.445326 | -0.437628 | 6 | -2.643646 | 4.390973 | -0.508517 |
| 1 | -2.807390 | 4.609465 | 0.625874 | 1 | -2.812053 | 4.575139 | 0.557102 |
| 1 | -3.544983 | 4.632101 | -0.975463 | 1 | -3.594345 | 4.578494 | -1.024227 |
| 1 | -1.885146 | 5.204822 | -0.757976 | 1 | -1.920546 | 5.132939 | -0.864659 |
| 5 | -0.661636 | 0.080095 | -2.495779 | 5 | -0.806953 | -0.023574 | -2.558264 |
| 7 | -0.478685 | 0.764589 | -3.856376 | 7 | -0.662160 | 0.636861 | -3.941050 |
| 6 | -1.367601 | 2.921341 | -3.151204 | 6 | -1.581225 | 2.799792 | -3.258417 |
| 6 | -0.798733 | 2.073614 | -4.141464 | 6 | -1.009626 | 1.926830 | -4.252305 |
| 6 | -1.739017 | 4.324777 | -3.482934 | 6 | -1.974857 | 4.109478 | -3.596707 |
| 6 | 0.051157 | 0.159485 | -4.999661 | 6 | -0.148399 | 0.014717 | -5.076869 |
| 6 | -0.446975 | 2.326215 | -5.532873 | 6 | -0.697248 | 2.157150 | -5.648227 |
| 6 | 0.065020 | 1.144944 | -6.025619 | 6 | -0.172238 | 0.973053 | -6.127173 |
| 6 | -0.569782 | 3.587777 | -6.340460 | 6 | -0.875614 | 3.395361 | -6.480116 |
| 6 | 0.470334 | -1.187862 | -5.042081 | 6 | 0.288107 | -1.329471 | -5.093714 |
| 1 | 0.427463 | 0.992522 | -7.034330 | 1 | 0.169277 | 0.802991 | -7.139995 |
| 1 | -1.614140 | 3.856760 | -6.544603 | 1 | -1.933764 | 3.637623 | -6.642576 |
| 1 | -0.079343 | 3.446658 | -7.308971 | 1 | -0.424332 | 3.239429 | -7.464959 |
| 1 | -0.094990 | 4.447502 | -5.854121 | 1 | -0.400010 | 4.273664 | -6.030077 |
| 6 | 0.987558 | -1.812935 | -6.162709 | 6 | 0.790872 | -1.967276 | -6.211807 |
| 1 | 0.369418 | -1.754663 | -4.123019 | 1 | 0.213431 | -1.876955 | -4.160969 |
| 6 | 1.427307 | -3.177385 | -6.260764 | 6 | 1.247519 | -3.323598 | -6.294342 |
| 1 | 1.079541 | -1.230733 | -7.078308 | 1 | 0.855958 | -1.400126 | -7.138831 |
| 6 | 1.394991 | -4.108412 | -5.188056 | 6 | 1.248052 | -4.235610 | -5.203468 |
| 6 | 1.932412 | -3.660891 | -7.496204 | 6 | 1.736946 | -3.819112 | -7.532534 |
| 6 | 1.829965 | -5.411831 | -5.334998 | 6 | 1.698969 | -5.532624 | -5.336075 |
| 6 | 2.336245 | -5.889018 | -6.580623 | 6 | 2.189651 | -6.023002 | -6.585071 |
| 6 | 2.371603 | -4.962336 | -7.661593 | 6 | 2.191989 | -5.114510 | -7.684118 |
| 1 | 1.019279 | -3.802861 | -4.215920 | 1 | 0.885542 | -3.917855 | -4.230491 |
| 1 | 1.778608 | -6.074235 | -4.479190 | 1 | 1.674009 | -6.181679 | -4.469266 |
| 7 | 2.767841 | -7.179443 | -6.727647 | 7 | 2.635925 | -7.305519 | -6.717827 |
| 1 | 1.976232 | -2.983133 | -8.346102 | 1 | 1.754569 | -3.154364 | -8.393243 |
| 1 | 2.745562 | -5.266655 | -8.631888 | 1 | 2.553024 | -5.430778 | -8.655202 |
| 6 | 2.719973 | -8.108314 | -5.602189 | 6 | 2.626617 | -8.217095 | -5.575206 |
| 6 | 3.267469 | -7.646943 | -8.017160 | 6 | 3.123989 | -7.788760 | -8.007832 |
| 1 | 4.149875 | -7.077034 | -8.335716 | 1 | 3.993909 | -7.212254 | -8.346451 |
| 1 | 2.500458 | -7.564673 | -8.798191 | 1 | 2.343819 | -7.728765 | -8.776888 |
| 1 | 3.552686 | -8.695108 | -7.929456 | 1 | 3.423575 | -8.831326 | -7.906151 |
| 1 | 1.691367 | -8.261848 | -5.250419 | 1 | 1.608060 | -8.377842 | -5.200134 |
| 1 | 3.324973 | -7.748683 | -4.760104 | 1 | 3.245266 | -7.833761 | -4.754423 |
| 1 | 3.118814 | -9.071512 | -5.919612 | 1 | 3.031886 | -9.179305 | -5.886415 |
| 8 | -0.592930 | 5.225818 | -3.177532 | 8 | -0.438379 | 5.273678 | -3.350534 |
| 1 | -2.584770 | 4.685868 | -2.903283 | 1 | -2.602765 | 4.667817 | -2.921985 |
| 1 | -1.975466 | 4.454818 | -4.534198 | 1 | -2.109595 | 4.370321 | -4.633063 |
| 6 | -0.750138 | 6.536314 | -3.490466 | 6 | -0.649298 | 6.554415 | -3.463147 |
| 6 | 0.371609 | 7.379711 | -3.030871 | 6 | 0.490448 | 7.421765 | -3.011711 |
| 8 | -1.738122 | 6.950534 | -4.085264 | 8 | -1.708528 | 7.062953 | -3.873216 |
| 6 | 0.704339 | 8.634625 | -3.699822 | 6 | 0.846927 | 8.657016 | -3.694184 |
| 6 | 1.093718 | 6.976003 | -1.924828 | 6 | 1.202763 | 7.020292 | -1.905499 |
| 7 | 2.100149 | 7.678196 | -1.366800 | 7 | 2.207214 | 7.726731 | -1.331221 |
| 1 | 0.857508 | 6.047147 | -1.423838 | 1 | 0.964794 | 6.085264 | -1.414657 |
| 6 | 1.848350 | 9.350225 | -3.103700 | 6 | 1.974313 | 9.384836 | -3.081383 |
| 8 | 0.123000 | 9.076287 | -4.708162 | 8 | 0.298471 | 9.076949 | -4.734114 |
| 6 | 2.532131 | 8.874338 | -1.964117 | 6 | 2.639089 | 8.920170 | -1.924239 |
| 6 | 2.798569 | 7.136483 | -0.206939 | 6 | 2.899894 | 7.181777 | -0.171931 |

| | | | | | | | |
|---|---|---|---|---|---|---|---|
| 6 | 2.018344 | 6.481563 | 0.901440 | 6 | 2.114364 | 6.517912 | 0.927683 |
| 6 | 2.674052 | 7.820599 | 1.131774 | 6 | 2.765473 | 7.856805 | 1.171175 |
| 1 | 3.762395 | 6.704255 | -0.465317 | 1 | 3.867693 | 6.753339 | -0.423356 |
| 1 | 2.020347 | 8.686392 | 1.188302 | 1 | 2.109964 | 8.721212 | 1.228706 |
| 1 | 3.566928 | 7.878179 | 1.747670 | 1 | 3.653634 | 7.911759 | 1.794244 |
| 1 | 2.456320 | 5.593437 | 1.346853 | 1 | 2.551083 | 5.628410 | 1.371759 |
| 1 | 0.934376 | 6.466362 | 0.837040 | 1 | 1.031057 | 6.500300 | 0.854233 |
| 6 | 2.288543 | 10.554597 | -3.685894 | 6 | 2.418069 | 10.589433 | -3.662499 |
| 6 | 3.358332 | 11.228451 | -3.158030 | 6 | 3.469842 | 11.276358 | -3.116836 |
| 6 | 4.092513 | 10.762616 | -2.035285 | 6 | 4.184171 | 10.823501 | -1.976125 |
| 6 | 3.643341 | 9.569313 | -1.454273 | 6 | 3.736195 | 9.629810 | -1.398265 |
| 1 | 1.759688 | 10.954351 | -4.544255 | 1 | 1.903655 | 10.978420 | -4.534583 |
| 1 | 4.173340 | 9.164783 | -0.604764 | 1 | 4.253363 | 9.235219 | -0.536107 |
| 9 | 3.709151 | 12.422493 | -3.718617 | 9 | 3.822679 | 12.471624 | -3.677005 |
| 7 | 5.161469 | 11.506317 | -1.525028 | 7 | 5.235252 | 11.584025 | -1.447984 |
| 6 | 6.278348 | 11.806558 | -2.444922 | 6 | 6.372537 | 11.874650 | -2.345032 |
| 6 | 5.653519 | 11.167624 | -0.186292 | 6 | 5.698605 | 11.260710 | -0.095701 |
| 6 | 7.127660 | 12.954561 | -1.906794 | 6 | 7.205855 | 13.032176 | -1.802240 |
| 1 | 5.888043 | 12.082591 | -3.423565 | 1 | 6.004161 | 12.137352 | -3.335948 |
| 1 | 6.905477 | 10.907202 | -2.564673 | 1 | 7.005203 | 10.975843 | -2.439295 |
| 6 | 6.498071 | 12.312587 | 0.377407 | 6 | 6.525362 | 12.415036 | 0.475032 |
| 1 | 6.254548 | 10.242247 | -0.202903 | 1 | 6.304395 | 10.338081 | -0.088890 |
| 1 | 4.800661 | 11.013045 | 0.479945 | 1 | 4.831649 | 11.108071 | 0.552693 |
| 7 | 7.577558 | 12.661844 | -0.546891 | 7 | 7.624578 | 12.758956 | -0.428178 |
| 1 | 6.921909 | 12.010757 | 1.333370 | 1 | 6.928731 | 12.125529 | 1.443609 |
| 1 | 5.857263 | 13.191799 | 0.542002 | 1 | 5.877474 | 13.293304 | 0.615387 |
| 1 | 6.538040 | 13.883866 | -1.909501 | 1 | 6.613578 | 13.959380 | -1.830940 |
| 1 | 8.001871 | 13.097625 | -2.541911 | 1 | 8.094402 | 13.170180 | -2.418330 |
| 6 | 8.838444 | 13.027874 | -0.159643 | 6 | 8.872625 | 13.140211 | -0.015262 |
| 8 | 9.689227 | 13.461306 | -0.938156 | 8 | 9.739160 | 13.569859 | -0.778444 |
| 8 | 9.027120 | 12.835989 | 1.164063 | 8 | 9.030370 | 12.967827 | 1.315246 |
| 6 | 10.327710 | 13.092518 | 1.826092 | 6 | 10.312147 | 13.244406 | 2.005128 |
| 6 | 10.033919 | 12.714504 | 3.280708 | 6 | 9.984879 | 12.886621 | 3.457719 |
| 6 | 10.690399 | 14.576886 | 1.718997 | 6 | 10.667138 | 14.729573 | 1.883960 |
| 6 | 11.407499 | 12.177687 | 1.240108 | 6 | 11.412756 | 12.328858 | 1.460425 |
| 1 | 11.086705 | 11.130521 | 1.282543 | 1 | 11.099093 | 11.279961 | 1.512262 |
| 1 | 12.323725 | 12.275540 | 1.833555 | 1 | 12.313552 | 12.443763 | 2.074129 |
| 1 | 11.635319 | 12.437357 | 0.204464 | 1 | 11.663670 | 12.573574 | 0.426450 |
| 1 | 9.870339 | 15.199288 | 2.094981 | 1 | 9.833164 | 15.351778 | 2.228364 |
| 1 | 10.909257 | 14.863214 | 0.688595 | 1 | 10.911068 | 15.001166 | 0.855206 |
| 1 | 11.576236 | 14.776389 | 2.332788 | 1 | 11.535093 | 14.945235 | 2.517497 |
| 1 | 9.230488 | 13.336759 | 3.689628 | 1 | 9.167461 | 13.509460 | 3.836953 |
| 1 | 10.931022 | 12.863940 | 3.890768 | 1 | 10.865572 | 13.051420 | 4.087419 |
| 1 | 9.735121 | 11.663395 | 3.357035 | 1 | 9.691068 | 11.834775 | 3.542799 |
| 8 | 2.129652 | 3.906368 | -2.903001 | 8 | 2.086907 | 3.979038 | -3.561143 |
| 1 | 2.085302 | 2.937097 | -2.901496 | 1 | 1.920688 | 3.177416 | -4.081591 |
| 1 | 1.208307 | 4.195475 | -3.038318 | 1 | 1.212284 | 4.422675 | -3.495092 |
| 8 | -4.316826 | 6.383846 | -5.171859 | 8 | -3.909782 | 6.412161 | -5.441908 |
| 1 | -4.617466 | 7.236636 | -5.522317 | 1 | -4.069229 | 7.219887 | -5.953997 |
| 1 | -3.428360 | 6.559239 | -4.800555 | 1 | -3.121646 | 6.611570 | -4.885177 |
| 9 | 0.587151 | -0.408181 | -2.013145 | 9 | 0.451686 | -0.500797 | -2.109945 |
| 9 | -1.530627 | -1.042914 | -2.613735 | 9 | -1.689181 | -1.132656 | -2.630845 |
| Products $T_1$ (explicit water) | | | | | | | |
| 6 | -0.568322 | -4.350627 | 6.192108 | | | | |
| 6 | -1.647034 | -3.482659 | 6.547560 | | | | |
| 6 | -2.468747 | -2.949158 | 5.580103 | | | | |
| 6 | -0.384779 | -4.634985 | 4.800319 | | | | |
| 6 | -1.216055 | -4.093763 | 3.847722 | | | | |

| | | | | |
|---|---|---|---|---|
| 6 -2.294277 -3.227988 4.194347 | |
| 1 -1.829799 -3.234168 7.585453 | |
| 1 -3.280195 -2.292246 5.883626 | |
| 1 -1.033338 -4.342221 2.807022 | |
| 6 -3.186457 -2.641479 3.250204 | |
| 7 0.250191 -4.884961 7.134017 | |
| 1 0.418368 -5.287061 4.480513 | |
| 6 0.042947 -4.590383 8.553498 | |
| 6 1.352915 -5.769561 6.751024 | |
| 1 2.060311 -5.254835 6.090762 | |
| 1 0.981326 -6.666483 6.242067 | |
| 1 1.885148 -6.079766 7.648958 | |
| 1 -0.946614 -4.925728 8.885104 | |
| 1 0.137553 -3.516394 8.751337 | |
| 1 0.797346 -5.115283 9.137533 | |
| 6 -3.165148 -2.809823 1.876063 | |
| 1 -3.956344 -1.999880 3.674398 | |
| 6 -4.083625 -2.196077 0.996995 | |
| 1 -2.408609 -3.445411 1.430716 | |
| 6 -5.169899 -1.323403 1.287630 | |
| 6 -5.782836 -0.960588 0.103951 | |
| 7 -4.048621 -2.367196 -0.380137 | |
| 6 -5.053843 -1.627666 -0.945099 | |
| 1 -5.470781 -1.000248 2.275479 | |
| 6 -6.983519 -0.063517 0.018999 | |
| 1 -7.335287 0.170847 1.028153 | |
| 1 -7.813463 -0.532996 -0.522156 | |
| 1 -6.757835 0.888014 -0.478111 | |
| 5 -3.043566 -3.261839 -1.135968 | |
| 7 -3.417992 -3.237857 -2.632837 | |
| 6 -5.242484 -1.607809 -2.390781 | |
| 6 -4.421882 -2.499510 -3.201584 | |
| 6 -6.144587 -0.755811 -2.972193 | |
| 6 -2.790083 -3.981306 -3.622860 | |
| 6 -4.461626 -2.783515 -4.613900 | |
| 6 -3.450574 -3.694725 -4.850638 | |
| 6 -5.390872 -2.263367 -5.671971 | |
| 6 -1.697805 -4.839011 -3.369578 | |
| 1 -3.205333 -4.125597 -5.812335 | |
| 1 -6.444019 -2.425434 -5.413638 | |
| 1 -5.196672 -2.782507 -6.615269 | |
| 1 -5.254262 -1.190325 -5.854706 | |
| 6 -1.056160 -5.577528 -4.349351 | |
| 1 -1.357331 -4.909603 -2.343129 | |
| 6 0.044895 -6.463397 -4.163801 | |
| 1 -1.413821 -5.487809 -5.373289 | |
| 6 0.658898 -6.726221 -2.904293 | |
| 6 0.585035 -7.145254 -5.291574 | |
| 6 1.718071 -7.594636 -2.780265 | |
| 6 2.254577 -8.276928 -3.919649 | |
| 6 1.645251 -8.017401 -5.186385 | |
| 1 0.290960 -6.235552 -2.008765 | |
| 1 2.145784 -7.757249 -1.798877 | |
| 7 3.299175 -9.135438 -3.799172 | |
| 1 0.143419 -6.970656 -6.269569 | |
| 1 2.011840 -8.506984 -6.079860 | |
| 6 3.904658 -9.397136 -2.491426 | |
| 6 3.834691 -9.825559 -4.974616 | |
| 1 4.195177 -9.109197 -5.721638 | |

| | | | | |
|---|---|---|---|---|
| 1 | 3.074609 | -10.466676 | -5.436444 | |
| 1 | 4.671531 | -10.450642 | -4.666986 | |
| 1 | 3.172603 | -9.822810 | -1.795463 | |
| 1 | 4.315654 | -8.479132 | -2.055859 | |
| 1 | 4.716731 | -10.112065 | -2.614902 | |
| 8 | -3.507464 | 2.824693 | -3.350719 | |
| 1 | -6.741967 | -0.071933 | -2.389746 | |
| 1 | -6.279730 | -0.709290 | -4.041529 | |
| 6 | -3.476513 | 3.922120 | -2.713049 | |
| 6 | -2.197178 | 4.261927 | -1.970339 | |
| 8 | -4.443288 | 4.730871 | -2.613775 | |
| 6 | -1.577115 | 5.575062 | -2.008141 | |
| 6 | -1.614139 | 3.259105 | -1.236842 | |
| 7 | -0.508550 | 3.400298 | -0.454808 | |
| 1 | -2.042191 | 2.264346 | -1.240772 | |
| 6 | -0.353465 | 5.698896 | -1.193368 | |
| 8 | -1.988554 | 6.535988 | -2.696519 | |
| 6 | 0.166276 | 4.623611 | -0.436508 | |
| 6 | 0.027878 | 2.245667 | 0.249001 | |
| 6 | -0.906244 | 1.295339 | 0.949777 | |
| 6 | -0.007255 | 2.198126 | 1.757535 | |
| 1 | 0.898058 | 1.816196 | -0.243543 | |
| 1 | -0.477001 | 3.033253 | 2.269548 | |
| 1 | 0.855083 | 1.774812 | 2.264947 | |
| 1 | -0.670677 | 0.237549 | 0.882067 | |
| 1 | -1.968219 | 1.521837 | 0.953080 | |
| 6 | 0.337429 | 6.927458 | -1.160348 | |
| 6 | 1.481592 | 7.061101 | -0.419822 | |
| 6 | 2.052518 | 5.994901 | 0.324183 | |
| 6 | 1.362399 | 4.778820 | 0.294037 | |
| 1 | -0.059856 | 7.772514 | -1.712291 | |
| 1 | 1.762011 | 3.932472 | 0.833023 | |
| 9 | 2.081582 | 8.288965 | -0.367853 | |
| 7 | 3.213275 | 6.201124 | 1.084559 | |
| 6 | 4.417666 | 6.647847 | 0.355024 | |
| 6 | 3.551199 | 5.191870 | 2.091875 | |
| 6 | 5.453257 | 7.217731 | 1.320501 | |
| 1 | 4.146880 | 7.416604 | -0.368001 | |
| 1 | 4.853727 | 5.796016 | -0.194235 | |
| 6 | 4.577433 | 5.747834 | 3.081356 | |
| 1 | 3.953120 | 4.275946 | 1.624716 | |
| 1 | 2.648288 | 4.925905 | 2.648142 | |
| 7 | 5.756338 | 6.249868 | 2.373780 | |
| 1 | 4.876171 | 4.961381 | 3.771919 | |
| 1 | 4.121507 | 6.565444 | 3.659716 | |
| 1 | 5.067777 | 8.145054 | 1.770896 | |
| 1 | 6.372940 | 7.450146 | 0.783708 | |
| 6 | 7.040248 | 6.134035 | 2.833440 | |
| 8 | 8.004144 | 6.691091 | 2.305488 | |
| 8 | 7.110554 | 5.325581 | 3.913534 | |
| 6 | 8.394441 | 4.989259 | 4.571851 | |
| 6 | 7.947227 | 4.057736 | 5.702109 | |
| 6 | 9.041918 | 6.254898 | 5.142343 | |
| 6 | 9.311237 | 4.247782 | 3.594173 | |
| 1 | 8.794309 | 3.381275 | 3.166132 | |
| 1 | 10.193576 | 3.883980 | 4.133069 | |
| 1 | 9.643446 | 4.899025 | 2.783393 | |
| 1 | 8.336045 | 6.787181 | 5.790167 | |
| 1 | 9.373177 | 6.928589 | 4.349787 | |

| | | | | |
|---|---|---|---|---|
| 1 | 9.911286 | 5.973306 | 5.747416 | |
| 1 | 7.256701 | 4.571866 | 6.379442 | |
| 1 | 8.818305 | 3.730641 | 6.279642 | |
| 1 | 7.446213 | 3.170494 | 5.299931 | |
| 8 | -1.412942 | 1.315842 | -4.251775 | |
| 1 | -1.722117 | 1.000132 | -5.114441 | |
| 1 | -2.149649 | 1.884755 | -3.903220 | |
| 8 | -6.786465 | 4.266366 | -3.928494 | |
| 1 | -7.253729 | 5.111032 | -3.837753 | |
| 1 | -5.912917 | 4.399605 | -3.466073 | |
| 9 | -1.725163 | -2.787480 | -0.950271 | |
| 9 | -3.113157 | -4.585566 | -0.643245 | |

| Compound 5 | | | | | | | |
|---|---|---|---|---|---|---|---|
| Min $S_0$ (implicit water) | | | | Min $S_1$ (implicit water) | | | |
| 6 | -2.477169 | 0.095276 | 0.447544 | 6 | -2.633277 | -0.018345 | 0.382904 |
| 6 | -2.446630 | 1.215920 | -0.487608 | 6 | -2.567215 | 1.116075 | -0.496310 |
| 6 | -3.179493 | 2.409007 | -0.214966 | 6 | -3.298085 | 2.310400 | -0.211701 |
| 6 | -2.938891 | 3.290466 | -1.250360 | 6 | -3.004034 | 3.230164 | -1.188079 |
| 7 | -1.785791 | 1.360526 | -1.679311 | 7 | -1.845193 | 1.313745 | -1.652664 |
| 6 | -2.077413 | 2.637063 | -2.171456 | 6 | -2.111255 | 2.601895 | -2.101645 |
| 5 | -0.856458 | 0.361010 | -2.424966 | 5 | -0.903807 | 0.342726 | -2.386497 |
| 7 | -0.509733 | 0.927165 | -3.831378 | 7 | -0.486468 | 0.952518 | -3.736108 |
| 9 | -1.496423 | -0.885136 | -2.553337 | 9 | -1.544273 | -0.893564 | -2.589803 |
| 9 | 0.345310 | 0.206336 | -1.681451 | 9 | 0.256087 | 0.124186 | -1.592511 |
| 6 | -1.607573 | 3.069672 | -3.416273 | 6 | -1.590138 | 3.091539 | -3.310369 |
| 6 | -0.852289 | 2.222096 | -4.234577 | 6 | -0.799618 | 2.252867 | -4.112702 |
| 6 | -1.923680 | 4.467632 | -3.883787 | 6 | -1.895702 | 4.481678 | -3.754240 |
| 6 | 0.155060 | 0.332171 | -4.871739 | 6 | 0.220257 | 0.376554 | -4.768571 |
| 6 | -0.379761 | 2.422437 | -5.558991 | 6 | -0.253805 | 2.500117 | -5.403945 |
| 6 | 0.231992 | 1.250932 | -5.960001 | 6 | 0.357968 | 1.341096 | -5.813968 |
| 6 | 0.744767 | -0.998969 | -4.980844 | 6 | 0.806625 | -0.929748 | -4.886877 |
| 8 | -0.878443 | 5.335248 | -3.375710 | 8 | -0.854961 | 5.360189 | -3.255323 |
| 1 | -2.887012 | 4.813587 | -3.505825 | 1 | -2.854334 | 4.832098 | -3.369856 |
| 1 | -1.945276 | 4.536606 | -4.971913 | 1 | -1.923125 | 4.565805 | -4.841549 |
| 6 | -0.972920 | 6.652127 | -3.720234 | 6 | -0.961304 | 6.664119 | -3.589214 |
| 6 | 0.146924 | 7.450062 | -3.178448 | 6 | 0.159315 | 7.469186 | -3.065113 |
| 8 | -1.895953 | 7.068395 | -4.402228 | 8 | -1.892039 | 7.079459 | -4.253424 |
| 6 | 0.217268 | 8.899411 | -3.351703 | 6 | 0.212635 | 8.913770 | -3.224971 |
| 6 | 1.154834 | 6.794946 | -2.493582 | 6 | 1.181738 | 6.820885 | -2.411708 |
| 7 | 2.230328 | 7.391502 | -1.944158 | 7 | 2.260378 | 7.418262 | -1.883987 |
| 1 | 1.115045 | 5.722381 | -2.365568 | 1 | 1.150254 | 5.746958 | -2.292617 |
| 6 | 1.433997 | 9.517976 | -2.785676 | 6 | 1.434024 | 9.537034 | -2.682952 |
| 8 | -0.641787 | 9.595605 | -3.923184 | 8 | -0.659980 | 9.605920 | -3.765334 |
| 6 | 2.419545 | 8.774816 | -2.102581 | 6 | 2.433163 | 8.799178 | -2.034699 |
| 6 | 3.246325 | 6.577750 | -1.286382 | 6 | 3.294835 | 6.612227 | -1.260941 |
| 6 | 2.833522 | 5.439648 | -0.391969 | 6 | 2.906155 | 5.482072 | -0.356669 |
| 6 | 3.443526 | 6.683500 | 0.205603 | 6 | 3.528062 | 6.722517 | 0.218035 |
| 1 | 4.130689 | 6.436262 | -1.903376 | 1 | 4.161030 | 6.470917 | -1.901407 |
| 1 | 2.776821 | 7.347047 | 0.749078 | 1 | 2.876049 | 7.389804 | 0.772842 |
| 1 | 4.463459 | 6.651471 | 0.577878 | 1 | 4.555538 | 6.690456 | 0.565775 |
| 1 | 3.428680 | 4.533322 | -0.449942 | 1 | 3.498841 | 4.576132 | -0.423695 |
| 1 | 1.775072 | 5.273566 | -0.215055 | 1 | 1.852384 | 5.318223 | -0.156711 |
| 6 | 1.630999 | 10.905041 | -2.926504 | 6 | 1.611896 | 10.922418 | -2.817578 |
| 6 | 2.757646 | 11.499941 | -2.421697 | 6 | 2.739910 | 11.518776 | -2.335567 |
| 6 | 3.786988 | 10.779089 | -1.761135 | 6 | 3.783383 | 10.801302 | -1.707618 |
| 6 | 3.580270 | 9.401067 | -1.614752 | 6 | 3.596583 | 9.429025 | -1.568212 |

| | | | | | | | |
|---|---|---|---|---|---|---|---|
| 1 | 0.871623 | 11.501639 | -3.420059 | 1 | 0.836430 | 11.514419 | -3.289716 |
| 1 | 4.337680 | 8.803730 | -1.129326 | 1 | 4.368876 | 8.834905 | -1.104003 |
| 9 | 2.873939 | 12.855952 | -2.527888 | 9 | 2.850924 | 12.868786 | -2.432747 |
| 7 | 4.903504 | 11.443862 | -1.242467 | 7 | 4.904413 | 11.474827 | -1.216844 |
| 6 | 5.721594 | 12.231383 | -2.188320 | 6 | 5.691977 | 12.238508 | -2.195555 |
| 6 | 5.746378 | 10.718444 | -0.287102 | 6 | 5.771079 | 10.750527 | -0.293020 |
| 6 | 6.614212 | 13.216974 | -1.439147 | 6 | 6.612984 | 13.221003 | -1.491008 |
| 1 | 5.073093 | 12.784602 | -2.866457 | 1 | 5.025686 | 12.788880 | -2.857488 |
| 1 | 6.346702 | 11.549031 | -2.788541 | 1 | 6.291180 | 11.545221 | -2.807433 |
| 6 | 6.641474 | 11.692103 | 0.483094 | 6 | 6.692799 | 11.718862 | 0.437515 |
| 1 | 6.374756 | 9.967837 | -0.796686 | 1 | 6.378088 | 9.996941 | -0.821385 |
| 1 | 5.106706 | 10.200796 | 0.432641 | 1 | 5.154858 | 10.237305 | 0.448612 |
| 7 | 7.420419 | 12.517089 | -0.440715 | 7 | 7.441386 | 12.526674 | -0.516320 |
| 1 | 7.316250 | 11.129797 | 1.125813 | 1 | 7.385642 | 11.159719 | 1.061938 |
| 1 | 6.016465 | 12.340365 | 1.115391 | 1 | 6.093878 | 12.376108 | 1.083016 |
| 1 | 5.991534 | 13.977301 | -0.943853 | 1 | 6.013706 | 13.990300 | -0.984119 |
| 1 | 7.281922 | 13.718806 | -2.139352 | 1 | 7.261819 | 13.709687 | -2.216207 |
| 6 | 8.721357 | 12.890673 | -0.238415 | 6 | 8.743763 | 12.890125 | -0.359521 |
| 8 | 9.316240 | 13.696163 | -0.956169 | 8 | 9.319633 | 13.682599 | -1.098882 |
| 8 | 9.261597 | 12.255790 | 0.824526 | 8 | 9.313465 | 12.266812 | 0.683831 |
| 6 | 10.678916 | 12.433825 | 1.218572 | 6 | 10.730295 | 12.447182 | 1.027038 |
| 6 | 10.796663 | 11.514506 | 2.437634 | 6 | 10.890868 | 11.546472 | 2.246288 |
| 6 | 10.937354 | 13.890293 | 1.616743 | 6 | 11.000948 | 13.901421 | 1.398430 |
| 6 | 11.602259 | 11.957082 | 0.093152 | 6 | 11.613108 | 11.960679 | -0.117373 |
| 1 | 11.349067 | 10.932276 | -0.201695 | 1 | 11.348408 | 10.934799 | -0.393089 |
| 1 | 12.638338 | 11.960388 | 0.450583 | 1 | 12.658583 | 11.965488 | 0.206601 |
| 1 | 11.535289 | 12.605207 | -0.782907 | 1 | 11.518110 | 12.600189 | -0.995610 |
| 1 | 10.220650 | 14.210736 | 2.381533 | 1 | 10.308472 | 14.229764 | 2.180209 |
| 1 | 10.863053 | 14.560407 | 0.758228 | 1 | 10.899882 | 14.560134 | 0.535371 |
| 1 | 11.944858 | 13.974673 | 2.039968 | 1 | 12.019658 | 13.989237 | 1.789073 |
| 1 | 10.103845 | 11.825272 | 3.227182 | 1 | 10.224686 | 11.865965 | 3.053295 |
| 1 | 11.815759 | 11.556243 | 2.836439 | 1 | 11.921466 | 11.593368 | 2.609615 |
| 1 | 10.573955 | 10.476961 | 2.165898 | 1 | 10.659755 | 10.507382 | 1.993274 |
| 1 | -3.340495 | 4.289602 | -1.355760 | 1 | -3.384571 | 4.239094 | -1.266669 |
| 1 | -0.495652 | 3.323881 | -6.145945 | 1 | -0.325870 | 3.427900 | -5.954437 |
| 6 | -4.019347 | 2.537027 | 1.019654 | 6 | -4.160740 | 2.414077 | 1.000780 |
| 6 | -4.608257 | 1.159849 | 1.363103 | 6 | -4.754814 | 1.039291 | 1.310043 |
| 1 | -4.818078 | 3.272307 | 0.875775 | 1 | -4.952431 | 3.154530 | 0.855435 |
| 1 | -3.405462 | 2.892299 | 1.861447 | 1 | -3.559062 | 2.754213 | 1.855717 |
| 6 | -3.540165 | 0.083756 | 1.393874 | 6 | -3.702470 | -0.041988 | 1.326220 |
| 1 | -5.358043 | 0.896384 | 0.601357 | 1 | -5.494492 | 0.793621 | 0.534803 |
| 1 | -5.132356 | 1.193128 | 2.323708 | 1 | -5.289233 | 1.054267 | 2.263675 |
| 6 | -1.516110 | -0.938443 | 0.514013 | 6 | -1.705216 | -1.087374 | 0.411375 |
| 6 | -1.606631 | -1.951071 | 1.455732 | 6 | -1.826666 | -2.120354 | 1.312771 |
| 6 | -2.669902 | -1.966268 | 2.374077 | 6 | -2.886991 | -2.134743 | 2.230766 |
| 6 | -3.627318 | -0.943225 | 2.338090 | 6 | -3.815609 | -1.089528 | 2.229909 |
| 8 | -2.679634 | -2.998656 | 3.260418 | 8 | -2.926582 | -3.186217 | 3.076205 |
| 6 | -3.731904 | -3.071227 | 4.228784 | 6 | -3.973257 | -3.260743 | 4.040062 |
| 1 | -3.530534 | -3.969682 | 4.812802 | 1 | -3.797065 | -4.180155 | 4.596451 |
| 1 | -4.708826 | -3.160054 | 3.740475 | 1 | -4.951157 | -3.308074 | 3.551142 |
| 1 | -3.721450 | -2.194383 | 4.885902 | 1 | -3.937371 | -2.405362 | 4.721715 |
| 1 | -4.450975 | -0.931664 | 3.043428 | 1 | -4.642012 | -1.084674 | 2.930819 |
| 1 | -0.675846 | -0.937594 | -0.165578 | 1 | -0.865918 | -1.086451 | -0.267461 |
| 1 | -0.856544 | -2.735215 | 1.501948 | 1 | -1.103256 | -2.929082 | 1.334271 |
| 6 | 0.890627 | 0.872173 | -7.252072 | 6 | 1.103892 | 1.021255 | -7.065866 |
| 6 | 1.028795 | -1.464638 | -6.302360 | 6 | 1.210646 | -1.340263 | -6.198052 |
| 6 | 1.098609 | -1.824308 | -3.897878 | 6 | 1.053201 | -1.814226 | -3.816996 |
| 6 | 1.692481 | -3.071561 | -4.084342 | 6 | 1.661476 | -3.043514 | -4.007436 |
| 6 | 1.951587 | -3.524479 | -5.385319 | 6 | 2.046750 | -3.429983 | -5.295378 |

| | | | | | | | |
|---|---|---|---|---|---|---|---|
| 6 | 1.619038 | -2.709635 | -6.480632 | 6 | 1.816326 | -2.566687 | -6.375994 |
| 8 | 2.525721 | -4.720681 | -5.687950 | 8 | 2.646583 | -4.601936 | -5.593915 |
| 6 | 2.895578 | -5.598200 | -4.618472 | 6 | 2.911249 | -5.527683 | -4.543543 |
| 1 | 3.324873 | -6.478679 | -5.097361 | 1 | 3.385853 | -6.385198 | -5.018273 |
| 1 | 2.018870 | -5.889324 | -4.029142 | 1 | 1.982381 | -5.842617 | -4.058302 |
| 1 | 3.643472 | -5.130540 | -3.968348 | 1 | 3.590729 | -5.093814 | -3.803544 |
| 1 | 0.931190 | -1.485384 | -2.885396 | 1 | 0.789169 | -1.524119 | -2.811208 |
| 1 | 1.954214 | -3.666811 | -3.217301 | 1 | 1.840084 | -3.683344 | -3.152118 |
| 1 | 1.834461 | -3.074098 | -7.481699 | 1 | 2.121362 | -2.889069 | -7.367232 |
| 6 | 0.655151 | -0.624812 | -7.507981 | 6 | 0.922330 | -0.462160 | -7.390740 |
| 1 | -0.410839 | -0.779939 | -7.734231 | 1 | -0.118356 | -0.628379 | -7.702923 |
| 1 | 1.217122 | -0.960639 | -8.385395 | 1 | 1.556291 | -0.755692 | -8.231627 |
| 1 | 1.972414 | 1.068499 | -7.199213 | 1 | 2.172536 | 1.238150 | -6.926143 |
| 1 | 0.498841 | 1.471235 | -8.080930 | 1 | 0.759457 | 1.645215 | -7.895487 |
| Min $T_1$ (implicit water) | | | | TS $T_1$ (implicit water) | | | |
| 6 | -2.824750 | -0.067727 | 0.372666 | 6 | -2.943735 | -0.237029 | 0.362506 |
| 6 | -2.721684 | 1.072777 | -0.502227 | 6 | -3.078928 | 0.885215 | -0.527012 |
| 6 | -3.468819 | 2.270066 | -0.249691 | 6 | -4.094884 | 1.882063 | -0.314380 |
| 6 | -3.133634 | 3.192024 | -1.214485 | 6 | -3.947732 | 2.840853 | -1.288727 |
| 7 | -1.940768 | 1.280072 | -1.641374 | 7 | -2.342372 | 1.249938 | -1.643245 |
| 6 | -2.193679 | 2.564162 | -2.094874 | 6 | -2.866498 | 2.433802 | -2.118829 |
| 5 | -0.962474 | 0.310044 | -2.343282 | 5 | -1.148754 | 0.506722 | -2.306849 |
| 7 | -0.480264 | 0.937772 | -3.671204 | 7 | -0.802890 | 1.193788 | -3.657937 |
| 9 | -1.597135 | -0.927687 | -2.590932 | 9 | -1.489885 | -0.836219 | -2.530616 |
| 9 | 0.164074 | 0.084126 | -1.495039 | 9 | -0.023694 | 0.583064 | -1.449051 |
| 6 | -1.619311 | 3.071291 | -3.281059 | 6 | -2.380773 | 3.057931 | -3.319605 |
| 6 | -0.784440 | 2.233932 | -4.052229 | 6 | -1.369850 | 2.376964 | -4.081854 |
| 6 | -1.913004 | 4.463287 | -3.730487 | 6 | -2.873630 | 4.285370 | -3.735447 |
| 6 | 0.291625 | 0.368898 | -4.686704 | 6 | 0.070154 | 0.770818 | -4.648201 |
| 6 | -0.175105 | 2.501341 | -5.321281 | 6 | -0.844600 | 2.725235 | -5.357443 |
| 6 | 0.469563 | 1.351627 | -5.715703 | 6 | 0.031732 | 1.730090 | -5.720924 |
| 6 | 0.894496 | -0.935851 | -4.791387 | 6 | 0.937438 | -0.374762 | -4.709957 |
| 8 | -0.886911 | 5.361672 | -3.186170 | 8 | -1.569210 | 5.871954 | -2.784426 |
| 1 | -2.884856 | 4.814769 | -3.379902 | 1 | -3.704808 | 4.750565 | -3.225018 |
| 1 | -1.897066 | 4.559130 | -4.818100 | 1 | -2.596670 | 4.718502 | -4.685626 |
| 6 | -0.987528 | 6.672633 | -3.523478 | 6 | -1.034119 | 6.674368 | -3.629822 |
| 6 | 0.140249 | 7.473706 | -2.992518 | 6 | 0.124723 | 7.494976 | -3.088504 |
| 8 | -1.914130 | 7.099773 | -4.197405 | 8 | -1.358135 | 6.800119 | -4.833214 |
| 6 | 0.126828 | 8.933988 | -3.003784 | 6 | 0.293905 | 8.909338 | -3.374102 |
| 6 | 1.239469 | 6.805741 | -2.486138 | 6 | 1.043521 | 6.849582 | -2.298908 |
| 7 | 2.341421 | 7.398206 | -1.983119 | 7 | 2.165955 | 7.417471 | -1.777957 |
| 1 | 1.259435 | 5.724845 | -2.477931 | 1 | 0.904453 | 5.805042 | -2.049983 |
| 6 | 1.367860 | 9.554930 | -2.496780 | 6 | 1.544108 | 9.493877 | -2.852323 |
| 8 | -0.822680 | 9.638815 | -3.394160 | 8 | -0.535799 | 9.613942 | -3.991350 |
| 6 | 2.451946 | 8.798181 | -2.003878 | 6 | 2.465089 | 8.748696 | -2.080571 |
| 6 | 3.459016 | 6.576295 | -1.534361 | 6 | 3.101790 | 6.598682 | -1.022872 |
| 6 | 3.199599 | 5.323597 | -0.741221 | 6 | 2.583023 | 5.613173 | -0.009367 |
| 6 | 3.814255 | 6.526325 | -0.068873 | 6 | 3.306268 | 6.854685 | 0.450618 |
| 1 | 4.276351 | 6.555524 | -2.251635 | 1 | 3.972838 | 6.303239 | -1.604540 |
| 1 | 3.181921 | 7.085553 | 0.614901 | 1 | 2.704888 | 7.636394 | 0.906165 |
| 1 | 4.869413 | 6.511987 | 0.188730 | 1 | 4.317404 | 6.768748 | 0.838423 |
| 1 | 3.825741 | 4.465545 | -0.966488 | 1 | 3.090234 | 4.654577 | 0.046516 |
| 1 | 2.175760 | 5.077497 | -0.475697 | 1 | 1.512625 | 5.568739 | 0.168291 |
| 6 | 1.487637 | 10.957980 | -2.500129 | 6 | 1.847762 | 10.843081 | -3.126122 |
| 6 | 2.634289 | 11.556905 | -2.048288 | 6 | 3.012866 | 11.399017 | -2.669019 |
| 6 | 3.758442 | 10.827462 | -1.579370 | 6 | 3.980364 | 10.671504 | -1.926606 |
| 6 | 3.629415 | 9.432527 | -1.568112 | 6 | 3.670380 | 9.336881 | -1.644930 |
| 1 | 0.654636 | 11.560858 | -2.844821 | 1 | 1.138487 | 11.442001 | -3.687246 |

| | | | |
|---|---|---|---|
| 1 | 4.459546 | 8.829429 | -1.231365 |
| 9 | 2.679471 | 12.921083 | -2.014077 |
| 7 | 4.893468 | 11.494746 | -1.105052 |
| 6 | 5.571839 | 12.418959 | -2.037446 |
| 6 | 5.863608 | 10.721269 | -0.324296 |
| 6 | 6.493797 | 13.369862 | -1.279223 |
| 1 | 4.831684 | 13.002405 | -2.583521 |
| 1 | 6.159847 | 11.837336 | -2.767292 |
| 6 | 6.791625 | 11.657497 | 0.453427 |
| 1 | 6.467051 | 10.063432 | -0.973174 |
| 1 | 5.326995 | 10.097210 | 0.395272 |
| 7 | 7.430962 | 12.616074 | -0.448437 |
| 1 | 7.555109 | 11.069729 | 0.959536 |
| 1 | 6.209288 | 12.200844 | 1.212581 |
| 1 | 5.893546 | 14.039389 | -0.644774 |
| 1 | 7.061333 | 13.977277 | -1.984106 |
| 6 | 8.728838 | 13.036256 | -0.339904 |
| 8 | 9.208482 | 13.943708 | -1.021384 |
| 8 | 9.406058 | 12.323495 | 0.586526 |
| 6 | 10.851167 | 12.525047 | 0.843971 |
| 6 | 11.135520 | 11.488222 | 1.934556 |
| 6 | 11.102391 | 13.941708 | 1.369663 |
| 6 | 11.659829 | 12.213835 | -0.419086 |
| 1 | 11.412816 | 11.214747 | -0.795968 |
| 1 | 12.728336 | 12.229590 | -0.175739 |
| 1 | 11.472360 | 12.946693 | -1.206380 |
| 1 | 10.462543 | 14.148137 | 2.235247 |
| 1 | 10.913649 | 14.693959 | 0.601521 |
| 1 | 12.146552 | 14.026119 | 1.692048 |
| 1 | 10.526043 | 11.682186 | 2.823876 |
| 1 | 12.191100 | 11.532936 | 2.222736 |
| 1 | 10.917705 | 10.476378 | 1.575822 |
| 1 | -3.510885 | 4.201108 | -1.308392 |
| 1 | -0.228609 | 3.436029 | -5.862550 |
| 6 | -4.390255 | 2.371044 | 0.924345 |
| 6 | -4.997676 | 0.988496 | 1.207805 |
| 1 | -5.176702 | 3.111163 | 0.743462 |
| 1 | -3.828892 | 2.711905 | 1.807748 |
| 6 | -3.940890 | -0.094317 | 1.270983 |
| 1 | -5.704773 | 0.743813 | 0.400378 |
| 1 | -5.574248 | 1.004299 | 2.138074 |
| 6 | -1.893241 | -1.137582 | 0.444998 |
| 6 | -2.052076 | -2.174652 | 1.344936 |
| 6 | -3.155580 | -2.192254 | 2.219252 |
| 6 | -4.090166 | -1.145510 | 2.173917 |
| 8 | -3.226260 | -3.249216 | 3.067604 |
| 6 | -4.317347 | -3.329374 | 3.993552 |
| 1 | -4.155189 | -4.248982 | 4.556059 |
| 1 | -5.275266 | -3.383519 | 3.464878 |
| 1 | -4.313712 | -2.472744 | 4.676386 |
| 1 | -4.947716 | -1.142782 | 2.837662 |
| 1 | -1.023639 | -1.135360 | -0.195360 |
| 1 | -1.325451 | -2.980129 | 1.397967 |
| 6 | 1.276711 | 1.052652 | -6.939444 |
| 6 | 1.365935 | -1.328214 | -6.092520 |
| 6 | 1.100131 | -1.835338 | -3.718535 |
| 6 | 1.732516 | -3.060924 | -3.895236 |
| 6 | 2.186273 | -3.429461 | -5.171909 |
| 6 | 1.996111 | -2.551006 | -6.255879 |

| | | | |
|---|---|---|---|
| 1 | 4.377422 | 8.737118 | -1.090808 |
| 9 | 3.236395 | 12.726559 | -2.909742 |
| 7 | 5.143880 | 11.304587 | -1.465255 |
| 6 | 6.028648 | 11.902159 | -2.485990 |
| 6 | 5.923009 | 10.622319 | -0.428572 |
| 6 | 6.998531 | 12.891769 | -1.846710 |
| 1 | 5.431190 | 12.424140 | -3.232666 |
| 1 | 6.596217 | 11.104449 | -2.994830 |
| 6 | 6.894259 | 11.598377 | 0.238921 |
| 1 | 6.488840 | 9.771147 | -0.845794 |
| 1 | 5.242436 | 10.240250 | 0.337087 |
| 7 | 7.740527 | 12.250923 | -0.761591 |
| 1 | 7.520486 | 11.057210 | 0.945825 |
| 1 | 6.324069 | 12.360743 | 0.790787 |
| 1 | 6.440200 | 13.755081 | -1.453744 |
| 1 | 7.708642 | 13.249695 | -2.592191 |
| 6 | 9.056624 | 12.572459 | -0.565814 |
| 8 | 9.712350 | 13.259513 | -1.350583 |
| 8 | 9.537503 | 12.028815 | 0.573528 |
| 6 | 10.951924 | 12.178786 | 0.988454 |
| 6 | 10.993418 | 11.387833 | 2.299111 |
| 6 | 11.273201 | 13.655186 | 1.240461 |
| 6 | 11.878202 | 11.543293 | -0.052923 |
| 1 | 11.580733 | 10.506657 | -0.248335 |
| 1 | 12.903318 | 11.534177 | 0.334733 |
| 1 | 11.866927 | 12.098957 | -0.992628 |
| 1 | 10.555547 | 14.087862 | 1.946884 |
| 1 | 11.252570 | 14.233717 | 0.314932 |
| 1 | 12.273472 | 13.736330 | 1.681055 |
| 1 | 10.299161 | 11.812868 | 3.032120 |
| 1 | 12.003812 | 11.421263 | 2.720218 |
| 1 | 10.724110 | 10.339617 | 2.129772 |
| 1 | -4.548193 | 3.730201 | -1.419805 |
| 1 | -1.106769 | 3.603582 | -5.930604 |
| 6 | -5.055571 | 1.775910 | 0.824629 |
| 6 | -5.333257 | 0.292297 | 1.112582 |
| 1 | -5.984685 | 2.310494 | 0.603446 |
| 1 | -4.620520 | 2.247077 | 1.718762 |
| 6 | -4.057058 | -0.514009 | 1.223102 |
| 1 | -5.938895 | -0.119636 | 0.291475 |
| 1 | -5.924472 | 0.182642 | 2.026575 |
| 6 | -1.786939 | -1.053983 | 0.483641 |
| 6 | -1.730683 | -2.089590 | 1.392227 |
| 6 | -2.836307 | -2.361656 | 2.225752 |
| 6 | -3.989798 | -1.564965 | 2.133358 |
| 8 | -2.688568 | -3.395481 | 3.080613 |
| 6 | -3.762437 | -3.735096 | 3.971766 |
| 1 | -3.401830 | -4.587582 | 4.546836 |
| 1 | -4.657912 | -4.018410 | 3.409190 |
| 1 | -3.986000 | -2.900799 | 4.644443 |
| 1 | -4.846708 | -1.760812 | 2.767790 |
| 1 | -0.917132 | -0.848617 | -0.122415 |
| 1 | -0.837594 | -2.699311 | 1.488172 |
| 6 | 0.873371 | 1.558219 | -6.943685 |
| 6 | 1.470331 | -0.711558 | -6.003611 |
| 6 | 1.342347 | -1.155387 | -3.599752 |
| 6 | 2.218331 | -2.221441 | -3.734396 |
| 6 | 2.723302 | -2.546397 | -5.007327 |
| 6 | 2.341516 | -1.779688 | -6.126139 |

| | | | | | | | |
|---|---|---|---|---|---|---|---|
| 8 | 2.817612 | -4.595816 | -5.460393 | 8 | 3.577881 | -3.561268 | -5.255430 |
| 6 | 3.048268 | -5.539746 | -4.406731 | 6 | 4.015577 | -4.394945 | -4.170763 |
| 1 | 3.556532 | -6.382285 | -4.875992 | 1 | 4.681834 | -5.129800 | -4.621965 |
| 1 | 2.101774 | -5.875674 | -3.969495 | 1 | 3.164772 | -4.901743 | -3.704099 |
| 1 | 3.688270 | -5.108300 | -3.629364 | 1 | 4.561388 | -3.806919 | -3.425879 |
| 1 | 0.785001 | -1.561756 | -2.722394 | 1 | 0.990725 | -0.904398 | -2.609926 |
| 1 | 1.876048 | -3.709361 | -3.038726 | 1 | 2.511371 | -2.781190 | -2.854262 |
| 1 | 2.350870 | -2.857132 | -7.236533 | 1 | 2.744987 | -2.050057 | -7.097906 |
| 6 | 1.125078 | -0.435277 | -7.291458 | 6 | 1.040299 | 0.058225 | -7.233674 |
| 1 | 0.101278 | -0.605722 | -7.657902 | 1 | 0.076581 | -0.343063 | -7.581099 |
| 1 | 1.802486 | -0.710741 | -8.105826 | 1 | 1.759330 | -0.099945 | -8.042954 |
| 1 | 2.337160 | 1.277338 | -6.747968 | 1 | 1.862031 | 2.011687 | -6.777342 |
| 1 | 0.965257 | 1.684281 | -7.777928 | 1 | 0.427189 | 2.073666 | -7.799960 |
| Products $T_1$ (implicit water) | | | | | | | |
| 6 | -2.717295 | -1.299929 | 0.164760 | | | | |
| 6 | -3.022477 | 0.055496 | -0.188282 | | | | |
| 6 | -4.222493 | 0.697616 | 0.293979 | | | | |
| 6 | -4.210570 | 1.992461 | -0.163399 | | | | |
| 7 | -2.318029 | 0.970638 | -0.948907 | | | | |
| 6 | -3.032466 | 2.149605 | -0.942738 | | | | |
| 5 | -0.987665 | 0.765853 | -1.735558 | | | | |
| 7 | -0.731992 | 2.007345 | -2.638878 | | | | |
| 9 | -1.083199 | -0.385877 | -2.526623 | | | | |
| 9 | 0.077059 | 0.641175 | -0.814131 | | | | |
| 6 | -2.610641 | 3.304711 | -1.699632 | | | | |
| 6 | -1.476007 | 3.166926 | -2.581218 | | | | |
| 6 | -3.290641 | 4.498152 | -1.614692 | | | | |
| 6 | 0.221538 | 2.181226 | -3.625316 | | | | |
| 6 | -0.991357 | 4.106487 | -3.533459 | | | | |
| 6 | 0.046635 | 3.496943 | -4.195513 | | | | |
| 6 | 1.268683 | 1.312666 | -4.078688 | | | | |
| 8 | -3.113745 | 8.950511 | -2.601058 | | | | |
| 1 | -4.144569 | 4.606382 | -0.955878 | | | | |
| 1 | -3.000060 | 5.366903 | -2.200135 | | | | |
| 6 | -2.233475 | 8.162894 | -3.053464 | | | | |
| 6 | -0.777163 | 8.582614 | -2.872908 | | | | |
| 8 | -2.444395 | 7.040830 | -3.599597 | | | | |
| 6 | 0.198613 | 8.543824 | -3.946734 | | | | |
| 6 | -0.390970 | 9.013268 | -1.629827 | | | | |
| 7 | 0.878707 | 9.372711 | -1.280030 | | | | |
| 1 | -1.118540 | 9.088743 | -0.830913 | | | | |
| 6 | 1.577673 | 8.871258 | -3.539520 | | | | |
| 8 | -0.072875 | 8.276970 | -5.140416 | | | | |
| 6 | 1.904320 | 9.262512 | -2.220089 | | | | |
| 6 | 1.168422 | 9.735865 | 0.097730 | | | | |
| 6 | 0.229018 | 10.647659 | 0.842361 | | | | |
| 6 | 1.592343 | 11.145794 | 0.431133 | | | | |
| 1 | 1.631239 | 8.929327 | 0.663352 | | | | |
| 1 | 1.632010 | 11.858673 | -0.387661 | | | | |
| 1 | 2.362779 | 11.271706 | 1.186691 | | | | |
| 1 | 0.044350 | 10.415062 | 1.886956 | | | | |
| 1 | -0.627742 | 11.051637 | 0.311206 | | | | |
| 6 | 2.620916 | 8.792405 | -4.485162 | | | | |
| 6 | 3.911541 | 9.067667 | -4.119945 | | | | |
| 6 | 4.283668 | 9.423031 | -2.796407 | | | | |
| 6 | 3.246069 | 9.515530 | -1.863800 | | | | |
| 1 | 2.386539 | 8.528343 | -5.510828 | | | | |
| 1 | 3.478180 | 9.775000 | -0.841213 | | | | |

| | | | |
|---|---|---|---|
| 9 | 4.881373 | 9.032157 | -5.084294 |
| 7 | 5.621911 | 9.716385 | -2.491210 |
| 6 | 6.608430 | 8.643393 | -2.729742 |
| 6 | 5.893066 | 10.408421 | -1.228501 |
| 6 | 8.025482 | 9.209094 | -2.755624 |
| 1 | 6.403393 | 8.159133 | -3.684004 |
| 1 | 6.528032 | 7.883798 | -1.933332 |
| 6 | 7.304482 | 10.999453 | -1.236202 |
| 1 | 5.786558 | 9.728239 | -0.365394 |
| 1 | 5.179186 | 11.228073 | -1.109611 |
| 7 | 8.296187 | 9.964155 | -1.532858 |
| 1 | 7.517905 | 11.437618 | -0.262972 |
| 1 | 7.364044 | 11.792783 | -1.996457 |
| 1 | 8.145190 | 9.866235 | -3.630427 |
| 1 | 8.748092 | 8.396619 | -2.831900 |
| 6 | 9.542041 | 9.905811 | -0.969053 |
| 8 | 10.420736 | 9.126532 | -1.341500 |
| 8 | 9.670878 | 10.788266 | 0.045628 |
| 6 | 10.902310 | 10.881764 | 0.864056 |
| 6 | 10.547660 | 11.987678 | 1.862146 |
| 6 | 12.086094 | 11.306110 | -0.010673 |
| 6 | 11.152720 | 9.558371 | 1.593691 |
| 1 | 10.264566 | 9.260779 | 2.162814 |
| 1 | 11.980366 | 9.687486 | 2.300508 |
| 1 | 11.412290 | 8.758586 | 0.897300 |
| 1 | 11.847116 | 12.224830 | -0.558496 |
| 1 | 12.355881 | 10.526866 | -0.725952 |
| 1 | 12.952648 | 11.508641 | 0.629134 |
| 1 | 10.324009 | 12.924816 | 1.340906 |
| 1 | 11.391449 | 12.161332 | 2.538303 |
| 1 | 9.676026 | 11.705290 | 2.462530 |
| 1 | -4.961526 | 2.749826 | 0.012598 |
| 1 | -1.384843 | 5.106242 | -3.690626 |
| 6 | -5.198408 | -0.033189 | 1.155267 |
| 6 | -5.196182 | -1.521580 | 0.773499 |
| 1 | -6.201330 | 0.393260 | 1.056702 |
| 1 | -4.907384 | 0.074285 | 2.210746 |
| 6 | -3.795579 | -2.087670 | 0.695193 |
| 1 | -5.674826 | -1.635979 | -0.210359 |
| 1 | -5.793183 | -2.100260 | 1.484201 |
| 6 | -1.429416 | -1.899313 | 0.074117 |
| 6 | -1.214974 | -3.198535 | 0.473978 |
| 6 | -2.285366 | -3.969484 | 0.981991 |
| 6 | -3.566998 | -3.400829 | 1.089500 |
| 8 | -1.978879 | -5.227724 | 1.343256 |
| 6 | -3.000420 | -6.083241 | 1.884573 |
| 1 | -2.502345 | -7.029888 | 2.091071 |
| 1 | -3.802300 | -6.234888 | 1.155424 |
| 1 | -3.402498 | -5.663043 | 2.811612 |
| 1 | -4.395786 | -3.981955 | 1.476879 |
| 1 | -0.590285 | -1.320645 | -0.282040 |
| 1 | -0.227231 | -3.645034 | 0.418636 |
| 6 | 0.937284 | 3.987681 | -5.289686 |
| 6 | 1.885046 | 1.645178 | -5.338028 |
| 6 | 1.770290 | 0.195499 | -3.363094 |
| 6 | 2.816265 | -0.571260 | -3.845596 |
| 6 | 3.402102 | -0.241931 | -5.084661 |
| 6 | 2.925463 | 0.867966 | -5.812078 |
| 8 | 4.420348 | -0.916763 | -5.648810 |

| | | | | | | | |
|---|---|---|---|---|---|---|---|
| 6 | 4.973930 | -2.061176 | -4.976495 | | | | |
| 1 | 5.766108 | -2.423240 | -5.630988 | | | | |
| 1 | 4.213882 | -2.837778 | -4.846824 | | | | |
| 1 | 5.393883 | -1.773548 | -4.007627 | | | | |
| 1 | 1.355911 | -0.054415 | -2.397738 | | | | |
| 1 | 3.178511 | -1.406121 | -3.258057 | | | | |
| 1 | 3.395169 | 1.098860 | -6.763821 | | | | |
| 6 | 1.365040 | 2.799702 | -6.165699 | | | | |
| 1 | 0.494711 | 2.454368 | -6.742990 | | | | |
| 1 | 2.123233 | 3.109476 | -6.890848 | | | | |
| 1 | 1.829710 | 4.460123 | -4.852812 | | | | |
| 1 | 0.433310 | 4.752808 | -5.887718 | | | | |
| Min $S_0$ (explicit water) | | | | Min $S_1$ (explicit water) | | | |
| 6 | -3.242655 | -0.274296 | 0.029889 | 6 | -3.260406 | -0.265439 | -0.014738 |
| 6 | -3.078844 | 0.946026 | -0.752862 | 6 | -3.084411 | 0.939996 | -0.775747 |
| 6 | -3.944604 | 2.062089 | -0.546995 | 6 | -3.922272 | 2.076239 | -0.556950 |
| 6 | -3.534577 | 3.068979 | -1.396641 | 6 | -3.490833 | 3.086374 | -1.380949 |
| 7 | -2.168908 | 1.260826 | -1.727440 | 7 | -2.158411 | 1.262902 | -1.743473 |
| 6 | -2.431767 | 2.570738 | -2.143671 | 6 | -2.400158 | 2.573136 | -2.138474 |
| 5 | -1.013812 | 0.400469 | -2.318292 | 5 | -1.038080 | 0.391832 | -2.341042 |
| 7 | -0.392020 | 1.137773 | -3.537710 | 7 | -0.398207 | 1.124781 | -3.533567 |
| 9 | -1.507348 | -0.854680 | -2.715958 | 9 | -1.557360 | -0.847640 | -2.755981 |
| 9 | -0.013822 | 0.228210 | -1.323462 | 9 | -0.047078 | 0.157652 | -1.348903 |
| 6 | -1.718335 | 3.160303 | -3.192703 | 6 | -1.674791 | 3.178685 | -3.178036 |
| 6 | -0.733454 | 2.447585 | -3.886274 | 6 | -0.703448 | 2.435964 | -3.870736 |
| 6 | -2.018288 | 4.579951 | -3.596572 | 6 | -1.954014 | 4.584385 | -3.581669 |
| 6 | 0.538015 | 0.698574 | -4.443798 | 6 | 0.523021 | 0.662450 | -4.447464 |
| 6 | 0.010560 | 2.819489 | -5.036847 | 6 | 0.061449 | 2.808886 | -5.010921 |
| 6 | 0.788285 | 1.734631 | -5.391956 | 6 | 0.802580 | 1.711836 | -5.376579 |
| 6 | 1.239588 | -0.577812 | -4.535575 | 6 | 1.192294 | -0.604600 | -4.540320 |
| 8 | -1.005827 | 5.433698 | -2.981672 | 8 | -0.942990 | 5.446700 | -2.973428 |
| 1 | -3.000885 | 4.902917 | -3.255201 | 1 | -2.933777 | 4.921246 | -3.246210 |
| 1 | -1.968628 | 4.711935 | -4.678599 | 1 | -1.896143 | 4.721174 | -4.662832 |
| 6 | -1.046729 | 6.755353 | -3.320032 | 6 | -0.997587 | 6.758973 | -3.286893 |
| 6 | 0.112516 | 7.505878 | -2.811818 | 6 | 0.154331 | 7.516190 | -2.778777 |
| 8 | -1.960003 | 7.218309 | -3.988576 | 8 | -1.916102 | 7.224021 | -3.939728 |
| 6 | 0.061792 | 8.955184 | -2.632763 | 6 | 0.088724 | 8.955186 | -2.581042 |
| 6 | 1.277974 | 6.816004 | -2.532149 | 6 | 1.322677 | 6.839297 | -2.515711 |
| 7 | 2.417305 | 7.383248 | -2.093710 | 7 | 2.453134 | 7.410127 | -2.077753 |
| 1 | 1.328895 | 5.745186 | -2.674315 | 1 | 1.382713 | 5.770757 | -2.670470 |
| 6 | 1.340497 | 9.553562 | -2.205060 | 6 | 1.358696 | 9.560992 | -2.148429 |
| 8 | -0.952730 | 9.654698 | -2.804742 | 8 | -0.926953 | 9.644137 | -2.739871 |
| 6 | 2.492326 | 8.779557 | -1.947019 | 6 | 2.510836 | 8.799472 | -1.909038 |
| 6 | 3.598826 | 6.553164 | -1.884649 | 6 | 3.642828 | 6.598130 | -1.889095 |
| 6 | 3.457320 | 5.204392 | -1.230874 | 6 | 3.518436 | 5.246857 | -1.252469 |
| 6 | 4.123579 | 6.335940 | -0.487117 | 6 | 4.169257 | 6.373764 | -0.501300 |
| 1 | 4.325398 | 6.653633 | -2.687476 | 1 | 4.364179 | 6.718264 | -2.692441 |
| 1 | 3.564801 | 6.785529 | 0.328959 | 1 | 3.605998 | 6.811017 | 0.317313 |
| 1 | 5.201884 | 6.328367 | -0.356041 | 1 | 5.246576 | 6.376773 | -0.370235 |
| 1 | 4.071951 | 4.404231 | -1.632686 | 1 | 4.143993 | 4.460320 | -1.661537 |
| 1 | 2.475099 | 4.896400 | -0.882813 | 1 | 2.539084 | 4.923798 | -0.911609 |
| 6 | 1.430191 | 10.949496 | -2.043871 | 6 | 1.428214 | 10.950530 | -1.965649 |
| 6 | 2.612088 | 11.527739 | -1.661119 | 6 | 2.598889 | 11.532328 | -1.576708 |
| 6 | 3.799772 | 10.785561 | -1.426986 | 6 | 3.788363 | 10.800319 | -1.357830 |
| 6 | 3.700665 | 9.395880 | -1.577787 | 6 | 3.709743 | 9.421259 | -1.531329 |
| 1 | 0.549178 | 11.561007 | -2.205279 | 1 | 0.539507 | 11.552331 | -2.116705 |
| 1 | 4.577653 | 8.785350 | -1.422238 | 1 | 4.592973 | 8.818584 | -1.384847 |
| 9 | 2.634358 | 12.877101 | -1.458830 | 9 | 2.614055 | 12.871606 | -1.354800 |

| | | | | | | | |
|---|---|---|---|---|---|---|---|
| 7 | 4.970367 | 11.426995 | -1.010791 | 7 | 4.948581 | 11.452725 | -0.936951 |
| 6 | 5.510871 | 12.487558 | -1.886434 | 6 | 5.471541 | 12.503646 | -1.821889 |
| 6 | 6.044531 | 10.598445 | -0.455049 | 6 | 6.027473 | 10.628298 | -0.402782 |
| 6 | 6.494214 | 13.369748 | -1.122553 | 6 | 6.459245 | 13.386798 | -1.077507 |
| 1 | 4.697797 | 13.106601 | -2.263230 | 1 | 4.652390 | 13.118967 | -2.189623 |
| 1 | 6.020721 | 12.025506 | -2.748449 | 1 | 5.970922 | 12.040292 | -2.687873 |
| 6 | 7.035284 | 11.463529 | 0.327123 | 6 | 7.022881 | 11.490877 | 0.362487 |
| 1 | 6.581473 | 10.052941 | -1.249965 | 1 | 6.553932 | 10.088393 | -1.206978 |
| 1 | 5.612624 | 9.867406 | 0.233504 | 1 | 5.608622 | 9.893228 | 0.288084 |
| 7 | 7.540425 | 12.553912 | -0.508178 | 7 | 7.509828 | 12.576269 | -0.479041 |
| 1 | 7.867296 | 10.846118 | 0.660436 | 1 | 7.861271 | 10.877973 | 0.684959 |
| 1 | 6.534258 | 11.881594 | 1.213116 | 1 | 6.533773 | 11.909148 | 1.253123 |
| 1 | 5.958241 | 13.932834 | -0.343473 | 1 | 5.933556 | 13.948564 | -0.292587 |
| 1 | 6.960369 | 14.080724 | -1.804605 | 1 | 6.915218 | 14.097139 | -1.765388 |
| 6 | 8.825717 | 13.023912 | -0.469269 | 6 | 8.789652 | 13.040717 | -0.464689 |
| 8 | 9.196157 | 14.038525 | -1.061803 | 8 | 9.151008 | 14.049536 | -1.062957 |
| 8 | 9.622688 | 12.228919 | 0.277093 | 8 | 9.596954 | 12.254223 | 0.263990 |
| 6 | 11.075028 | 12.479035 | 0.431394 | 6 | 11.037741 | 12.510594 | 0.390764 |
| 6 | 11.512156 | 11.318918 | 1.330393 | 6 | 11.499425 | 11.362368 | 1.280738 |
| 6 | 11.307165 | 13.820631 | 1.133313 | 6 | 11.274359 | 13.849915 | 1.080757 |
| 6 | 11.771982 | 12.397161 | -0.930052 | 6 | 11.707846 | 12.429774 | -0.976677 |
| 1 | 11.536615 | 11.447760 | -1.424534 | 1 | 11.467157 | 11.479185 | -1.463489 |
| 1 | 12.857086 | 12.442513 | -0.782769 | 1 | 12.793743 | 12.479909 | -0.849029 |
| 1 | 11.475449 | 13.220389 | -1.582886 | 1 | 11.395003 | 13.249591 | -1.624130 |
| 1 | 10.746102 | 13.862472 | 2.073885 | 1 | 10.729427 | 13.890990 | 2.029353 |
| 1 | 11.007045 | 14.660423 | 0.503741 | 1 | 10.958502 | 14.684180 | 0.453657 |
| 1 | 12.372438 | 13.924361 | 1.369311 | 1 | 12.341587 | 13.959433 | 1.297712 |
| 1 | 10.983369 | 11.349241 | 2.289248 | 1 | 10.988428 | 11.392838 | 2.247737 |
| 1 | 12.587431 | 11.386819 | 1.526541 | 1 | 12.576414 | 11.438184 | 1.455842 |
| 1 | 11.308699 | 10.355949 | 0.849518 | 1 | 11.292761 | 10.398148 | 0.806700 |
| 1 | -3.977585 | 4.051341 | -1.491773 | 1 | -3.906634 | 4.081241 | -1.459006 |
| 1 | -0.041053 | 3.773675 | -5.544641 | 1 | 0.042845 | 3.774885 | -5.496601 |
| 6 | -5.059548 | 2.004304 | 0.452385 | 6 | -5.016093 | 2.043262 | 0.456497 |
| 6 | -5.604931 | 0.568657 | 0.503741 | 6 | -5.581032 | 0.624351 | 0.535616 |
| 1 | -5.855537 | 2.709968 | 0.192330 | 1 | -5.802850 | 2.760482 | 0.205978 |
| 1 | -4.690596 | 2.291180 | 1.448921 | 1 | -4.619482 | 2.335418 | 1.439302 |
| 6 | -4.494521 | -0.447930 | 0.685085 | 6 | -4.493460 | -0.410520 | 0.687151 |
| 1 | -6.132023 | 0.356647 | -0.439103 | 1 | -6.135137 | 0.414240 | -0.390325 |
| 1 | -6.341091 | 0.464591 | 1.307234 | 1 | -6.295683 | 0.537872 | 1.358367 |
| 6 | -2.247506 | -1.258963 | 0.223782 | 6 | -2.296676 | -1.293508 | 0.125269 |
| 6 | -2.478872 | -2.375629 | 1.010466 | 6 | -2.536479 | -2.400992 | 0.906022 |
| 6 | -3.724149 | -2.549908 | 1.638054 | 6 | -3.756508 | -2.534677 | 1.585453 |
| 6 | -4.722095 | -1.579427 | 1.473099 | 6 | -4.724696 | -1.532381 | 1.470780 |
| 8 | -3.862356 | -3.675599 | 2.388613 | 8 | -3.903802 | -3.652368 | 2.326778 |
| 6 | -5.105269 | -3.915182 | 3.058578 | 6 | -5.116178 | -3.850537 | 3.048994 |
| 1 | -4.976560 | -4.862145 | 3.583425 | 1 | -5.000673 | -4.803550 | 3.563093 |
| 1 | -5.927758 | -4.000629 | 2.339689 | 1 | -5.971740 | -3.900702 | 2.368528 |
| 1 | -5.321288 | -3.119866 | 3.780626 | 1 | -5.268008 | -3.052693 | 3.782357 |
| 1 | -5.686759 | -1.690547 | 1.955853 | 1 | -5.673077 | -1.619236 | 1.987637 |
| 1 | -1.273667 | -1.137518 | -0.228516 | 1 | -1.339921 | -1.201786 | -0.366138 |
| 1 | -1.702845 | -3.120735 | 1.159283 | 1 | -1.786868 | -3.177939 | 1.016456 |
| 6 | 1.752217 | 1.536613 | -6.522475 | 6 | 1.788607 | 1.523128 | -6.480101 |
| 6 | 1.854040 | -0.879609 | -5.790690 | 6 | 1.861158 | -0.893279 | -5.773441 |
| 6 | 1.399980 | -1.495816 | -3.481548 | 6 | 1.278770 | -1.559385 | -3.506203 |
| 6 | 2.116286 | -2.681109 | -3.638921 | 6 | 1.978637 | -2.743883 | -3.659609 |
| 6 | 2.700331 | -2.974358 | -4.879109 | 6 | 2.623093 | -3.011642 | -4.872268 |
| 6 | 2.564643 | -2.063899 | -5.940807 | 6 | 2.556077 | -2.076168 | -5.914876 |
| 8 | 3.419671 | -4.097424 | -5.148811 | 8 | 3.331968 | -4.130656 | -5.128953 |
| 6 | 3.602549 | -5.065484 | -4.109463 | 6 | 3.441911 | -5.125740 | -4.114623 |

| | | | | | | | |
|---|---|---|---|---|---|---|---|
| 1 | 4.194874 | -5.865099 | -4.555105 | 1 | 4.044153 | -5.923062 | -4.547566 |
| 1 | 2.639537 | -5.464084 | -3.771341 | 1 | 2.456142 | -5.514854 | -3.842244 |
| 1 | 4.145708 | -4.632656 | -3.261869 | 1 | 3.943588 | -4.725124 | -3.228428 |
| 1 | 0.979413 | -1.278893 | -2.509784 | 1 | 0.812286 | -1.359759 | -2.553201 |
| 1 | 2.218397 | -3.352715 | -2.794484 | 1 | 2.025707 | -3.441637 | -2.832759 |
| 1 | 3.031117 | -2.304899 | -6.892514 | 1 | 3.061926 | -2.307404 | -6.847695 |
| 6 | 1.701066 | 0.065903 | -6.965873 | 6 | 1.756792 | 0.063440 | -6.935467 |
| 1 | 0.731091 | -0.122081 | -7.451022 | 1 | 0.806128 | -0.121604 | -7.455094 |
| 1 | 2.473921 | -0.139754 | -7.713371 | 1 | 2.555297 | -0.135702 | -7.655035 |
| 1 | 2.774203 | 1.785637 | -6.198257 | 1 | 2.797296 | 1.774063 | -6.122209 |
| 1 | 1.513753 | 2.200194 | -7.360403 | 1 | 1.572341 | 2.194923 | -7.315627 |
| 8 | -0.017683 | 4.713749 | -0.182983 | 8 | 0.201950 | 4.586197 | -0.385996 |
| 1 | -0.372611 | 3.865953 | 0.128717 | 1 | -0.123463 | 3.735090 | -0.056932 |
| 1 | -0.455041 | 4.864609 | -1.040421 | 1 | -0.305162 | 4.757408 | -1.199217 |
| 8 | -4.583312 | 6.915300 | -5.103545 | 8 | -4.423101 | 6.601558 | -5.084138 |
| 1 | -3.689806 | 7.005909 | -4.716386 | 1 | -3.558597 | 6.827222 | -4.687726 |
| 1 | -4.893917 | 7.824200 | -5.237534 | 1 | -4.834825 | 7.448102 | -5.309767 |
| Min $T_1$ (explicit water) | | | | TS $T_1$ (explicit water) | | | |
| 6 | -3.320454 | -0.305099 | 0.001474 | 6 | -3.105537 | -0.274838 | 0.031059 |
| 6 | -3.120015 | 0.902570 | -0.758998 | 6 | -3.123539 | 0.881277 | -0.825908 |
| 6 | -3.939083 | 2.058714 | -0.542592 | 6 | -4.127009 | 1.899529 | -0.676082 |
| 6 | -3.487663 | 3.060938 | -1.370415 | 6 | -3.862910 | 2.886084 | -1.598226 |
| 7 | -2.176205 | 1.216049 | -1.739181 | 7 | -2.271316 | 1.262615 | -1.854264 |
| 6 | -2.398481 | 2.525741 | -2.131483 | 6 | -2.717121 | 2.474585 | -2.337968 |
| 5 | -1.067262 | 0.323221 | -2.344207 | 5 | -1.031852 | 0.515067 | -2.418447 |
| 7 | -0.408186 | 1.058432 | -3.534613 | 7 | -0.523184 | 1.246864 | -3.690389 |
| 9 | -1.612536 | -0.906226 | -2.774289 | 9 | -1.376755 | -0.809783 | -2.734452 |
| 9 | -0.078830 | 0.063449 | -1.348010 | 9 | -0.004159 | 0.522866 | -1.440552 |
| 6 | -1.660554 | 3.132716 | -3.172475 | 6 | -2.101083 | 3.121173 | -3.458995 |
| 6 | -0.696452 | 2.369270 | -3.869109 | 6 | -1.015809 | 2.460162 | -4.122063 |
| 6 | -1.929785 | 4.539757 | -3.578635 | 6 | -2.539114 | 4.380284 | -3.888016 |
| 6 | 0.521011 | 0.581354 | -4.461441 | 6 | 0.458571 | 0.848859 | -4.589071 |
| 6 | 0.077862 | 2.741142 | -5.014982 | 6 | -0.329051 | 2.855328 | -5.305833 |
| 6 | 0.810831 | 1.637634 | -5.387407 | 6 | 0.569239 | 1.858350 | -5.606776 |
| 6 | 1.177335 | -0.696761 | -4.560747 | 6 | 1.304384 | -0.315482 | -4.604245 |
| 8 | -0.921109 | 5.423839 | -2.949433 | 8 | -1.447235 | 5.737926 | -2.864809 |
| 1 | -2.913177 | 4.882942 | -3.258372 | 1 | -3.472675 | 4.783773 | -3.522499 |
| 1 | -1.849576 | 4.686222 | -4.657711 | 1 | -2.218006 | 4.771916 | -4.843157 |
| 6 | -0.983337 | 6.742443 | -3.266582 | 6 | -1.205500 | 6.864518 | -3.463220 |
| 6 | 0.172166 | 7.507775 | -2.761654 | 6 | -0.021494 | 7.621059 | -2.931287 |
| 8 | -1.907298 | 7.211255 | -3.920292 | 8 | -1.856982 | 7.301635 | -4.433958 |
| 6 | 0.093400 | 8.947983 | -2.532822 | 6 | 0.034609 | 9.075445 | -2.889215 |
| 6 | 1.359981 | 6.838084 | -2.537156 | 6 | 1.048506 | 6.883821 | -2.477958 |
| 7 | 2.499155 | 7.417548 | -2.110629 | 7 | 2.207459 | 7.403332 | -2.005258 |
| 1 | 1.431173 | 5.774092 | -2.716983 | 1 | 1.004306 | 5.802506 | -2.491362 |
| 6 | 1.368657 | 9.562676 | -2.117575 | 6 | 1.324364 | 9.619486 | -2.420741 |
| 8 | -0.941257 | 9.629240 | -2.654386 | 8 | -0.908328 | 9.833627 | -3.196449 |
| 6 | 2.545075 | 8.808717 | -1.916959 | 6 | 2.389523 | 8.792422 | -1.999442 |
| 6 | 3.706224 | 6.610884 | -1.968853 | 6 | 3.293876 | 6.511092 | -1.625513 |
| 6 | 3.618365 | 5.235263 | -1.363510 | 6 | 2.995637 | 5.260496 | -0.841934 |
| 6 | 4.280168 | 6.354538 | -0.597422 | 6 | 3.712886 | 6.410518 | -0.179173 |
| 1 | 4.405140 | 6.759228 | -2.788692 | 1 | 4.075989 | 6.454217 | -2.379636 |
| 1 | 3.736966 | 6.758598 | 0.252307 | 1 | 3.148313 | 6.994019 | 0.542599 |
| 1 | 5.362001 | 6.369332 | -0.499999 | 1 | 4.775825 | 6.326795 | 0.028660 |
| 1 | 4.239678 | 4.467006 | -1.814412 | 1 | 3.557822 | 4.370730 | -1.109339 |
| 1 | 2.655138 | 4.889037 | -0.998718 | 1 | 1.971198 | 5.072402 | -0.534550 |
| 6 | 1.429461 | 10.954164 | -1.910010 | 6 | 1.516169 | 11.015032 | -2.386256 |
| 6 | 2.606884 | 11.547898 | -1.537590 | 6 | 2.710846 | 11.541396 | -1.971372 |

| | | | | | | | |
|---|---|---|---|---|---|---|---|
| 6 | 3.817323 | 10.827402 | -1.359257 | 6 | 3.816890 | 10.741662 | -1.579423 |
| 6 | 3.747829 | 9.442105 | -1.556950 | 6 | 3.617782 | 9.356205 | -1.603262 |
| 1 | 0.530020 | 11.548705 | -2.027288 | 1 | 0.700001 | 11.669657 | -2.672203 |
| 1 | 4.642857 | 8.848384 | -1.444935 | 1 | 4.430545 | 8.702412 | -1.323482 |
| 9 | 2.602895 | 12.889939 | -1.289147 | 9 | 2.827584 | 12.900857 | -1.896758 |
| 7 | 4.982288 | 11.483474 | -0.948522 | 7 | 5.005969 | 11.337060 | -1.139161 |
| 6 | 5.477727 | 12.582715 | -1.802574 | 6 | 5.686485 | 12.255337 | -2.075440 |
| 6 | 6.087857 | 10.663980 | -0.443569 | 6 | 5.971435 | 10.488613 | -0.434515 |
| 6 | 6.457481 | 13.464466 | -1.033679 | 6 | 6.692893 | 13.132140 | -1.335125 |
| 1 | 4.641919 | 13.193463 | -2.141458 | 1 | 4.952778 | 12.894119 | -2.565463 |
| 1 | 5.978065 | 12.159826 | -2.689948 | 1 | 6.207334 | 11.669660 | -2.851781 |
| 6 | 7.075011 | 11.527985 | 0.344263 | 6 | 6.984338 | 11.348242 | 0.325242 |
| 1 | 6.619932 | 10.157072 | -1.266904 | 1 | 6.507162 | 9.824024 | -1.134353 |
| 1 | 5.688981 | 9.901110 | 0.230284 | 1 | 5.439014 | 9.867287 | 0.290524 |
| 7 | 7.535813 | 12.655754 | -0.466926 | 7 | 7.628568 | 12.304207 | -0.576120 |
| 1 | 7.928606 | 10.920889 | 0.639876 | 1 | 7.739616 | 10.705064 | 0.772932 |
| 1 | 6.584111 | 11.905936 | 1.253634 | 1 | 6.469107 | 11.893683 | 1.130064 |
| 1 | 5.926149 | 13.991173 | -0.226425 | 1 | 6.159999 | 13.809628 | -0.650828 |
| 1 | 6.891784 | 14.206423 | -1.703639 | 1 | 7.256568 | 13.733538 | -2.048278 |
| 6 | 8.808503 | 13.159692 | -0.433437 | 6 | 8.949186 | 12.657643 | -0.515529 |
| 8 | 9.141992 | 14.201015 | -1.000992 | 8 | 9.440928 | 13.566422 | -1.186623 |
| 8 | 9.638364 | 12.365397 | 0.276872 | 8 | 9.633254 | 11.878262 | 0.350254 |
| 6 | 11.085702 | 12.650263 | 0.416607 | 6 | 11.095274 | 12.008906 | 0.552013 |
| 6 | 11.567127 | 11.478035 | 1.276393 | 6 | 11.380777 | 10.915079 | 1.585128 |
| 6 | 11.293611 | 13.977674 | 1.152270 | 6 | 11.427720 | 13.389974 | 1.125201 |
| 6 | 11.763175 | 12.624706 | -0.956846 | 6 | 11.836818 | 11.719905 | -0.756780 |
| 1 | 11.545077 | 11.683432 | -1.474246 | 1 | 11.529279 | 10.750504 | -1.165383 |
| 1 | 12.848902 | 12.694921 | -0.824596 | 1 | 12.913906 | 11.677613 | -0.558631 |
| 1 | 11.434941 | 13.457555 | -1.581771 | 1 | 11.651244 | 12.494710 | -1.503263 |
| 1 | 10.745943 | 13.978851 | 2.101621 | 1 | 10.837102 | 13.581984 | 2.028274 |
| 1 | 10.962510 | 14.826356 | 0.550811 | 1 | 11.234664 | 14.182679 | 0.399958 |
| 1 | 12.359330 | 14.102506 | 1.375725 | 1 | 12.488092 | 13.419713 | 1.400905 |
| 1 | 11.052017 | 11.467515 | 2.243091 | 1 | 10.816373 | 11.093938 | 2.506763 |
| 1 | 12.642960 | 11.569841 | 1.459141 | 1 | 12.448138 | 10.905748 | 1.830017 |
| 1 | 11.382351 | 10.523530 | 0.771704 | 1 | 11.107212 | 9.929160 | 1.193863 |
| 1 | -3.883005 | 4.064269 | -1.449286 | 1 | -4.427463 | 3.794856 | -1.754750 |
| 1 | 0.069603 | 3.710505 | -5.494488 | 1 | -0.500695 | 3.764057 | -5.865828 |
| 6 | -5.040016 | 2.046350 | 0.469839 | 6 | -5.195401 | 1.786169 | 0.362270 |
| 6 | -5.635080 | 0.631975 | 0.548775 | 6 | -5.539718 | 0.303211 | 0.569957 |
| 1 | -5.813069 | 2.779653 | 0.217674 | 1 | -6.084092 | 2.355813 | 0.072842 |
| 1 | -4.640378 | 2.330686 | 1.455284 | 1 | -4.835520 | 2.214554 | 1.309812 |
| 6 | -4.564106 | -0.427584 | 0.702564 | 6 | -4.302361 | -0.544626 | 0.773410 |
| 1 | -6.194933 | 0.434594 | -0.378257 | 1 | -6.073999 | -0.062815 | -0.319666 |
| 1 | -6.353010 | 0.561552 | 1.371950 | 1 | -6.219514 | 0.181788 | 1.418622 |
| 6 | -2.374703 | -1.355021 | 0.145344 | 6 | -1.990793 | -1.133469 | 0.230278 |
| 6 | -2.638562 | -2.462850 | 0.928539 | 6 | -2.050383 | -2.198741 | 1.104696 |
| 6 | -3.866882 | -2.574955 | 1.607899 | 6 | -3.235167 | -2.459810 | 1.824040 |
| 6 | -4.818485 | -1.549476 | 1.489313 | 6 | -4.351247 | -1.624638 | 1.650754 |
| 8 | -4.035184 | -3.697113 | 2.351832 | 8 | -3.197509 | -3.523167 | 2.656471 |
| 6 | -5.258971 | -3.878441 | 3.075819 | 6 | -4.357459 | -3.849310 | 3.436985 |
| 1 | -5.156023 | -4.835954 | 3.586625 | 1 | -4.077863 | -4.728361 | 4.017283 |
| 1 | -6.115024 | -3.913702 | 2.393224 | 1 | -5.207185 | -4.087309 | 2.788795 |
| 1 | -5.399074 | -3.079752 | 3.812266 | 1 | -4.615265 | -3.025869 | 4.111035 |
| 1 | -5.770469 | -1.618172 | 2.003912 | 1 | -5.269802 | -1.812300 | 2.195116 |
| 1 | -1.414358 | -1.282575 | -0.343332 | 1 | -1.062650 | -0.938741 | -0.285945 |
| 1 | -1.901655 | -3.252923 | 1.039344 | 1 | -1.188352 | -2.840013 | 1.261036 |
| 6 | 1.794266 | 1.443694 | -6.497883 | 6 | 1.555346 | 1.728798 | -6.722416 |
| 6 | 1.843340 | -0.987312 | -5.802301 | 6 | 1.988662 | -0.599894 | -5.837728 |
| 6 | 1.258566 | -1.661026 | -3.527946 | 6 | 1.550300 | -1.164739 | -3.498294 |

| | | | | | | | |
|---|---|---|---|---|---|---|---|
| 6 | 1.950157 | -2.855851 | -3.689114 | 6 | 2.415342 | -2.246036 | -3.581492 |
| 6 | 2.592396 | -3.126238 | -4.908577 | 6 | 3.071561 | -2.516386 | -4.795971 |
| 6 | 2.530410 | -2.180814 | -5.950558 | 6 | 2.848025 | -1.682643 | -5.909238 |
| 8 | 3.295149 | -4.255114 | -5.176700 | 8 | 3.933371 | -3.538858 | -4.990875 |
| 6 | 3.401611 | -5.265044 | -4.164700 | 6 | 4.214305 | -4.437045 | -3.906645 |
| 1 | 3.996870 | -6.062259 | -4.610140 | 1 | 4.922646 | -5.161851 | -4.307404 |
| 1 | 2.412917 | -5.649501 | -3.891898 | 1 | 3.302997 | -4.949400 | -3.581308 |
| 1 | 3.910845 | -4.875360 | -3.276588 | 1 | 4.667354 | -3.901300 | -3.066069 |
| 1 | 0.796279 | -1.462325 | -2.572186 | 1 | 1.080592 | -0.958739 | -2.548014 |
| 1 | 1.992564 | -3.557283 | -2.864048 | 1 | 2.582220 | -2.859458 | -2.704066 |
| 1 | 3.031485 | -2.411586 | -6.886968 | 1 | 3.364843 | -1.912204 | -6.836923 |
| 6 | 1.746746 | -0.020027 | -6.963212 | 6 | 1.731075 | 0.242097 | -7.068948 |
| 1 | 0.793120 | -0.190636 | -7.485583 | 1 | 0.811814 | -0.117102 | -7.555299 |
| 1 | 2.543700 | -0.221430 | -7.685812 | 1 | 2.543514 | 0.107862 | -7.789450 |
| 1 | 2.807850 | 1.681596 | -6.140547 | 1 | 2.523028 | 2.149124 | -6.409479 |
| 1 | 1.583929 | 2.123286 | -7.330249 | 1 | 1.230262 | 2.297729 | -7.599223 |
| 8 | 0.187638 | 4.561603 | -0.285586 | 8 | -1.283965 | 5.224777 | -0.095313 |
| 1 | -0.093024 | 3.668187 | -0.031493 | 1 | -2.026843 | 4.622994 | 0.069965 |
| 1 | -0.267173 | 4.734644 | -1.130944 | 1 | -1.323695 | 5.412629 | -1.060418 |
| 8 | -4.485088 | 6.825510 | -5.085980 | 8 | -4.566623 | 6.996415 | -4.933708 |
| 1 | -3.602249 | 6.937177 | -4.678349 | 1 | -3.600599 | 7.085533 | -4.747449 |
| 1 | -4.808192 | 7.726865 | -5.239716 | 1 | -4.892697 | 7.905269 | -5.021711 |
| Products $T_1$ (explicit water) | | | | | | | |
| 6 | -3.976302 | -4.992583 | 1.349040 | | | | |
| 6 | -3.907512 | -4.092217 | 0.235403 | | | | |
| 6 | -4.570659 | -2.809539 | 0.271549 | | | | |
| 6 | -4.286334 | -2.158414 | -0.903964 | | | | |
| 7 | -3.249659 | -4.204409 | -0.975949 | | | | |
| 6 | -3.480142 | -3.039057 | -1.675989 | | | | |
| 5 | -2.405436 | -5.392174 | -1.523538 | | | | |
| 7 | -2.071317 | -5.133478 | -3.024238 | | | | |
| 9 | -3.129865 | -6.584671 | -1.404116 | | | | |
| 9 | -1.199298 | -5.474857 | -0.791738 | | | | |
| 6 | -2.995407 | -2.844681 | -3.021746 | | | | |
| 6 | -2.342180 | -3.951525 | -3.679358 | | | | |
| 6 | -3.187048 | -1.646062 | -3.669907 | | | | |
| 6 | -1.514093 | -5.999782 | -3.946805 | | | | |
| 6 | -1.948297 | -4.056236 | -5.041137 | | | | |
| 6 | -1.450760 | -5.323358 | -5.221424 | | | | |
| 6 | -1.016258 | -7.335425 | -3.795984 | | | | |
| 8 | -3.011816 | 1.841320 | 0.797526 | | | | |
| 1 | -3.701367 | -0.816206 | -3.195933 | | | | |
| 1 | -2.827952 | -1.493432 | -4.680983 | | | | |
| 6 | -2.522410 | 2.550587 | -0.135208 | | | | |
| 6 | -1.344795 | 3.441861 | 0.209997 | | | | |
| 8 | -2.916053 | 2.539437 | -1.337325 | | | | |
| 6 | -1.211080 | 4.808841 | -0.263958 | | | | |
| 6 | -0.368224 | 2.906572 | 1.012141 | | | | |
| 7 | 0.773895 | 3.543090 | 1.389437 | | | | |
| 1 | -0.471732 | 1.899147 | 1.395133 | | | | |
| 6 | 0.061167 | 5.461855 | 0.100371 | | | | |
| 8 | -2.086355 | 5.424782 | -0.912287 | | | | |
| 6 | 1.035916 | 4.826676 | 0.903769 | | | | |
| 6 | 1.758601 | 2.835278 | 2.192892 | | | | |
| 6 | 1.301984 | 1.984588 | 3.348728 | | | | |
| 6 | 2.030765 | 3.279911 | 3.609452 | | | | |
| 1 | 2.604375 | 2.475792 | 1.610143 | | | | |
| 1 | 1.442133 | 4.109842 | 3.990289 | | | | |

| | | | | |
|---|---|---|---|---|
| 1 | 3.060816 | 3.251991 | 3.953667 | |
| 1 | 1.825620 | 1.045405 | 3.501329 | |
| 1 | 0.241318 | 1.956351 | 3.581574 | |
| 6 | 0.331840 | 6.765985 | -0.361886 | |
| 6 | 1.515310 | 7.381287 | -0.051143 | |
| 6 | 2.533369 | 6.760478 | 0.719931 | |
| 6 | 2.256689 | 5.472125 | 1.188888 | |
| 1 | -0.416037 | 7.285460 | -0.951249 | |
| 1 | 3.001486 | 4.952262 | 1.773490 | |
| 9 | 1.708155 | 8.667462 | -0.474054 | |
| 7 | 3.714156 | 7.452974 | 1.026320 | |
| 6 | 4.531303 | 7.915527 | -0.113980 | |
| 6 | 4.559280 | 6.912696 | 2.094549 | |
| 6 | 5.528408 | 8.980671 | 0.333533 | |
| 1 | 3.885653 | 8.336336 | -0.884059 | |
| 1 | 5.074852 | 7.059720 | -0.549490 | |
| 6 | 5.558449 | 7.969053 | 2.571105 | |
| 1 | 5.108144 | 6.015767 | 1.758460 | |
| 1 | 3.928336 | 6.631733 | 2.942111 | |
| 7 | 6.338805 | 8.486395 | 1.445805 | |
| 1 | 6.229482 | 7.526228 | 3.304771 | |
| 1 | 5.014213 | 8.796374 | 3.050901 | |
| 1 | 4.986618 | 9.886767 | 0.644716 | |
| 1 | 6.189909 | 9.239972 | -0.492992 | |
| 6 | 7.663336 | 8.824115 | 1.515802 | |
| 8 | 8.266412 | 9.401564 | 0.609747 | |
| 8 | 8.216203 | 8.429484 | 2.683536 | |
| 6 | 9.654485 | 8.617960 | 2.984847 | |
| 6 | 9.778425 | 8.001428 | 4.381174 | |
| 6 | 9.993872 | 10.111169 | 3.023931 | |
| 6 | 10.510975 | 7.845003 | 1.977188 | |
| 1 | 10.200848 | 6.794535 | 1.936858 | |
| 1 | 11.559105 | 7.876797 | 2.296023 | |
| 1 | 10.439292 | 8.274857 | 0.976155 | |
| 1 | 9.323108 | 10.638221 | 3.712075 | |
| 1 | 9.916119 | 10.566365 | 2.034771 | |
| 1 | 11.020132 | 10.238019 | 3.387156 | |
| 1 | 9.132944 | 8.523895 | 5.095558 | |
| 1 | 10.813557 | 8.078681 | 4.730238 | |
| 1 | 9.496965 | 6.942892 | 4.365519 | |
| 1 | -4.608334 | -1.169208 | -1.209490 | |
| 1 | -2.045388 | -3.284303 | -5.791572 | |
| 6 | -5.364567 | -2.385560 | 1.462953 | |
| 6 | -6.002721 | -3.623090 | 2.112618 | |
| 1 | -6.128891 | -1.654590 | 1.182344 | |
| 1 | -4.701384 | -1.892249 | 2.189006 | |
| 6 | -5.000429 | -4.736254 | 2.323514 | |
| 1 | -6.804270 | -3.994163 | 1.456829 | |
| 1 | -6.471433 | -3.358629 | 3.064886 | |
| 6 | -3.087688 | -6.080240 | 1.580096 | |
| 6 | -3.203635 | -6.874521 | 2.698149 | |
| 6 | -4.223395 | -6.622456 | 3.643774 | |
| 6 | -5.110417 | -5.549721 | 3.445510 | |
| 8 | -4.259012 | -7.454042 | 4.700210 | |
| 6 | -5.255317 | -7.268425 | 5.720535 | |
| 1 | -5.073340 | -8.059775 | 6.446872 | |
| 1 | -6.260813 | -7.371365 | 5.300920 | |
| 1 | -5.140029 | -6.290407 | 6.197968 | |
| 1 | -5.895937 | -5.345583 | 4.163883 | |

| | | | | |
|---|---|---|---|---|
| 1 | -2.282365 | -6.274295 | 0.887576 | |
| 1 | -2.512806 | -7.692303 | 2.877267 | |
| 6 | -0.889291 | -5.997780 | -6.429518 | |
| 6 | -0.796706 | -8.085266 | -5.007188 | |
| 6 | -0.677206 | -7.945257 | -2.560588 | |
| 6 | -0.155297 | -9.224732 | -2.497326 | |
| 6 | 0.043899 | -9.951297 | -3.689236 | |
| 6 | -0.279763 | -9.364655 | -4.930339 | |
| 8 | 0.542055 | -11.198887 | -3.745617 | |
| 6 | 0.904085 | -11.876624 | -2.529565 | |
| 1 | 1.267437 | -12.854558 | -2.843464 | |
| 1 | 0.031732 | -11.993998 | -1.879457 | |
| 1 | 1.698534 | -11.333838 | -2.008093 | |
| 1 | -0.792201 | -7.393198 | -1.639605 | |
| 1 | 0.105516 | -9.645196 | -1.533654 | |
| 1 | -0.116487 | -9.947364 | -5.832297 | |
| 6 | -1.181626 | -7.504410 | -6.349756 | |
| 1 | -2.258569 | -7.664523 | -6.506050 | |
| 1 | -0.662336 | -8.039453 | -7.149942 | |
| 1 | 0.198430 | -5.836881 | -6.466251 | |
| 1 | -1.305914 | -5.566827 | -7.345032 | |
| 8 | -2.645267 | 2.065254 | 3.497279 | |
| 1 | -3.525763 | 1.902234 | 3.868860 | |
| 1 | -2.764738 | 2.016337 | 2.511419 | |
| 8 | -4.891145 | 0.887528 | -2.186587 | |
| 1 | -4.188821 | 1.488290 | -1.805839 | |
| 1 | -5.723735 | 1.182686 | -1.786804 | |

## 10. References


1. Sitkowska, K.; Feringa, B. L.; Szymański, W., Green-Light-Sensitive BODIPY Photoprotecting Groups for Amines. *The Journal of Organic Chemistry* **2018,** *83* (4), 1819-1827.
2. Peterson, J. A.; Wijesooriya, C.; Gehrmann, E. J.; Mahoney, K. M.; Goswami, P. P.; Albright, T. R.; Syed, A.; Dutton, A. S.; Smith, E. A.; Winter, A. H., Family of BODIPY Photocages Cleaved by Single Photons of Visible/Near-Infrared Light. *J. Am. Chem. Soc.* **2018,** *140* (23), 7343-7346.
3. Megerle, U.; Lechner, R.; König, B.; Riedle, E., Laboratory apparatus for the accurate, facile and rapid determination of visible light photoreaction quantum yields. *Photochem. Photobiol. Sci.* **2010,** *9* (10), 1400-1406.
4. European Comitee on Antimicrobial Suscestibility Testing, Determination of minimum inhibitory concentrations (MICs) of antibacterial agents by broth microdilution, EUCAST Discussion Document E.Def 5.1. *Clin. Microbiol. Infect.* **2003,** *9* (7), 1-10.
5. Becke, A. D., Density-functional thermochemistry. III. The role of exact exchange. The Journal of Chemical Physics 1993, 98 (7), 5648-5652.
6. Lee, C.; Yang, W.; Parr, R. G., Development of the Colle-Salvetti correlation-energy formula into a functional of the electron density. *Phys. Rev. B* **1988**, *37* (2), 785-789.
7. Yanai, T.; Tew, D. P.; Handy, N. C., A new hybrid exchange–correlation functional using the Coulomb-attenuating method (CAM-B3LYP). *Chem. Phys. Lett*. **2004**, *393* (1), 51-57.
8. Tomasi, J.; Mennucci, B.; Cammi, R., Quantum Mechanical Continuum Solvation Models. *Chem. Rev.* **2005**, *105* (8), 2999-3094.

9. Frisch, M. J.; Trucks, G. W.; Schlegel, H. B.; Scuseria, G. E.; Robb, M. A.; Cheeseman, J. R.; Scalmani, G.; Barone, V.; Petersson, G. A.; Nakatsuji, H.; Li, X.; Caricato, M.; Marenich, A. V.; Bloino, J.; Janesko, B. G.; Gomperts, R.; Mennucci, B.; Hratchian, H. P.; Ortiz, J. V.; Izmaylov, A. F.; Sonnenberg, J. L.; Williams; Ding, F.; Lipparini, F.; Egidi, F.; Goings, J.; Peng, B.; Petrone, A.; Henderson, T.; Ranasinghe, D.; Zakrzewski, V. G.; Gao, J.; Rega, N.; Zheng, G.; Liang, W.; Hada, M.; Ehara, M.; Toyota, K.; Fukuda, R.; Hasegawa, J.; Ishida, M.; Nakajima, T.; Honda, Y.; Kitao, O.; Nakai, H.; Vreven, T.; Throssell, K.; Montgomery Jr., J. A.; Peralta, J. E.; Ogliaro, F.; Bearpark, M. J.; Heyd, J. J.; Brothers, E. N.; Kudin, K. N.; Staroverov, V. N.; Keith, T. A.; Kobayashi, R.; Normand, J.; Raghavachari, K.; Rendell, A. P.; Burant, J. C.; Iyengar, S. S.; Tomasi, J.; Cossi, M.; Millam, J. M.; Klene, M.; Adamo, C.; Cammi, R.; Ochterski, J. W.; Martin, R. L.; Morokuma, K.; Farkas, O.; Foresman, J. B.; Fox, D. J. Gaussian 16 Rev. C.01, Wallingford, CT, 2016.

10. Weigend, F.; Ahlrichs, R. Balanced basis sets of split valence, triple zeta valence and quadruple zeta valence quality for H to Rn: Design and assessment of accuracy. *Phys. Chem. Chem. Phys.* **2005**, *7*, 3297-3305.

11. Rappoport, D.; Furche, F. Property-optimized Gaussian basis sets for molecular response calculations. *J. Chem. Phys*., **2010**, *133*, 134105.

12. Neese, F.; Wennmohs, F.; Becker, U.; Riplinger, C. The ORCA quantum chemistry program package. *J. Chem. Phys*., **2020**, *152*, 224108.
